# Serval Optimization Algorithm: A New Bio-Inspired Approach for Solving Optimization Problems

**DOI:** 10.3390/biomimetics7040204

**Published:** 2022-11-20

**Authors:** Mohammad Dehghani, Pavel Trojovský

**Affiliations:** Department of Mathematics, Faculty of Science, University of Hradec Králové, 500 03 Hradec Králové, Czech Republic

**Keywords:** bio-inspired, engineering systems, optimization, metaheuristic, serval, exploration, exploitation

## Abstract

This article introduces a new metaheuristic algorithm called the Serval Optimization Algorithm (SOA), which imitates the natural behavior of serval in nature. The fundamental inspiration of SOA is the serval’s hunting strategy, which attacks the selected prey and then hunts the prey in a chasing process. The steps of SOA implementation in two phases of exploration and exploitation are mathematically modeled. The capability of SOA in solving optimization problems is challenged in the optimization of thirty-nine standard benchmark functions from the CEC 2017 test suite and CEC 2019 test suite. The proposed SOA approach is compared with the performance of twelve well-known metaheuristic algorithms to evaluate further. The optimization results show that the proposed SOA approach, due to the appropriate balancing exploration and exploitation, is provided better solutions for most of the mentioned benchmark functions and has superior performance compared to competing algorithms. SOA implementation on the CEC 2011 test suite and four engineering design challenges shows the high efficiency of the proposed approach in handling real-world optimization applications.

## 1. Introduction

An optimization problem is a type of problem that has several feasible solutions. Optimization is the process of searching for the best solution among possible solutions for a problem [1]. Optimization is used in various science, engineering, technology, and real-world applications [2]. Finding the best optimum can achieve multiple benefits, such as reducing costs, maximizing profits, improving equipment efficiency, etc. For this reason, finding suitable and effective solutions for optimization applications is a fundamental challenge for scientists. From a mathematical point of view, an optimization problem is characterized by three main parts: decision variables, objectives, and constraints [3].

Problem-solving techniques in dealing with optimization tasks are classified into two groups: deterministic and stochastic approaches [4]. Deterministic methods in two categories, gradient-based and non-gradient-based, are effective in handling optimization problems that are linear, convex, differentiable, and have a continuous search space [5]. Among the deterministic methods of solving optimization problems are approaches such as dynamic programming, Newton methods, linear programming, gradient method, quadratic programming, and simplex methods, among the deterministic methods of solving optimization problems [6]. However, as optimization problems become more complex, the number of decision variables increases, and for many real-world applications, deterministic approaches lose their effectiveness. Features such as complexity, non-linearity, non-convexity, non-differentiability, discretization, and problems with high-dimensions objective functions or a discrete search space, etc., are the nature of many modern optimization problems and real-world applications. Such characteristics lead to disruption of the efficiency of deterministic approaches and the problem that they are getting stuck in local optima [7]. Such difficulties in deterministic approaches have led researchers to develop new methods called stochastic approaches to deal with optimization problems. Advantages such as simplicity of concepts, easy implementation, no dependence on the type of problem, no need for derivative information, efficiency in non-linear, non-convex, NP-hard, complex and high-dimensional problems, efficiency in non-linear, and unknown search spaces have made metaheuristic algorithms popular and widespread [8].

The process of solution finding in metaheuristic algorithms starts with the random generation of a number of feasible solutions in the search space. Then, these solutions are improved during iterations of the algorithm based on different steps of updating the metaheuristic algorithms. Finally, after the full implementation of the algorithm, the best feasible solution found during the iterations is presented as a solution to the problem [9]. Metaheuristic algorithms perform the search process in the problem-solving space at both global and local levels. Global search with the concept of exploration ability leads to scanning different regions of the problem-solving space and avoiding getting stuck in local optima. Local search with the concept of exploitation ability leads to finding better solutions in promising areas of search space. In addition to the appropriate quality in exploration and exploitation, the primary key to the success of metaheuristic algorithms in solving optimization problems is to create a balance between exploration and exploitation during algorithm iterations [10].

The nature of random search in metaheuristic algorithms leads to the fact that there is no guarantee that the solutions obtained from these methods are the best solution to the problem. However, these solutions are acceptable as quasi-optimal solutions. A metaheuristic algorithm that can provide better pseudo-optimal solutions closer to the global optimum has a superiority in competition with other metaheuristic algorithms. The desire of scientists to achieve better and more effective solutions for optimization applications has led to the introduction of numerous metaheuristic algorithms [11].

Due to the fact that numerous metaheuristic algorithms have been developed so far, the main research question is: Does the world still need to introduce newer metaheuristic algorithms? No Free Lunch (NFL) [12] theorem answers the question that the effective performance of a metaheuristic algorithm in solving a set of optimization problems does not guarantee the same performance of that algorithm in all optimization applications. According to the NFL theorem, there is no assumption about the efficiency or non-efficiency of an algorithm in handling an optimization problem. Therefore, it can only be claimed that a particular algorithm is the best optimizer for some optimization tasks. Instead, the NFL theorem encourages and motivates researchers to be able to provide more effective solutions for optimization tasks by designing new metaheuristic algorithms. This theorem has also inspired the authors of this article to develop a new metaheuristic algorithm to deal with optimization problems.

According to the concept of the NFL theorem, the randomness of the search process in metaheuristic algorithms, the failure to guarantee the achievement of the global optimal by metaheuristic algorithms, and failure of a metaheuristic algorithm to provide similar performance in all optimization applications, the world always needs to introduce newer metaheuristic algorithms to provide more effective solutions for optimization problems. In this regard, the goal of the paper is to introduce a new metaheuristic algorithm to provide an effective problem-solving tool for researchers to be able to achieve better solutions for optimization tasks. The proposed new metaheuristic algorithm is developed based on simulating the natural behavior of serval during hunting and chasing process. In the proposed method, by simulating the search process in two phases of (i) exploration with the aim of increasing the global search power of the algorithm in order to identify the main optimal area and prevent getting stuck in the local optimal and the (ii) exploitation with the aim of increasing the local search power in order to achieve better solutions, it is expected acquired more effective solutions that are closer to the global optimum in solving optimization problems.

This paper’s novelty and innovative aspects in designing a new optimizer called the Serval Optimization Algorithm (SOA) to deal with optimization tasks in different sciences. The main contributions of this paper are listed as follows:

- SOA is a nature-inspired approach that simulates natural serval behaviors.
- The essential inspiration of SOA is the serval strategy when hunting in three stages: selection, attack, and chase.
- The mathematical model of SOA is presented in two phases: exploration and exploitation.
- SOA capability is benchmarked in optimizing the CEC 2017 and CEC 2019 test suites.
- The performance of SOA in handling real-world applications is evaluated on the CEC 2011 test suite and four engineering design challenges.
- The performance of the proposed SOA approach is challenged in comparison with twelve well-known metaheuristic algorithms.

The article is organized as follows: a literature review is presented in Section 2. The proposed SOA approach is introduced and modeled in Section 3. Simulation studies and results are presented in Section 4. The effectiveness of SOA in handling real-world applications is challenged in Section 5. Conclusions and suggestions for future research are provided in Section 6.

## 2. Literature Review

Metaheuristic algorithms have been developed based on the simulation of various natural phenomena, natural behaviors of animals, birds, aquatic animals, insects, and other living creatures in the wild, physical laws and phenomena, biological sciences, genetics, human behaviors and interactions, rules of games, and other evolutionary phenomena. Therefore, based on the main idea used in the design, metaheuristic algorithms are classified into five groups: swarm-based, evolutionary-based, physics-based, game-based, and human-based.

Swarm-based algorithms are developed by being inspired by the swarming behavior of living organisms, such as, e.g., animals, birds, insects, and aquatics in nature. The most famous algorithms of this group can be mentioned: Particle Swarm Optimization (PSO) [13], Artificial Bee Colony (ABC) [14], and Ant Colony Optimization (ACO) [15]. PSO is developed based on the simulation of the movement of flocks of birds or fish that are searching for food. ABC is introduced inspired by the activities of a honey bee colony in obtaining food resources. ACO is designed based on modeling the ability of ants to find the optimal route between the nest and the food source. Searching for food resources and hunting strategy for providing food are natural behaviors among animals that are employed in the design of numerous metaheuristic algorithms such as the Coati Optimization Algorithm (COA) [16], Reptile Search Algorithm (RSA) [17], White Shark Optimizer (WSO) [18], Honey Badger Algorithm (HBA) [19], Golden Jackal Optimization (GJO) [20], African Vultures Optimization Algorithm (AVOA) [21], Grey Wolf Optimizer (GWO) [22], Whale Optimization Algorithm (WOA) [23], Marine Predator Algorithm (MPA) [24], and Tunicate Swarm Algorithm (TSA) [25].

Evolutionary-based algorithms are introduced with inspiration from biological and genetics sciences, random operators, concepts of natural selection, and survival of the fittest. Genetic Algorithm (GA) [26] and Differential Evolution (DE) [27] are among the most well-known and widely used metaheuristic algorithms that are designed based on reproduction simulation, Darwin’s theory of evolution, and stochastic operators such as selection, crossover, and mutation.

Physics-based algorithms are designed with inspiration from phenomena, concepts, and laws in physics. The Simulated Annealing (SA) [28] algorithm is one of the most famous physics-based approaches. The modeling of the metal annealing phenomenon in metallurgy has been the main idea in its design. Physical forces are the origin of the creation of algorithms such as the Spring Search Algorithm (SSA) [29] based on spring tensile force, the Gravitational Search Algorithm (GSA) [30] based on gravitational attraction force, and the Momentum Search Algorithm (MSA) [31] based on momentum force. The phenomenon of physical changes in water has been the main idea in Water Cycle Algorithm (WCA) design [32]. Concepts of cosmology have been the origin of Black Hole Algorithm (BHA) design [33]. Some of the most popular physics-based methods are: Equilibrium Optimizer (EO) [34], Electro-Magnetism Optimization (EMO) [35], Multi-Verse Optimizer (MVO) [36], Archimedes Optimization Algorithm (AOA) [37], Thermal Exchange Optimization (TEO) [38], and Lichtenberg Algorithm (LA) [39].

Game-based algorithms are developed with inspiration from various individual and group games, the behavior of players, coaches, referees, and other people influencing the game. Football Game Based Optimization (FGBO) [40] and Volleyball Premier League (VPL) [41] are two game-based approaches that are designed based on the modeling of holding league competitions. The common aspect of many games is the effort of players to earn points, which is the origin of the design of algorithms, including Darts Game Optimizer (DGO) [42], Puzzle Optimization Algorithm (POA) [43], Hide Object Game Optimizer (HOGO) [44], Archery Algorithm (AA) [8], and Tug of War Optimization (TWO) [45].

Human-based algorithms are introduced by taking inspiration from human behaviors, interactions, and thoughts. One of this group’s most widely used algorithms is Teaching-Learning Based Optimization (TLBO) [46], which is introduced based on the modeling of human behaviors between students and teachers in the classroom. Teammates’ efforts to achieve team goals have been the design idea of the Teamwork Optimization Algorithm (TOA) [47]. The therapeutic activities of doctors in treating patients have inspired the design of Doctor and Patient Optimization (DPO) [48]. Some of the other popular human-based methods are: Ali Baba and the Forty Thieves (AFT) [49], Coronavirus Herd Immunity Optimizer (CHIO) [50], War Strategy Optimization (WSO) [51], and Gaining Sharing Knowledge based Algorithm (GSK) [52].

Based on the best knowledge obtained from the literature review, no metaheuristic algorithm has been designed so far based on the simulation of natural behaviors of servals. At the same time, the serval’s strategy during hunting and capturing prey is an intelligent process with the potential to design an optimizer. In order to address this research gap, in this paper, the natural behavior of servals during hunting in nature is employed in the design of a new bio-inspired metaheuristic algorithm, which is introduced and modeled in the next section.

## 3. Serval Optimization Algorithm

This section is dedicated to the introduction and mathematical modeling of the proposed Serval Optimization Algorithm (SOA) approach.

### 3.1. Inspiration of SOA

Serval is a skilled predator that hunts its prey in three stages. First, using its strong sense of hearing, it identifies the position of the prey and observes it for up to 15 min without moving. Then, in the second stage, it moves towards the prey, jumps up to a height of 4 meters in the air with all four feet, and attacks this prey with its front paws. Finally, in the third stage, in a chasing process by running and jumping to catch the fleeing prey, the serval kills it and starts eating it [53]. 

Serval’s strategy during hunting is one of the most characteristic natural behaviors of this animal. This strategy is an intelligent process that can inspire the design of a new metaheuristic algorithm. Modeling the three-stage serval strategy during hunting is employed in SOA design, which is discussed below.

### 3.2. Algorithm Initialization

The proposed SOA approach is a population-based optimizer that is able to provide suitable solutions for optimization problems by using the search power of its search agents. Servals that look for prey in nature have a similar approach to the mechanism of search agents in identifying the optimal solution. For this reason, from a mathematical point of view, servals form the SOA population that seeks to achieve the optimal solution in the search space. Therefore, each serval is a candidate solution for the problem whose position in the search space determines the values of the decision variables. From a mathematical point of view, each serval is a vector, and their population together forms the SOA population matrix, which can be represented according to Equation (1). The initial position of servals in the search space at the beginning of the implementation of the algorithm is randomly generated using Equation (2).

(1) $$X={\left[\begin{array}{c} X_{1}\\ \vdots \\ X_{i}\\ \vdots \\ X_{N} \end{array}\right]}_{N\times d}={\left[\begin{array}{ccccc} x_{1,1} & \cdots & x_{1,j} & \cdots & x_{1,d}\\ \vdots & \ddots & \vdots & ⋰ & \vdots \\ x_{i,1} & \cdots & x_{i,j} & \cdots & x_{i,d}\\ \vdots & ⋰ & \vdots & \ddots & \vdots \\ x_{N,1} & \cdots & x_{N,j} & \cdots & x_{N,d} \end{array}\right]}_{N\times d},$$

(2) $$x_{i,j}=lb_{j}+r_{i,j}\cdot \left(ub_{j}-lb_{j}\right),\;i=1,2,\;\ldots ,\;N\;\mathrm{and}\;j=1,2,\;\ldots ,d,$$

where X denotes the population matrix of serval locations, $X_{i}$ is the ith serval (candidate solution), $x_{i,j}$ is its jth dimension in search space (decision variable), N denotes the number of servals, d is the number of decision variables, $r_{i,j}$ are random numbers in the interval [0,1], $lb_{j}$, and $ub_{j}$ are the lower and upper bounds of the jth decision variable, respectively.

Since each serval is a candidate solution for the problem, the objective function of the problem can be evaluated based on the proposed values of each serval for the decision variables. Then, according to Equation (3), a vector can represent the values of the problem’s objective function.

(3) $$F={\left[\begin{array}{c} F_{1}\\ \vdots \\ F_{i}\\ \vdots \\ F_{N} \end{array}\right]}_{N\times 1}={\left[\begin{array}{c} F\left(X_{1}\right)\\ \vdots \\ F\left(X_{i}\right)\\ \vdots \\ F\left(X_{N}\right) \end{array}\right]}_{N\times 1},$$

where F denotes the vector of objective function values and $F_{i}$ denote to the obtained objective function value from the ith serval.

Among the calculated values for the objective function, the best value is identified as the best candidate solution, and the member corresponding to it is determined as the best member of the population. Considering that in each SOA iteration, the positions of all population members are updated, of course, the best member should be updated in each iteration.

### 3.3. Mathematical Modelling of SOA

The process of updating SOA population members in the search space has two phases based on simulating the serval hunting strategy in nature. These phases are intended to model exploration in global search and exploitation in local search in SOA design.

#### 3.3.1. Phase 1: Prey Selection and Attacking (Exploration)

The serval is an efficient predator that uses its strong sense of hearing to identify the location of its prey and then attack it. In the first phase of SOA, the positions of servals are updated based on the simulation of these two strategies. This update causes big changes in the position of servals and leads to a detailed scanning of the search space. The purpose of this phase of SOA is to increase the power of SOA exploration in global search and to identify the main optimal region.

In the SOA design, the position of the population’s best member is considered the prey position. First, the new position for the serval is calculated using Equation (4) to model the serval’s attack on the prey. Then, if this new position improves the value of the objective function, it replaces the previous serval position according to Equation (5).

(4) $$x_{i,j}^{P1}=x_{i,j}+r_{i,j}\cdot \left(P_{j}-I_{i,j}\cdot x_{i,j}\right),\;i=1,2,\;\ldots ,\;N\;\mathrm{and}\;j=1,2,\;\ldots ,d,$$

(5) $$X_{i}=\{\begin{array}{c} X_{i}^{P1},\;F_{i}^{P1}<F_{i}\\ X_{i},\;else \end{array}$$

where $X_{i}^{P1}$ denotes the new position of the ith serval based on the first phase of SOA, $x_{i,j}^{P1}$ is its jth dimension, $F_{i}^{P1}$ is its objective function value, $r_{i,j}$ are random numbers in interval [0,1], P denotes the prey location, $P_{j}$ is its jth dimension, $I_{i,j}$ are numbers randomly selected from the set {1,2}, N is the total number of servals population, and d is the number of decision variables.

#### 3.3.2. Phase 2: Chase Process (Exploitation)

After attacking the prey, the serval tries to stop the prey by leaping in a chase process, then kills it and feeds on it. In the second phase of SOA, this serval strategy is employed in updating the population position of SOA. The simulation of the chase process causes small changes in the positions of the servals in the search space. In fact, the purpose of this SOA phase is to increase the exploitation power of SOA in local search and find better solutions. In order to mathematically model the chasing process between the serval and the prey, a new random position near the serval is calculated using Equation (6). This new position, provided that it improves the value of the objective function, replaces the previous position of the corresponding serval according to Equation (7).

(6) $$x_{i,j}^{P2}=x_{i,j}+\frac{r_{i,j}\cdot \left(ub_{j}-lb_{j}\right)}{t},\;i=1,2,\;\ldots ,\;N,\;j=1,2,\;\ldots ,d,\;\mathrm{and}\;t=1,2,\;\ldots ,\;T,$$

(7) $$X_{i}=\{\begin{array}{c} X_{i}^{P2},\;F_{i}^{P2}<F_{i},\\ X_{i},\;else, \end{array}$$

where $X_{i}^{P2}$ represents the new position of the ith serval based on second phase of SOA, $x_{i,j}^{P2}$ is its jth dimension, $F_{i}^{P2}$ denotes its objective function value, t is the iteration counter of the algorithm, and T represents to the total number of algorithm iterations.

### 3.4. Repetition Process, Pseudocode, and Flowchart of SOA

**Figure 1:**
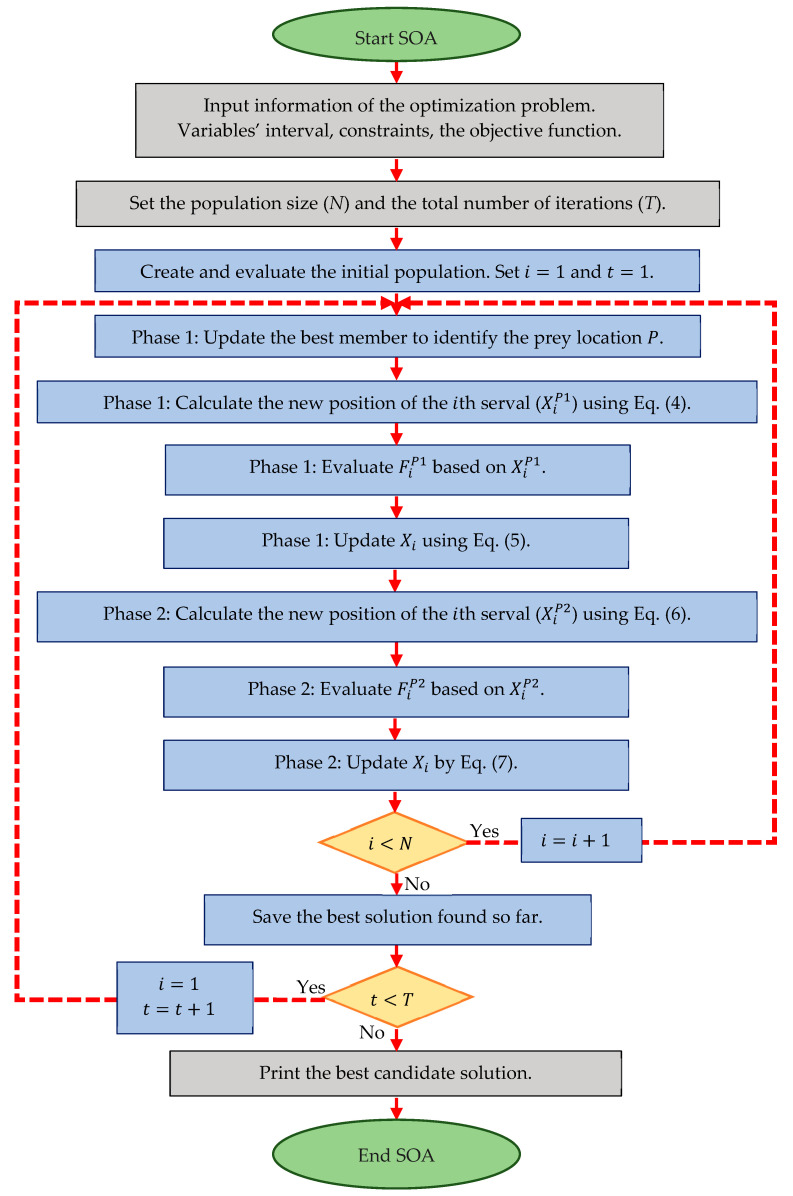
Flowchart of the proposed SOA.

By updating all servals based on the first and second phases of SOA, the first iteration of the algorithm is completed. Then, based on the new positions of the servals and the new values obtained for the objective function, the algorithm enters the next iteration. The operation of updating the positions of servals is repeated until the last iteration of the algorithm based on Equations (4)–(7). After the complete implementation of SOA, the best candidate solution obtained during the algorithm’s execution is introduced as the solution to the problem. The SOA implementation process is presented in the form of a flowchart in Figure 1, and its pseudo code is presented in Algorithm 1.

**Algorithm 1** Pseudocode of the SOA.Start SOA.1. Input problem information: variables, the objective function, and constraints.2. Set the population size (*N*) and the total number of iterations (*T*)3. Generate the initial population matrix at random.4. Evaluate the objective function.5. For *t* = 1 to *N*6.   For *i* = 1 to *N*7.   Phase 1: Prey selection and attacking (exploration)8.     Update the best member of population as prey location.9.     Calculate the new position of the *i*th SOA member based on attack simulation using Equation (4). $x_{i,j}^{P1}\leftarrow x_{i,j}+r_{i,j}\cdot \left(P_{j}-I_{i,j}\cdot x_{i,j}\right)$10.     Update the ith SOA member using Equation (5).
$X_{i}\leftarrow \{\begin{array}{c} X_{i}^{P1},\;F_{i}^{P1}<F_{i},\\ X_{i},\;\mathrm{else}, \end{array}$11.   Phase 2: Chase process (exploitation)12.     Calculate new position of the ith SOA member based on simulation the chase using Equation (6).
$x_{i,j}^{P2}\leftarrow x_{i,j}+\frac{r_{i,j}\cdot \left(ub_{j}-lb_{j}\right)}{t}$
13.     Update the ith SOA member using Equation (7). $X_{i}\leftarrow \{\begin{array}{c} X_{i}^{P2},\;F_{i}^{P2}<F_{i},\\ X_{i},\;\mathrm{else}, \end{array}$
14.   end15.   Save the best candidate solution so far.16. end.17. Output the best quasi-optimal solution obtained with the SOA.End SOA.

### 3.5. Computational Complexity of SOA

This subsection is dedicated to the computational complexity analysis of the proposed SOA approach. SOA initialization operation has a complexity equal to O(Nd), where *N* is the number of servals and *d* is the number of decision variables. The process of updating the SOA population and calculating the objective function in two phases has a complexity equal to O(2NdT), where *T* is the maximum number of algorithm iterations. Therefore, the total computational complexity of SOA is O(Nd(1+2T)).

## 4. Simulation Studies and Results

This section is dedicated to evaluating the performance of SOA in solving optimization problems and achieving solutions for these problems. For this purpose, thirty-nine standard benchmark functions from the CEC 2017 test suite and CEC 2019 test suite have been employed. The CEC 2017 test suite has 30 benchmark functions, including 3 unimodal functions C17-F1 to C17-F3, 7 multimodal functions C17-F4 to C17-F10, 10 hybrid functions C17-F11 to C17-F20, and 10 composition functions C17-F21 to C17-F30. The C17-F2 function is not considered in the simulations due to its unstable behavior. The complete information of the CEC 2017 test suite is described in [54]. CEC 2019 test suite has 10 hard benchmark functions, the complete information of which is described in [55]. The quality of the SOA approach in optimization has been compared with the performance of twelve well-known metaheuristic algorithms. These algorithms include: (i) widely used and famous methods: GA, PSO, (ii) high cited methods: GSA, TLBO, MVO, GWO, WOA (iii) recently published methods MPA, TSA, RSA, AVOA, and WSO. The adjusted values for the parameters of competitor algorithms are listed in Table 1.

SOA and competitor algorithms are employed to optimize the 39 benchmark functions mentioned above. Simulation results are presented using six indicators: mean, best, worst, standard deviation (std), median, and rank.

### 4.1. Evaluation the CEC 2017 Test Suite

To analyze the quality of SOA and competitor algorithms in handling optimization problems, they have been implemented on the CEC 2017 test suite for dimensions d equal to 10, 30, 50, and 100. Table 2, Table 3, Table 4 and Table 5 present the results obtained from these implementations.

What can be concluded from the comparison of simulation results is that for the dimension d=10, SOA is the best optimizer in handling the functions C17-F3, C17-F6, C17-F7, C17-F10, C17-F19 to C17-F24, C17-F26 to C17-F30. For the dimension d=30, SOA is the best optimizer in handling the functions C17-F1, C17-F3 to C17-F5, C17-F7 to C17-F11, C17-F14 to C17-F16, C17-F20, C17-F21, C17-F23, C17-F26, C17-F27, C17-F29, and C17-F30. For the dimension d=50, SOA is the best optimizer in handling the functions C17-F1, C17-F3 to C17-F10, C17-F12 to C17-F20, C17-F22, C17-F23, and C17-F25, to C17-F30. For the dimension d=100, SOA is the best optimizer in handling the functions C17-F1, C17-F3 to C17-F5, C17-F7 to C17-F13, C17-F15, C17-F16, C17-F18, to C17-F22, C17-F24, to C17-F26, C17-F29, and C17-F30.

**Figure 2:**
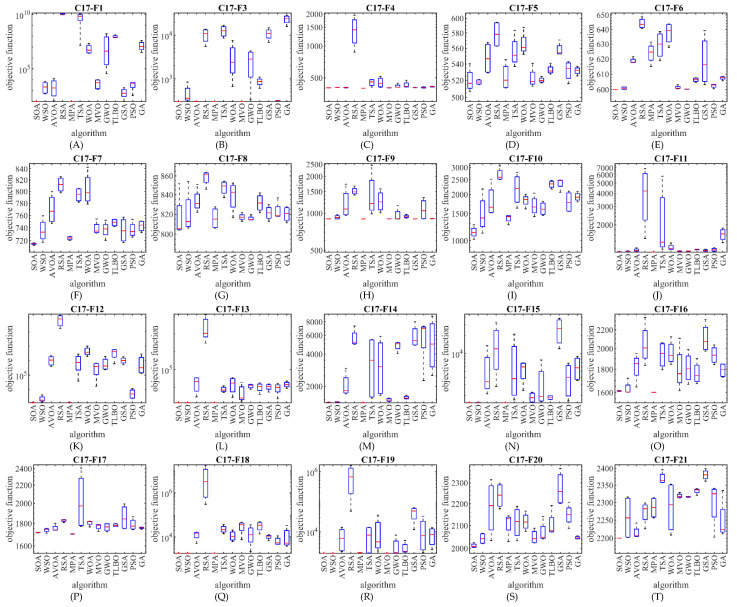

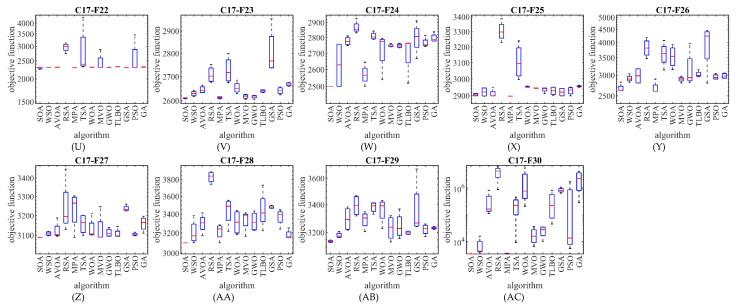
Boxplot diagram of SOA and competitor algorithms performances on the CEC 2017 test suite (for the dimension d=10).

**Figure 3:**
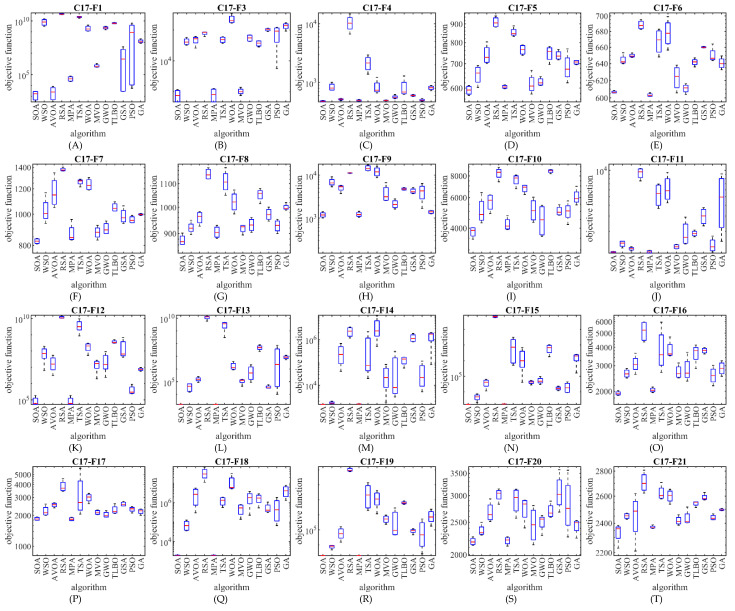

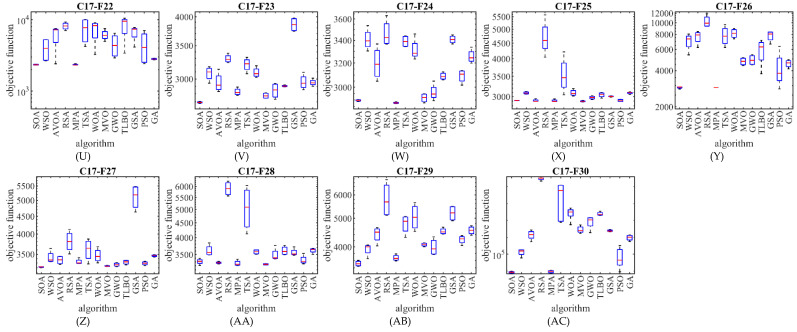
Boxplot diagram of SOA and competitor algorithms performances on the CEC 2017 test suite (for the dimension d=30).

**Figure 4:**
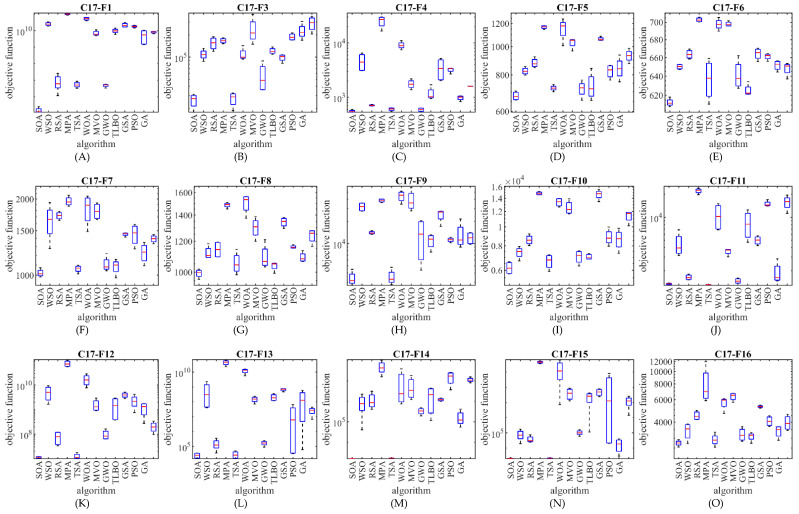

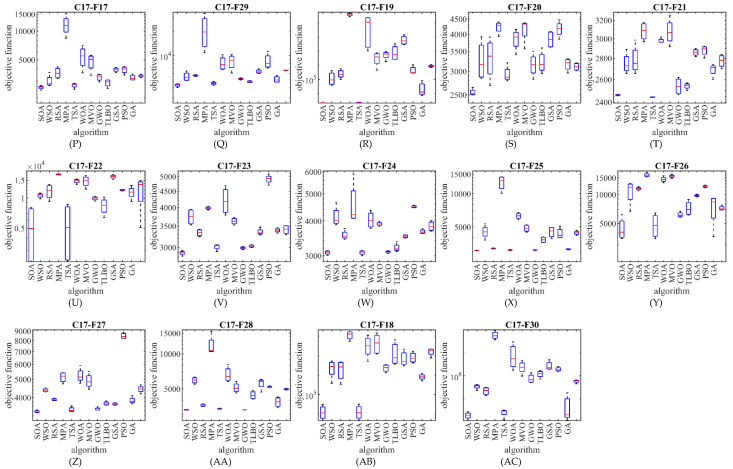
Boxplot diagram of SOA and competitor algorithms performances on the CEC 2017 test suite (for the dimension d=50).

**Figure 5:**
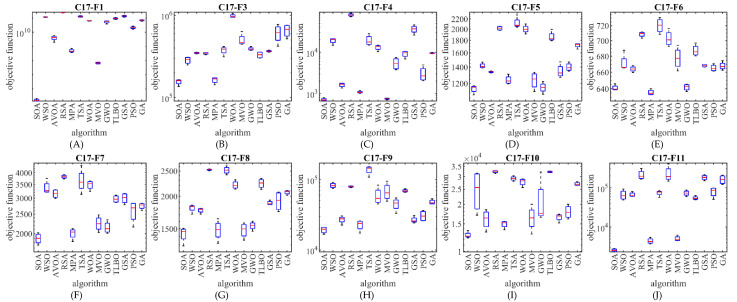

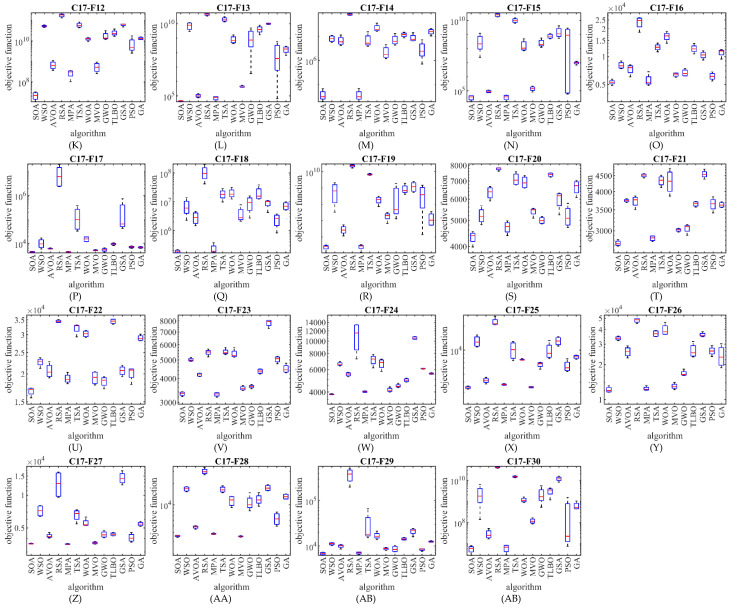
Boxplot diagram of SOA and competitor algorithms performances on the CEC 2017 test suite (for the dimension d=100).

The optimization results show that the proposed SOA approach has provided superior performance compared to competing algorithms in the CEC 2017 test suite optimization by creating a suitable balance between exploration and exploitation. The performance of SOA and competitor algorithms in the optimization of the CEC 2017 test suite is drawn as a boxplot diagram in Figure 2, Figure 3, Figure 4 and Figure 5.

### 4.2. Evaluation the CEC 2019 Test Suite

This subsection tests the effectiveness of the proposed SOA approach and competing algorithms in solving the CEC 2019 test suite. The optimization results of C19-F1 to C19-F10 functions are published in Table 6.

**Figure 6:**
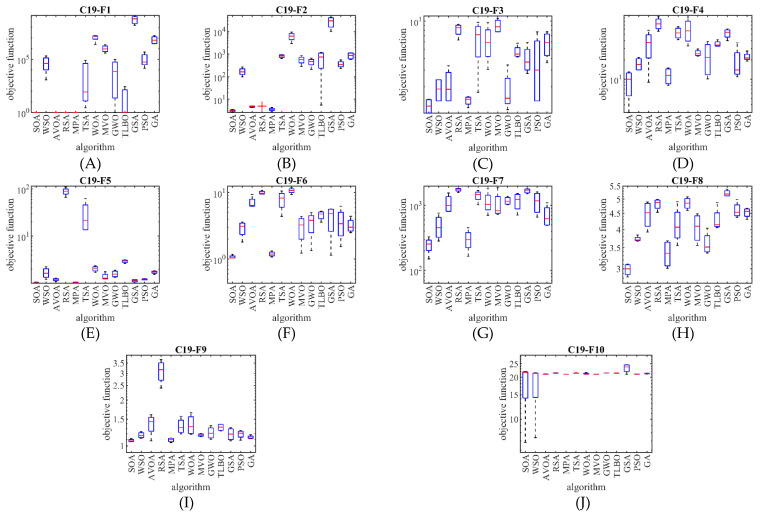
Boxplot diagram of SOA and competitor algorithms performances on the CEC 2019 test suite.

What is evident from the comparison of the simulation results is that the proposed SOA approach is the first best optimizer C19-F1 to C19-F4, and C19-F6 to C19-F9 functions against competitor algorithms. The optimization results show that SOA’s proposed approach better handles the CEC 2019 test suite by winning the first rank compared to competitor algorithms. The performance of SOA and competitor algorithms in the optimization of the CEC 2019 test suite is drawn as a boxplot diagram in Figure 6.

### 4.3. Statistical Analysis

In this subsection, by providing a statistical analysis of the simulation results, it has been investigated how significant the superiority of the proposed SOA approach is against competitor algorithms from a statistical point of view. For this reason, the Wilcoxon rank sum test [56] is utilized, which is applicable to determine the significant difference between the average of two data samples. The results of applying the Wilcoxon rank sum test on the performance of the proposed SOA proposed approach and competitor algorithms are reported in Table 7. Based on the values obtained for the p-value index, in cases where the *p*-value is less than 0.05, the proposed SOA approach has a statistically significant superiority compared to the corresponding competitor algorithm.

## 5. SOA for Real-World Applications

This section is dedicated to analyzing the effectiveness of the proposed SOA approach in handling real-world applications. In this regard, SOA and competitor algorithms are employed to optimize the CEC 2011 test suite and four engineering design problems.

### 5.1. Evaluation the CEC 2011 Test Suite

This collection contains twenty-two real-world optimization problems (the C11-F3 function was excluded in the simulation studies). The CEC 2011 test suite details are described in [57]. The optimization results of the CEC 2011 test suite using SOA and competitor algorithms are published in Table 8.

**Figure 7:**
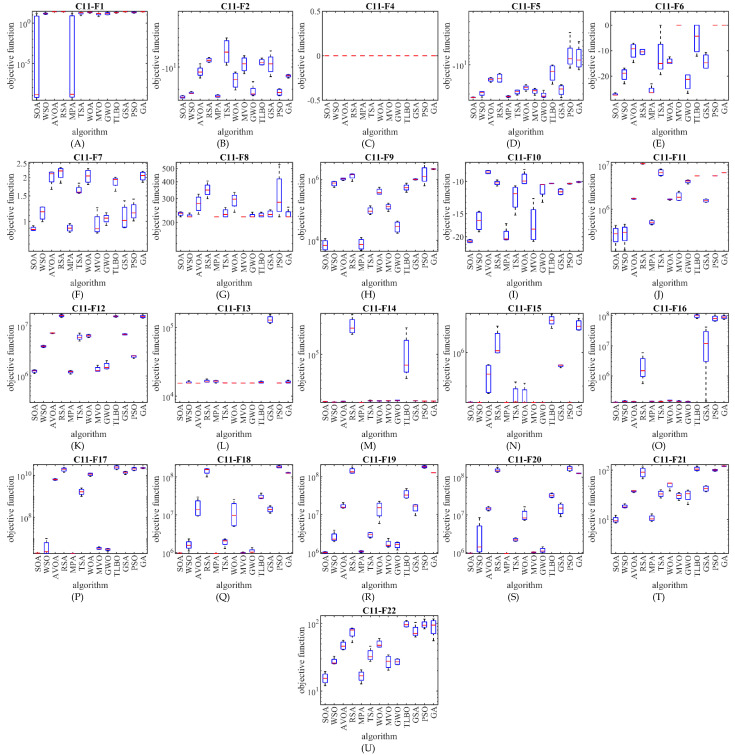
Boxplot diagram of SOA and competitor algorithms performances on the CEC 2011 test suite.

The simulation results imply that the proposed SOA approach is the best optimizer for handling functions C11-F1, C11-F2, C11-F4 to C11-F6, C11-F8 to C11-F10, F12, F15, F18, and C11-F20 to C11-F22. A comparison of the simulation results indicates that the proposed SOA approach has an acceptable efficiency in dealing with real-world optimization problems against competitor algorithms. Additionally, the results of employing the Wilcoxon rank sum test on the performance of SOA and competitor algorithms on the CEC 2011 test suite show the statistically significant superiority of SOA in competition with the compared algorithms. The performance of SOA and competitor algorithms in dealing with the CEC 2011 test suite is plotted as a boxplot diagram in Figure 7.

### 5.2. The SOA Testing on Engineering Optimization Problems

In this subsection, the performance of SOA in solving four engineering design problems from real-world applications is evaluated.

#### 5.2.1. Pressure Vessel Design Problem

**Figure 8:**
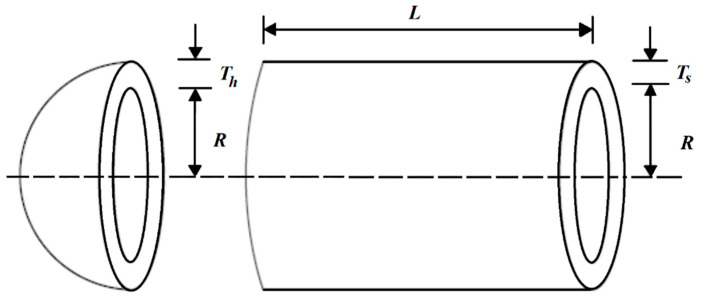
Schematic of the pressure vessel design.

The pressure vessel design is a real-world challenge in engineering studies where the goal is to minimize the design cost. The schematic of this design is provided in Figure 8. 

The mathematical model of pressure vessel design problem is as follows [58]:

Consider: $X=\left[x_{1},\;x_{2},\;x_{3},\;x_{4}\right]=\left[T_{s},\;T_{h},\;R,\;L\right]$.

Minimize: $f\left(x\right)=0.6224x_{1}x_{3}x_{4}+1.778x_{2}x_{3}^{2}+3.1661x_{1}^{2}x_{4}+19.84x_{1}^{2}x_{3}$.

Subject to:

$g_{1}\left(x\right)=-x_{1}+0.0193x_{3}\;\leq \;0,\;g_{2}\left(x\right)=-x_{2}+0.00954x_{3}\leq \;0,\;g_{3}\left(x\right)=-\pi x_{3}^{2}x_{4}-\frac{4}{3}\pi x_{3}^{3}+1296000\leq \;0,\;g_{4}\left(x\right)=x_{4}-240\;\leq \;0.$

With

$0\leq x_{1},x_{2}\leq 100\;\mathrm{and}\;10\leq x_{3},x_{4}\leq 200$.

The optimization results of pressure vessel design using SOA and competitor algorithms are released in Table 9 and Table 10. 

**Figure 9:**
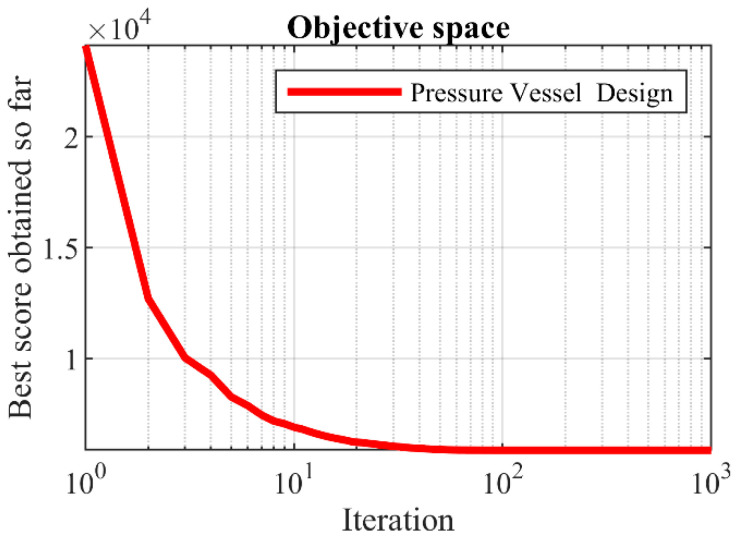
SOA’s performance convergence curve on the pressure vessel design.

Based on the simulation results, the proposed SOA approach has provided the optimal solution with the values of the design variables equal to (0.778027, 0.384579, 40.31228, 200), and the value of the objective function equals to 5882.901. The analysis of the results shows that compared to competitor algorithms. Therefore, SOA has provided better performance in dealing with pressure vessel design. The convergence curve of SOA in achieving the solution for the pressure vessel design problem is drawn in Figure 9.

#### 5.2.2. Speed Reducer Design Problem

**Figure 10:**
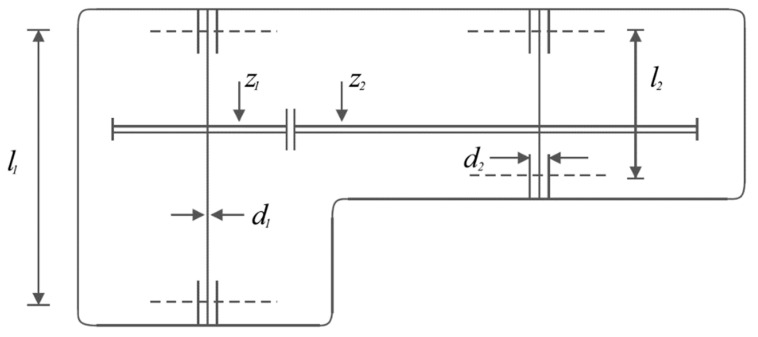
Schematic of speed reducer design.

The speed reducer design is an engineering subject aiming to minimize the speed reducer’s weight. The schematic of this design is provided in Figure 10. 

The mathematical model of the speed reducer design problem is as follows [59,60]:

Consider: $X=\left[x_{1,}\;x_{2},\;x_{3},\;x_{4},\;x_{5}\;,x_{6}\;,x_{7}\right]=\left[b,\;m,\;p,\;l_{1},\;l_{2},\;d_{1},\;d_{2}\right]$.

Minimize: $f\left(x\right)=0.7854x_{1}x_{2}^{2}\left(3.3333x_{3}^{2}+14.9334x_{3}-43.0934\right)-1.508x_{1}\left(x_{6}^{2}+x_{7}^{2}\right)+7.4777\left(x_{6}^{3}+x_{7}^{3}\right)+0.7854(x_{4}x_{6}^{2}+x_{5}x_{7}^{2}).$

Subject to:

$g_{1}\left(x\right)=\frac{27}{x_{1}x_{2}^{2}x_{3}}-1\;\leq \;0,\;g_{2}\left(x\right)=\frac{397.5}{x_{1}x_{2}^{2}x_{3}}-1\leq \;0,\;g_{3}\left(x\right)=\frac{1.93x_{4}^{3}}{x_{2}x_{3}x_{6}^{4}}-1\leq \;0,\;g_{4}\left(x\right)=\frac{1.93x_{5}^{3}}{x_{2}x_{3}x_{7}^{4}}-1\;\leq \;0,\;g_{5}\left(x\right)=\frac{1}{110x_{6}^{3}}\sqrt{{\left(\frac{745x_{4}}{x_{2}x_{3}}\right)}^{2}+16.9\cdot 10^{6}}-1\leq \;0,\;g_{6}\left(x\right)=\frac{1}{85x_{7}^{3}}\sqrt{{\left(\frac{745x_{5}}{x_{2}x_{3}}\right)}^{2}+157.5\cdot 10^{6}}-1\;\leq \;0,\;g_{7}\left(x\right)=\frac{x_{2}x_{3}}{40}-1\;\leq \;0,\;g_{8}\left(x\right)=\frac{5x_{2}}{x_{1}}-1\;\leq \;0,\;g_{9}\left(x\right)=\frac{x_{1}}{12x_{2}}-1\;\leq \;0,\;g_{10}\left(x\right)=\frac{1.5x_{6}+1.9}{x_{4}}-1\;\leq \;0,\;g_{11}\left(x\right)=\frac{1.1x_{7}+1.9}{x_{5}}-1\;\leq \;0.$

With 

$2.6\leq x_{1}\leq 3.6,\;0.7\leq x_{2}\leq 0.8,\;17\leq x_{3}\leq 28,\;7.3\leq x_{4}\leq 8.3,\;7.8\leq x_{5}\leq 8.3,\;2.9\leq x_{6}\leq 3.9,\;\mathrm{and}\;5\leq x_{7}\leq 5.5.$

The implementation results of the proposed SOA and competitor algorithms on the speed reducer design problem are released in Table 11 and Table 12.

**Figure 11:**
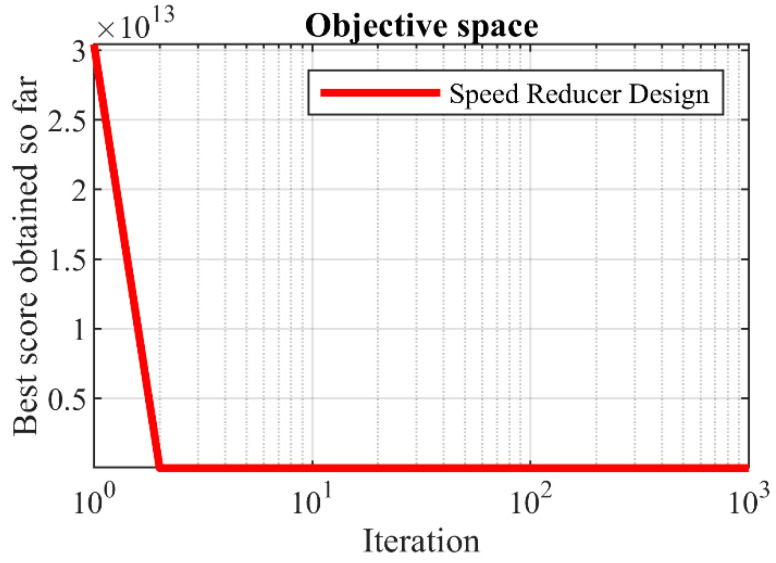
SOA’s performance convergence curve on the speed reducer design.

Based on the simulation results, the proposed SOA approach has provided the optimal solution with the values of the design variables equal to (3.5, 0.7, 17, 7.3, 7.8, 3.350215, 5.286683) and the objective function equal to 2996.348. Analysis of the results shows that SOA has provided better performance in handling speed reducer design compared to competitor algorithms. The convergence curve of SOA while solving the speed reducer design problem is drawn in Figure 11.

#### 5.2.3. Welded Beam Design

**Figure 12:**
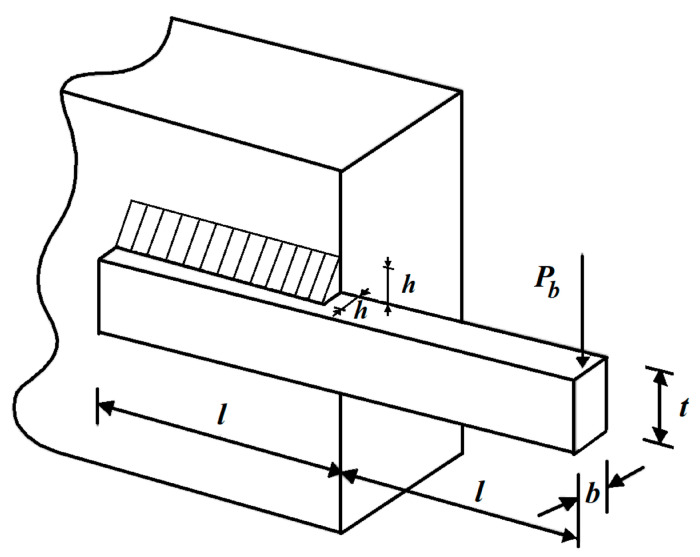
Schematic of the welded beam design.

The welded beam design is a real-world application with the aim of minimizing the fabrication cost of the welded beam. The schematic of welded beam design problem is provided in Figure 12. 

The mathematical model of welded beam design problem is as follows [23]:

Consider: $X=\left[x_{1},\;x_{2},\;x_{3},\;x_{4}\right]=\left[h,\;l,\;t,\;b\right]$.

Minimize: $f\left(x\right)=1.10471x_{1}^{2}x_{2}+0.04811x_{3}x_{4}\;\left(14.0+x_{2}\right)$.

Subject to:

$g_{1}\left(x\right)=\tau \left(x\right)-13600\;\leq \;0,\;g_{2}\left(x\right)=\sigma \left(x\right)-30000\;\leq \;0,\;g_{3}\left(x\right)=x_{1}-x_{4}\leq \;0,\;g_{4}\left(x\right)=0.10471x_{1}^{2}+0.04811x_{3}x_{4}\;\left(14+x_{2}\right)-5.0\;\leq \;0,\;g_{5}\left(x\right)=0.125-x_{1}\leq \;0,\;g_{6}\left(x\right)=\delta \;\left(x\right)-0.25\;\leq \;0,\;g_{7}\left(x\right)=6000-p_{c}\;\left(x\right)\leq \;0.$

Here

$\tau \left(x\right)=\sqrt{{\left(\tau^{\prime }\right)}^{2}+\left(2\tau \tau^{\prime }\right)\frac{x_{2}}{2R}+{\left(\tau ”\right)}^{2}\;},\;\tau^{\prime }=\frac{6000}{\sqrt{2}x_{1}x_{2}},\;\tau ”=\frac{MR}{J},\;M=6000\left(14+\frac{x_{2}}{2}\right),\;R=\sqrt{\frac{x_{2}^{2}}{4}+{\left(\frac{x_{1}+x_{3}}{2}\right)}^{2}},\;J=2\left\{x_{1}x_{2}\sqrt{2}\left[\frac{x_{2}^{2}}{12}+{\left(\frac{x_{1}+x_{3}}{2}\right)}^{2}\right]\right\}\;,\;\sigma \left(x\right)=\frac{504000}{x_{4}x_{3}^{2}},$

$\delta \;\left(x\right)=\frac{65856000}{\left(30\cdot 10^{6}\right)x_{4}x_{3}^{3}}\;,\;p_{c}\;\left(x\right)=\frac{4.013\left(30\cdot 10^{6}\right)\sqrt{\frac{x_{3}^{2}x_{4}^{6}}{36}}}{196}\left(1-\frac{x_{3}}{28}\sqrt{\frac{30\cdot 10^{6}}{4\left(12\cdot 10^{6}\right)}}\right).$

With

$0.1\leq x_{1},\;x_{4}\leq 2\;\mathrm{and}\;0.1\leq x_{2},\;x_{3}\leq 10$.

The results of using the SOA and competing algorithms on the problem of welded beam design are released in Table 13 and Table 14. 

**Figure 13:**
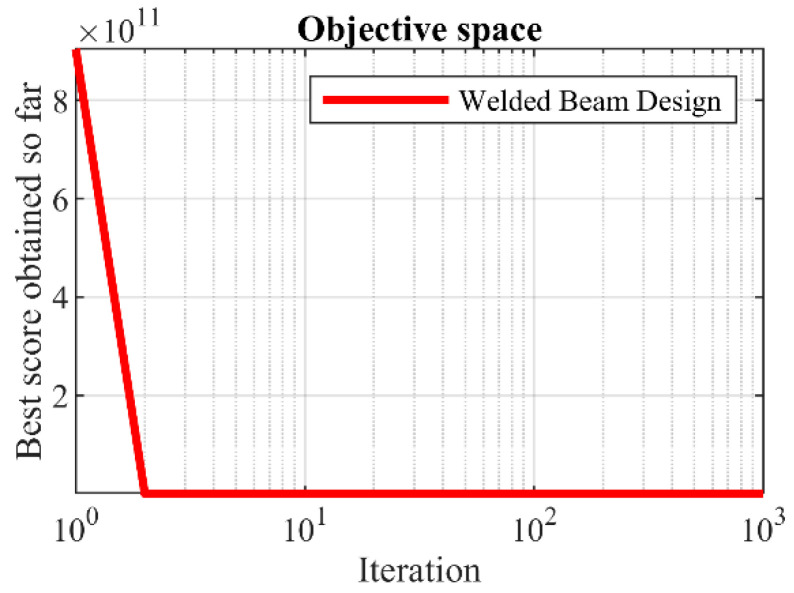
SOA’s performance convergence curve on the welded beam design.

Based on the simulation results, the proposed SOA approach has provided the optimal solution with the values of the design variables equal to (0.20573, 3.470489, 9.036624, 0.20573) and the objective function equal to 1.724852. Based on the statistical indicators, it is clear that SOA has provided a more effective capability in handling the welded beam design problem compared to competitor algorithms. The SOA con-vergence curve during welded beam design optimization is drawn in Figure 13.

#### 5.2.4. Tension/Compression Spring Design

**Figure 14:**
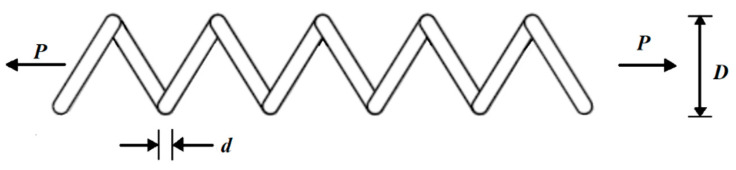
Schematic of the tension/compression spring design.

The tension/compression spring design is a real-world issue with the goal of minimizing the weight of tension/compression spring. The schematic of this design is provided in Figure 14. 

The mathematical model of tension/compression spring design problem is as follows [23]:

Consider: $X=\left[x_{1},\;x_{2},\;x_{3}\;\right]=\left[d,\;D,\;P\right].$

Minimize: $f\left(x\right)=\left(x_{3}+2\right)x_{2}x_{1}^{2}.$

Subject to:

$g_{1}\left(x\right)=1-\frac{x_{2}^{3}x_{3}}{71785x_{1}^{4}}\;\leq \;0,\;g_{2}\left(x\right)=\frac{4x_{2}^{2}-x_{1}x_{2}}{12566\left(x_{2}x_{1}^{3}\right)}+\frac{1}{5108x_{1}^{2}}-1\leq \;0,$

$g_{3}\left(x\right)=1-\frac{140.45x_{1}}{x_{2}^{2}x_{3}}\leq \;0,\;g_{4}\left(x\right)=\frac{x_{1}+x_{2}}{1.5}-1\;\leq \;0$.

With 

$0.05\leq x_{1}\leq 2,\;0.25\leq x_{2}\leq 1.3\;\mathrm{and}\;2\leq \;x_{3}\leq 15$.

The simulation results of the tension/compression spring design problem using the SOA and competitor algorithms are released in Table 15 and Table 16. 

**Figure 15:**
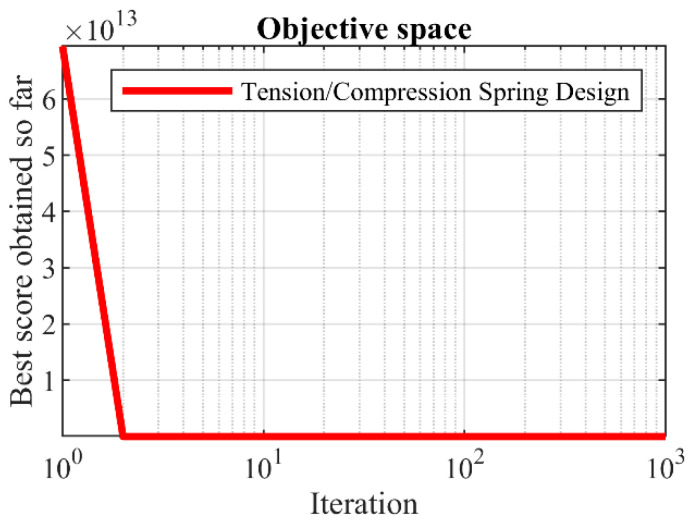
SOA’s performance convergence curve on the tension/compression spring.

Based on the simulation results, the proposed SOA approach has provided the optimal solution with the values of the design variables equal to (0.051689, 0.356718, 11.28897) and the objective function equal to 0.012665. Comparing the obtained results indicates the superiority of SOA in dealing with the tension/compression spring design problem compared to competing algorithms. The SOA convergence curve while achieving the optimal design for the tension/compression spring design problem is drawn in Figure 15.

## 6. Conclusions and Future Works

This paper introduced a new swarm-based metaheuristic algorithm named the Serval Optimization Algorithm (SOA) based on the simulation of serval behaviors in nature. The serval strategy during hunting in the three stages of prey selection, attack, and the chase is the fundamental inspiration of SOA. Different steps of SOA were stated and mathematically modeled in two phases of exploration and exploitation. The effectiveness of SOA in solving optimization problems was tested on thirty-nine benchmark functions from the CEC 2017 test suite and the CEC 2019 test suite. The SOA’s results were compared with the performance of the other twelve well-known metaheuristic algorithms. The optimization results showed that SOA had performed better by balancing exploration and exploitation and had superior performance compared to competitor algorithms. Employing the proposed approach in optimizing the CEC 2011 test suite and four engineering design challenges demonstrated SOA’s evident ability to address real-world applications.

The introduction of SOA enables several research tasks for future studies. Designing the multi-objective version of SOA and using it in multi-objective optimization problems, developing the binary version of SOA, and using it in applications that require binary algorithms, such as feature selection, are among the most special suggestions for future studies. The use of SOA in various optimization problems in science and real-world applications are among the other recommendations of this article for future research.

## Figures and Tables

**Table 1:** Control parameters values.

Algorithm	Parameter	Value
GA		
	Type	Real coded
	Selection	Roulette wheel (Proportionate)
	Crossover	Whole arithmetic (Probability=0.8, α∈[−0.5, 1.5])
	Mutation	Gaussian (Probability=0.05)
PSO		
	Topology	Fully connected
	Cognitive and social constant	$\left(C_{1},C_{2}\right)=\left(2,\;2\right)$
	Inertia weight	Linear reduction from 0.9 to 0.1
	Velocity limit	10% of dimension range
GSA		
	Alpha, $G_{0}$, $R_{norm}$, $R_{power}$	20, 100, 2, 1
TLBO		
	$T_{F}:$ teaching factor	$T_{F}=\;\mathrm{round}\;\;\left[\left(1+rand\right)\right]$
	random number	*rand* is a random number between 0 and 1.
GWO		
	Convergence parameter (*a*)	*a*: Linear reduction from 2 to 0.
MVO		
	wormhole existence probability (WEP)	Min(WEP)=0.2 and Max(WEP)=1.
	Exploitation accuracy over the iterations (*p*)	p=6.
WOA		
	Convergence parameter (*a*)	*a*: Linear reduction from 2 to 0.
	*r* is a random vector in [0, 1].	
	*l* is a random number from [−1, 1].	
TSA		
	$P_{min}\;\mathrm{and}\;P_{max}\;$	1, 4
	$c_{1},c_{2},c_{3}$	random numbers lie in the interval [0, 1].
MPA		
	Constant number	P=0.5
	Random vector	R is a vector of uniform random numbers in [0, 1].
	Fish Aggregating Devices (*FADs*)	FADs=0.2
	Binary vector	U=0 or 1
RSA		
	Sensitive parameter	β=0.01
	Sensitive parameter	α=0.1
	Evolutionary Sense (ES)	ES: randomly decreasing values between 2 and −2
AVOA		
	$L,L_{2}$	0.8, 0.2
	w	2.5
	$P_{1},P_{2},P_{3}$	0.6, 0.4, 0.6
WSO		
	$F_{min}\;\mathrm{and}\;F_{max}$	0.07, 0.75
	$\tau ,a_{0},a_{1},a_{2}$	4.125, 6.25, 100, 0.0005

**Table 2:** Optimization results of the CEC 2017 test suite (for the dimension d=10).

		SOA	WSO	AVOA	RSA	MPA	TSA	WOA	MVO	GWO	TLBO	GSA	PSO	GA
C17-F1	mean	100.827	3072.047	4092.864	1.09 × 10^10^	100	6.28 × 10^9^	8734,850	5565.472	43,130,985	92,363,844	789.6176	3796.408	16,141,320
	best	100.5137	501.1341	116.6581	9.41 × 10^9^	100	14,208,120	2734,095	1329.943	36,727.39	75,427,798	100.0205	352.658	3,264,472
	worst	100.9824	7395.231	12,699.46	1.3 × 10^10^	100	1.15 × 10^10^	21,710,549	10,084.16	1.64 × 10^8^	1.31 × 10^8^	1902.646	5631.975	38,820,667
	std	0.214676	3233.023	5877.613	1.61 × 10^9^	5.28 × 10^−6^	4.84× 10^9^	8,902,280	4689.867	80,613,752	26,120,139	779.9287	2493.307	15,706,283
	median	100.9061	2195.912	1777.667	1.06 × 10^10^	100	6.82× 10^9^	5,247,378	5423.893	4,281,710	81,580,648	577.9017	4600.499	11,240,071
	rank	2	4	6	13	1	12	8	7	10	11	3	5	9
C17-F3	mean	300	464.5135	302.0192	10,267.95	300	12,685.25	3260.516	300.0635	2593.578	858.8106	10,918.38	315.5806	23,430.61
	best	300	300.1927	300	5527.263	300	8573.571	669.5847	300.0286	329.8934	607.0993	6863.28	313.0498	15,808.63
	worst	300	844.4021	304.319	13,744.58	300	16,868.14	7558.956	300.1108	4362.243	1095.099	14,847.31	319.4459	28,582.79
	std	1.89 × 10^−13^	254.8057	2.342749	3757.315	4.04 × 10^−11^	3775.747	2994.478	0.038787	1853.797	199.5407	3293.007	2.79139	5569.145
	median	300	356.7296	301.8789	10,899.98	300	12,649.64	2406.762	300.0573	2841.088	866.5219	10,981.47	314.9134	24,665.52
	rank	1	6	4	10	2	12	9	3	8	7	11	5	13
C17-F4	mean	403.0484	407.2957	405.0707	1414.847	400	452.2675	452.3776	403.3356	417.5088	429.1642	404.8591	405.1688	413.6139
	best	402.5089	406.6334	401.3246	874.8043	400	408.4697	407.1862	400.7995	407.7759	410.0639	403.8009	400.3294	408.6659
	worst	403.9619	407.9785	406.9653	1943.823	400	484.0319	516.3257	405.041	446.4927	474.1362	406.4845	411.3318	417.5843
	std	0.64045	0.610881	2.657606	456.3131	2.19 × 10^−7^	35.3625	51.19739	1.911616	19.32268	30.32581	1.230735	5.510745	4.388246
	median	402.8615	407.2854	405.9964	1420.38	400	458.2842	442.9992	403.751	407.8834	416.2283	404.5755	404.5069	414.1026
	rank	2	7	5	13	1	11	12	3	9	10	4	6	8
C17-F5	mean	520.0481	517.9215	547.3819	578.3821	524.1491	555.48	565.3388	522.6386	520.2654	532.6729	557.956	531.8387	531.6347
	best	506.9647	514.9671	528.8537	562.5519	510.9445	536.7252	552.0922	512.9368	516.9173	527.4993	552.7326	515.9198	525.2321
	worst	540.3655	520.8979	567.6569	594.5714	545.3806	583.5143	587.2286	540.7974	524.6775	540.5986	570.6416	542.783	537.0252
	std	14.40665	2.916079	20.38514	17.75947	16.24505	19.95166	15.19038	12.38441	3.301751	5.610836	8.573295	12.1854	4.905863
	median	516.4311	517.9106	546.5084	578.2025	520.1356	550.8402	561.0172	518.4101	519.7334	531.2967	554.225	534.326	532.1407
	rank	2	1	9	13	5	10	12	4	3	8	11	7	6
C17-F6	mean	600.0008	600.8465	618.7414	644.0479	623.8526	629.3204	637.3912	601.3458	600.1938	606.3029	618.6179	602.4556	607.4921
	best	600.0001	600.0026	617.6546	640.5716	614.9259	618.7987	627.5706	600.5279	600.1227	604.6819	603.1557	600.4931	605.6483
	worst	600.0024	601.6626	621.5024	648.6505	631.2704	638.4976	643.6766	603.0893	600.3623	607.7053	639.1099	603.3747	608.445
	std	0.001059	0.916838	1.846013	3.630547	6.802204	9.123253	7.55601	1.179296	0.113824	1.335779	16.6334	1.356157	1.252703
	median	600.0003	600.8604	617.9043	643.4847	624.607	629.9926	639.1589	600.8831	600.145	606.4122	616.1031	602.9772	607.9374
	rank	1	3	9	13	10	11	12	4	2	6	8	5	7
C17-F7	mean	714.9232	735.8553	770.0335	812.0262	723.6362	794.4765	804.2404	738.9214	737.1898	748.3656	736.1961	736.6172	742.9681
	best	712.7689	717.4734	746.6222	797.7337	720.2506	781.8441	778.6661	731.8761	720.4588	742.0222	717.1787	725.272	732.2879
	worst	716.4969	759.7501	800.0727	825.7699	726.113	805.1441	842.8269	754.0978	752.0498	753.5933	757.0473	753.4337	750.6899
	std	1.562194	17.9943	24.60583	13.16748	2.810547	11.91296	28.10275	10.33946	12.94484	5.913658	19.72524	12.12503	8.93704
	median	715.2135	733.0988	766.7196	812.3006	724.0905	795.4589	797.7343	734.8559	738.1253	748.9235	735.2791	733.8816	744.4474
	rank	1	3	10	13	2	11	12	7	6	9	4	5	8
C17-F8	mean	816.6276	821.1931	833.5798	858.0199	815.9157	847.5389	838.4987	817.1652	815.6853	831.6274	821.3916	822.884	820.143
	best	803.9798	805.9698	821.8891	845.8319	805.9698	837.1798	816.4064	812.9367	814.2627	821.404	812.9345	817.9092	811.2669
	worst	851.586	853.9211	850.7427	863.7385	827.5538	854.4324	852.3891	821.8906	819.3457	842.065	829.8487	836.8133	827.5287
	std	23.31975	22.21323	12.1958	8.256772	11.06309	7.741079	15.84329	3.666664	2.45444	9.1537	7.197692	9.298061	7.809856
	median	805.4723	812.4407	830.8436	861.2546	815.0695	849.2718	842.5997	816.9166	814.5663	831.5204	821.3916	818.4067	820.8881
	rank	3	6	10	13	2	12	11	4	1	9	7	8	5
C17-F9	mean	900.3851	929.3131	1211.93	1514.186	900	1458.039	1265.415	900.2293	965.985	941.6624	900	1084.117	905.2287
	best	900.015	900.0012	958.169	1410.468	900	990.2664	1008.524	900.0018	900.5441	913.7529	900	900.9737	901.4811
	worst	901.011	971.5927	1724.097	1663.035	900	2444.594	1578.136	900.9101	1155.325	980.4159	900	1338.977	907.1148
	std	0.471982	34.89239	354.9109	107.5417	2.05 × 10^−8^	667.1913	251.4761	0.453875	126.2692	28.21168	0	216.6576	2.557814
	median	900.2572	922.8293	1082.726	1491.62	900	1198.649	1237.501	900.0026	904.0356	936.2405	900	1048.258	906.1594
	rank	4	6	10	13	2	12	11	3	8	7	1	9	5
C17-F10	mean	1136.471	1525.5	1833.3	2692.02	1398.269	2196.056	1831.813	1696.621	1618.109	2333.778	2369.988	1799.815	1931.023
	best	1015.317	1118.755	1517.483	2508.239	1269.37	1628.727	1611.271	1431.721	1460.399	2172.52	2069.602	1548.165	1789.372
	worst	1269.761	2173.755	2515.046	3078.344	1458.036	2789.958	1998.959	2038.82	1803.231	2471.171	2484.022	2102.943	2091.463
	std	104.1524	455.0288	468.3656	265.2632	87.62248	516.1888	161.4886	255.5049	164.4428	130.9142	200.6485	289.059	126.6268
	median	1130.404	1404.745	1650.336	2590.748	1432.834	2182.769	1858.51	1657.972	1604.402	2345.711	2463.163	1774.075	1921.627
	rank	1	3	8	13	2	10	7	5	4	11	12	6	9
C17-F11	mean	1110.292	1124.026	1151.953	4189.355	1101.74	2433.766	1222.852	1122.666	1125.431	1164.599	1141.981	1160.628	1626.893
	best	1106.88	1117.954	1118.262	1484.117	1100.601	1175.783	1164.024	1105.305	1113.134	1157.099	1121.042	1117.92	1359.157
	worst	1113.47	1133.078	1209.026	6861.324	1103.787	5818.316	1345.834	1135.827	1132.354	1175.083	1173.496	1208.451	1865.215
	std	2.815898	6.45982	39.95022	2416.697	1.417748	2258.972	83.82071	12.94547	8.869157	8.65636	22.38728	42.52562	221.4175
	median	1110.408	1122.536	1140.263	4205.989	1101.286	1370.483	1190.775	1124.767	1128.117	1163.106	1136.692	1158.07	1641.6
	rank	2	4	7	13	1	12	10	3	5	9	6	8	11
C17-F12	mean	1242.422	2343.334	1,181,918	7.58 × 10^8^	1233.441	1096,381	4,964,945	370,327	756,766.5	3,736,063	1,095,779	5930.549	913,806.8
	best	1207.99	1359.837	382,250	1.68 × 10^8^	1200.822	38,588.44	1,889,170	17,106.39	235,233.1	616,984.9	509,541	2404.891	132,160.6
	worst	1330.82	4297.499	2,143,478	1.32 × 10^9^	1320.289	2,832,543	10,251,735	674,113.1	1,939,197	5,876,192	1,853,207	11,438.52	2,872,053
	std	59.08526	1323.889	823,784.1	5.85 × 10^8^	58.10221	1,238,741	3,677,451	314,405.5	801,178.8	2,473,613	568,776.2	4251.026	1,315,343
	median	1215.439	1858	1,100,971	7.69E × 10^8^	1206.327	757,195.4	3,859,438	395,044.3	426,317.8	4,225,537	1,010,183	4939.391	325,507
	rank	2	3	10	13	1	9	12	5	6	11	8	4	7
C17-F13	mean	1314.651	1329.426	19,590.41	36,919,805	1306.198	7713.778	17,906.98	6218.485	11,600.44	10,709.37	10,719.01	9477.394	14,731.63
	best	1308.406	1314.537	2827.835	3,065,090	1301.331	5119.752	2690.152	1643.858	7514.508	3941.49	5323.606	2204.365	8697.185
	worst	1318.079	1363.912	33,636.44	1.23 × 10^8^	1310.414	11,977.73	34,896.49	18,348.49	14,579.42	17,269.75	15,137.13	16,209.74	22,062.2
	std	4.420181	23.19983	15,927.29	57,222,140	4.14073	3006.084	15,038.52	8095.684	3088.611	5648.28	4147.954	5798.845	5591.951
	median	1316.06	1319.628	20,948.69	11,023,424	1306.523	6878.815	17,020.65	2440.796	12,153.91	10,813.12	11,207.65	9747.735	14,083.57
	rank	2	3	12	13	1	5	11	4	9	7	8	6	10
C17-F14	mean	1414.72	1423.19	2068.49	5643.20	1404.48	3501.69	3400.44	1493.75	4803.04	1568.78	5878.19	5869.60	5357.57
	best	1412.45	1406.97	1699.95	4926.75	1401.00	1.55 × 10^3^	1648.11	1.4 × 10^3^	4050	1522.97	4842	2264	2517.23
	worst	1419.77	1435.92	2935.30	7310.20	1406.97	5.45 × 10^3^	5862.66	1551	5156	1652.01	8015.69	7283	8985.62
	std	3.390648	12.24421	582.1813	1119.612	2.50	2.21 × 10^3^	2049.513	5	510.9601	59.87624	1.49 × 10^3^	2.41 × 10^3^	2913.649
	median	1413.33	1424.93	1819.36	5167.92	1404.98	3.50 × 10^3^	3.05 × 10^3^	1.49 × 10^3^	5002.87	1550.06	5327.38	6965.79	4963.72
	rank	2	3	6	11	1	8	7	4	9	5	13	12	10
C17-F15	mean	1512.83	1521.97	5583.05	14,802.85	1500.96	7545.17	5337.48	1861.61	3291.73	1839.96	25,559.97	4109.45	5985.44
	best	1511.37	1511.68	2115.43	2826.68	1500.28	1.68 × 10^3^	2420.26	1.54 × 10^3^	1575	1690.59	11,956	1622	3545.59
	worst	1514.98	1528.08	13,461.88	32,524.72	1502.00	2.09 × 10^4^	6967.84	2255	7770	1979.28	38,417.80	6877	8873.14
	std	1.654731	7.536983	5293.321	12967.07	0.79	9.04 × 10^3^	2099.34	3.67 × 10^2^	2997.628	147.988	1.26 × 10^4^	2.55 × 10^3^	2702.172
	median	1512.50	1524.07	3377.44	11,930.01	1500.79	3.80 × 10^3^	5.98 × 10^3^	1.82 × 10^3^	1911.17	1844.99	25,932.99	3968.99	5761.51
	rank	2	3	9	12	1	11	8	5	6	4	13	7	10
C17-F16	mean	1611.84	1631.96	1825.20	2047.53	1601.43	1939.80	1958.00	1810.13	1816.18	1764.56	2108.53	1936.44	1791.70
	best	1607.70	1602.02	1645.36	1835.70	1600.83	1.80 × 10^3^	1845.63	1.61 × 10^3^	1668	1673.94	1973	1842	1728.99
	worst	1615.65	1719.59	1950.57	2341.89	1601.90	2.06 × 10^3^	2123.07	2107	1990	1899.69	2317.95	2037	1848.57
	std	3.460903	58.42219	128.574	213.7822	0.44	1.30 × 10^2^	120.1071	2.10 × 10^2^	140.4967	99.39318	1.57 × 10^2^	87.4	64.59923
	median	1612.00	1603.12	1852.44	2006.27	1601.49	1.95 × 10^3^	1.93 × 10^3^	1.76 × 10^3^	1803.64	1742.32	2071.58	1933.46	1794.62
	rank	2	3	8	12	1	10	11	6	7	4	13	9	5
C17-F17	mean	1716.703	1738.518	1754.813	1827.274	1703.13	2030.624	1805.698	1769.854	1764.037	1782.169	1857.806	1788.933	1757.909
	best	1713.768	1710.808	1736.971	1809.096	1701.435	1769.61	1767.831	1723.549	1722.862	1769.332	1751.548	1742.054	1746.479
	worst	1719.172	1753.339	1802.155	1837.197	1704.773	2407.068	1821.411	1790.076	1797.933	1799.261	1993.65	1864.424	1765.935
	std	2.329847	18.92729	31.64484	12.48988	1.416707	307.1122	25.48385	31.13371	38.49878	15.20238	123.4642	55.24992	8.187043
	median	1716.937	1744.963	1740.062	1831.402	1703.157	1972.91	1816.775	1782.896	1767.677	1780.042	1843.013	1774.627	1759.61
	rank	2	3	4	11	1	13	10	7	6	8	12	9	5
C17-F18	mean	1814.405	1821.362	12,583.12	6,109,172	1801.274	23,279.35	12,052.7	31,257.28	15,733.45	32,735.85	10,284.31	6716.702	11,959.11
	best	1810.809	1808.227	5064.639	302,305.5	1800.582	14,032.49	6236.408	7496.333	2186.154	13,630.19	6724.069	4646.373	3903.202
	worst	1819.123	1830.204	16,593.21	17,734,831	1801.954	36,612.74	20,512.16	46,205.61	35,629.02	48,828.46	12,582.88	11,176.9	33,403.2
	std	3.560254	10.39659	5168.715	8,077,112	0.638221	9630.484	6341.297	16,873.78	14,345.79	15,253.63	2500.631	3067.479	14,314.21
	median	1813.843	1823.509	14,337.31	3,199,777	1801.279	21,236.09	10,731.12	35,663.58	12,559.31	34,242.38	10,915.15	5521.766	5265.028
	rank	2	3	8	13	1	10	7	11	9	12	5	4	6
C17-F19	mean	1900.829	1906.001	7055.795	754,594.9	1988.096	7862.419	11,722.22	1924.021	3422.268	2919.764	43,205.63	12,423.58	8045.107
	best	1900.197	1902.859	2196.819	48,985.81	1941.345	1936.761	2247.991	1904.848	1931.079	2082.118	11,768.13	1992.972	2511.267
	worst	1901.132	1911.477	14,063.29	1621,173	2033.869	14,054.72	35,401.61	1943.471	7740.098	5097.511	62,729.71	32,949.66	13,550.94
	std	0.426417	4.051826	5770.348	709,246.5	37.97119	6845.286	15,850.82	15.98525	2879.051	1456.032	22,823.33	14,370.32	5195.004
	median	1900.994	1904.834	5981.538	674,110.5	1988.585	7729.098	4619.639	1923.882	2008.947	2249.714	49,162.35	7375.847	8059.112
	rank	1	2	7	13	4	8	10	3	6	5	12	11	9
C17-F20	mean	2011.485	2042.536	2182.848	2239.101	2108.71	2113.828	2117.662	2049.031	2069.477	2103.812	2271.99	2148.104	2046.167
	best	2003.98	2020.622	2033.57	2176.378	2029.565	2032.586	2074.137	2023.77	2040.489	2070.52	2201.309	2086.369	2040.594
	worst	2024.81	2065.244	2315.735	2298.524	2143.217	2186.926	2165.246	2088.018	2141.267	2190.885	2371.812	2206.599	2054.028
	std	9.604069	23.49206	126.9386	60.11148	53.07689	71.16499	38.70398	30.47418	48.05391	58.21754	82.95245	49.2503	6.047881
	median	2008.575	2042.138	2191.043	2240.75	2131.029	2117.899	2115.632	2042.168	2048.075	2076.922	2257.419	2149.723	2045.023
	rank	1	2	11	12	7	8	9	4	5	6	13	10	3
C17-F21	mean	2200	2257.378	2214.815	2272.021	2285.241	2370.129	2286.841	2318.856	2316.059	2333.48	2380.593	2298.441	2248.355
	best	2200	2201.776	2204.429	2225.704	2256.272	2357.389	2208.473	2312.628	2314.555	2320.683	2361.841	2202.641	2211.672
	worst	2200	2315.502	2241.859	2298.356	2313.686	2395.748	2352.572	2328.8	2317.369	2339.95	2399.185	2341.226	2334.061
	std	4.36 × 10^−6^	63.34808	18.08569	32.13155	30.34504	17.75696	74.57407	7.057306	1.33189	8.678521	15.60657	64.84178	57.45861
	median	2200	2256.116	2206.485	2282.012	2285.503	2363.69	2293.16	2316.999	2316.157	2336.644	2380.674	2324.948	2223.844
	rank	1	4	2	5	6	12	7	10	9	11	13	8	3
C17-F22	mean	2293.452	2304.053	2309.64	2961.228	2300.478	2852.928	2310.94	2447.659	2303.451	2323.176	2300	2590.674	2315.89
	best	2249.619	2302.865	2304.685	2736.917	2300.289	2325.93	2306.026	2303.655	2301.229	2317.806	2300	2301.37	2312.981
	worst	2309.573	2306.702	2311.968	3125.843	2300.639	4289.031	2315.364	2876.013	2308.429	2330.145	2300	3455.61	2322.759
	std	29.29682	1.78132	3.347251	163.7741	0.148633	958.3573	3.841076	285.5712	3.34564	5.128153	4.79 × 10^−11^	576.6258	4.631892
	median	2307.308	2303.322	2310.953	2991.077	2300.493	2398.375	2311.184	2305.484	2302.074	2322.376	2300	2302.858	2313.91
	rank	1	5	6	13	3	12	7	10	4	9	2	11	8
C17-F23	mean	2610.573	2629.183	2645.231	2708.158	2612.224	2726.841	2653.024	2616.866	2616.438	2639.373	2806.265	2640.991	2667.811
	best	2607.664	2617.394	2632.674	2676.838	2606.93	2674.586	2628.832	2605.735	2605.898	2633.039	2736.331	2624.248	2659.715
	worst	2612.054	2640.741	2664.384	2751.868	2615.963	2799.207	2684.875	2626.638	2621.972	2644.202	2955.197	2656.69	2674.554
	std	1.994539	10.55426	14.9135	35.0647	4.480225	56.75694	23.83249	9.040098	7.270681	5.590693	102.8952	14.70599	7.17143
	median	2611.287	2629.299	2641.933	2701.962	2613.002	2716.785	2649.195	2617.545	2618.941	2640.125	2766.766	2641.513	2668.487
	rank	1	5	8	11	2	12	9	4	3	6	13	7	10
C17-F24	mean	2500	2628.429	2777.971	2866.473	2569.191	2809.687	2721.283	2747.647	2747.38	2702.715	2798.085	2766.089	2796.904
	best	2500	2500.026	2749.551	2831.736	2500	2788.515	2539.368	2741.391	2733.965	2519.228	2667.104	2743.202	2777.411
	worst	2500	2756.728	2810.839	2925.445	2644.684	2842.916	2793.127	2760.786	2763.043	2765.55	2910.085	2813.42	2837.744
	std	0.000138	148.1166	27.63919	40.74193	59.26318	24.23185	121.6661	8.946334	13.17159	122.3327	99.80776	31.95768	28.33329
	median	2500	2628.481	2775.747	2854.356	2566.041	2803.659	2776.318	2744.206	2746.256	2763.041	2807.575	2753.866	2786.23
	rank	1	3	9	13	2	12	5	7	6	4	11	8	10
C17-F25	mean	2906.512	2922.277	2912.059	3302.497	2897.744	3106.26	2952.732	2944.896	2936.407	2928.564	2921.498	2929.4	2955.349
	best	2899.622	2897.934	2899.173	3227.936	2897.743	2996.274	2950.071	2943.625	2913.914	2903.231	2899.585	2899.585	2949.724
	worst	2914.472	2946.585	2949.468	3386.778	2897.746	3238.662	2957.737	2946.057	2946.069	2950.815	2943.426	2950.481	2961.557
	std	7.989653	27.1567	24.94164	65.75238	0.001401	109.1914	3.436649	1.280875	15.0943	24.7158	25.30253	22.9263	4.8434
	median	2905.976	2922.294	2899.797	3297.637	2897.743	3095.053	2951.561	2944.951	2942.823	2930.105	2921.49	2933.766	2955.059
	rank	2	5	3	13	1	12	10	9	8	6	4	7	11
C17-F26	mean	2671.195	2913.423	2985.864	3820.788	2675.001	3616.499	3543.485	2894.319	3162.049	3024.196	3934.065	2953.575	2984.157
	best	2617.932	2817.326	2800	3472.539	2600.001	3138.702	3155.692	2801.236	2817.027	2962.221	2800	2900	2918.692
	worst	2822.654	3035.347	3176.148	4183.224	2900	4076.951	3938.706	2975.736	3958.769	3149.034	4458.174	3043.137	3058.781
	std	100.9928	90.24848	214.6477	306.4302	149.9997	384.0996	344.4273	71.55693	534.9178	84.63646	768.3315	68.48644	71.71749
	median	2622.096	2900.509	2983.654	3813.695	2600.001	3625.172	3539.772	2900.152	2936.199	2992.764	4239.043	2935.581	2979.577
	rank	1	4	7	12	2	11	10	3	9	8	13	5	6
C17-F27	mean	3089.249	3108.049	3122.299	3241.788	3230.617	3156.931	3131.995	3128.978	3112.27	3107.927	3236.309	3104.619	3158.594
	best	3088.978	3097.47	3095.743	3130.053	3089.518	3099.004	3100.639	3089.735	3094.181	3094.735	3223.231	3096.887	3110.429
	worst	3089.518	3119.426	3187.808	3448.33	3302.844	3201.841	3212.034	3245.764	3138.824	3143.478	3259.486	3115.015	3195.857
	std	0.310716	9.908994	43.79996	141.0072	97.5953	51.24289	53.52643	77.85813	20.82847	23.73436	16.13351	7.657847	36.95242
	median	3089.25	3107.651	3102.823	3194.385	3265.053	3163.44	3107.654	3090.206	3108.038	3096.747	3231.259	3103.287	3164.045
	rank	1	4	6	13	11	9	8	7	5	3	12	2	10
C17-F28	mean	3100	3206.371	3298.81	3829.484	3216.221	3436.214	3311.827	3335.937	3320.677	3445.166	3476.706	3368.271	3180.566
	best	3100	3101.23	3172.905	3741.083	3100	3216.368	3185.855	3164.423	3227.504	3224.747	3462.464	3239.91	3150.246
	worst	3100	3383.883	3411.822	3893.295	3283.338	3554.863	3436.561	3411.825	3433.953	3731.813	3496.499	3446.48	3250.807
	std	7.13 × 10^−5^	125.1841	97.91492	70.64911	81.82053	153.671	130.6719	115.1056	103.9361	210.5104	15.77081	89.50779	47.68498
	median	3100	3170.186	3305.256	3841.779	3240.773	3486.812	3312.445	3383.749	3310.626	3412.052	3473.932	3393.348	3160.605
	rank	1	3	5	13	4	10	6	8	7	11	12	9	2
C17-F29	mean	3138.606	3179.875	3296.049	3393.557	3289.561	3381.154	3360.139	3232.25	3245.584	3197.841	3362.024	3221.345	3231.314
	best	3131.359	3164.605	3216.476	3316.959	3206.422	3329.678	3226.82	3135.375	3159.205	3187.325	3241.182	3171.9	3222.514
	worst	3148.378	3205.527	3382.815	3465.973	3346.892	3413.532	3430.368	3320.002	3367.448	3209.226	3673.067	3259.484	3241.71
	std	7.707765	18.65754	85.63264	76.78127	60.23812	39.15099	91.66353	86.28562	90.14385	11.73545	208.2333	37.86476	8.061759
	median	3137.343	3174.685	3292.453	3395.648	3302.466	3390.704	3391.683	3236.811	3227.843	3197.407	3266.924	3226.999	3230.516
	rank	1	2	9	13	8	12	10	6	7	3	11	4	5
C17-F30	mean	3396.648	7103.166	315,325.2	3,935,476	3593.41	228,769.3	1854,829	18,378.17	25,404.81	328,679.2	837,788.8	434,361.7	2254,186
	best	3395.218	4030.14	111,836.1	885,863.3	3485.918	9138.959	207,480.4	6468.614	10,065.88	44,946.47	644,043.4	5474.912	282,133.6
	worst	3397.324	15,735.06	821,835.5	6,216,163	3634.301	458,333.2	5,681,033	35,678.41	34,710.86	821,511	1069,950	1,705,095	4,266,383
	std	0.972309	5757.626	338,593.4	2,231,806	71.81478	185,592.7	2,567,825	12,749.49	11,597.43	352,907.1	176,988.7	847,168.3	1,783,824
	median	3397.024	4323.731	163,814.6	4,319,938	3626.71	223,802.5	765,402.1	15,682.84	28,421.26	224,129.6	818,581	13,438.71	2,234,113
	rank	1	3	7	13	2	6	11	4	5	8	10	9	12
Sum rank		46	106	215	356	87	303	272	160	182	218	265	206	223
Mean rank		1.586207	3.655172	7.413793	12.27586	3	10.44828	9.37931	5.517241	6.275862	7.517241	9.137931	7.103448	7.689655
Total rank		1	3	7	13	2	12	11	4	5	8	10	6	9

**Table 3:** Optimization results of the CEC 2017 test suite (for the dimension d=30).

		SOA	WSO	AVOA	RSA	MPA	TSA	WOA	MVO	GWO	TLBO	GSA	PSO	GA
C17-F1	mean	1575.134	8.63 × 10^9^	3318.344	4.38 × 10^10^	44,342.91	2.31 × 10^10^	2.19 × 10^9^	660,553.8	2.3 × 10^9^	6.17 × 10^9^	11,193,471	2 × 10^9^	1.32 × 10^8^
	best	291.5791	3.2 × 10^9^	301.9834	3.91 × 10^10^	23,422.06	1.8 × 10^10^	1.14 × 10^9^	489,540.5	1.48 × 10^9^	5.57 × 10^9^	2775.087	5373.926	84,091,185
	worst	2723.003	1.55 × 10^10^	8149.68	5.39 × 10^10^	73,308.85	2.69 × 10^10^	4.06 × 10^9^	1,043,985	3.25 × 10^9^	7.22 × 10^9^	39,077,336	6.32 × 10^9^	2.15 × 10^8^
	std	1312.344	5.69 × 10^9^	3675.955	6.83 × 10^9^	23,716.31	3.78 × 10^9^	1.36 × 10^9^	257,939.3	7.37 × 10^8^	7.68 × 10^8^	18,781,636	2.98 × 10^9^	57,126,045
	median	1642.976	7.91 × 10^9^	2410.857	4.11 × 10^10^	40,320.37	2.38 × 10^10^	1.77 × 10^9^	554,344.8	2.24 × 10^9^	5.95 × 10^9^	2,846,886	8.4 × 10^8^	1.15 × 10^8^
	rank	1	11	2	13	3	12	8	4	9	10	5	7	6
C17-F3	mean	781.8758	42,375.1	47,609.78	78,405.91	834.2454	48,537.76	225,225.5	1031.676	55,393.14	36,906.86	102,114.9	77,694.12	134,031.2
	best	470.0985	31,992.7	25,840.42	60,713.72	466.3567	36,205.28	165,937.7	751.8445	42,537.2	28,114.76	87,911.84	5616.74	91,331.46
	worst	1159.523	56,799.39	61,573.93	85,173.33	1251.796	60,139.23	339,833.4	1377.855	69,178.02	44,671.67	112,455.1	122,259.2	171,056.6
	std	363.8149	11,011.05	15,321.68	11,832.89	426.2832	11,184.08	79,056.19	259.0859	14,094.56	8186.672	11,070.13	52,140.19	33,150.42
	median	748.9407	40,354.16	51,512.39	83,868.3	809.4147	48,903.27	197,565.4	998.5019	54,928.67	37,420.51	104,046.4	91,450.26	136,868.4
	rank	1	5	6	10	2	7	13	3	8	4	11	9	12
C17-F4	mean	481.8263	841.7389	518.3661	10,440.38	495.7528	2158.726	841.9903	495.544	567.0971	827.2028	603.7402	504.0106	813.8432
	best	469.5735	740.4671	494.0059	6680.906	482.257	1388.877	664.6617	488.5097	532.196	630.1235	582.046	478.9838	724.672
	worst	496.1271	1012.754	537.7292	14,606.03	517.0849	2948.487	1220.665	504.9644	623.5882	1298.773	628.6736	536.8896	894.6409
	std	12.21788	127.7259	18.14231	3289.65	15.86538	664.9026	255.0823	7.418566	39.35261	316.7743	20.33317	24.07526	71.3288
	median	480.8022	806.8675	520.8646	10237.29	491.8346	2148.769	741.317	494.3509	556.3021	689.9572	602.1205	500.0845	818.03
	rank	1	10	5	13	3	12	11	2	6	9	7	4	8
C17-F5	mean	591.9605	653.5151	740.563	909.9791	604.5444	853.4376	764.4085	616.2858	623.7568	746.731	737.7933	685.3203	706.2255
	best	572.8715	603.0483	700.9927	881.9704	597.5869	831.9647	737.6831	577.9794	610.044	696.6675	716.8998	620.4016	695.0019
	worst	609.7335	691.464	803.4606	946.4631	612.4978	888.9031	787.6346	671.5395	645.0115	779.7375	765.6516	768.6397	714.7736
	std	17.43398	39.32124	46.32888	30.80919	6.734852	24.70772	24.55298	39.50365	15.98556	36.95873	21.72216	61.70543	9.752587
	median	592.6185	659.7741	728.8994	905.7414	604.0464	846.4413	766.1582	607.8121	619.9859	755.2595	734.3109	676.12	707.5632
	rank	1	5	9	13	2	12	11	3	4	10	8	6	7
C17-F6	mean	606.6229	644.2901	649.6843	688.3251	603.2172	667.3338	677.7711	623.4261	610.7615	641.7919	660.113	650.2099	640.5046
	best	605.5854	639.7766	647.5717	682.6749	601.8096	648.0678	656.5877	605.7405	604.005	636.0484	659.3197	643.6113	632.6248
	worst	608.634	653.2473	652.9881	695.4187	605.8092	682.7658	699.2122	638.1243	616.1276	646.9417	661.167	664.0501	649.3386
	std	1.435549	6.086035	2.340959	5.885883	1.859946	16.20593	17.91275	14.59694	5.139016	4.469085	0.814301	9.348912	7.046186
	median	606.1361	642.0682	649.0887	687.6034	602.6249	669.2509	677.6422	624.9199	611.4567	642.0888	659.9828	646.5891	640.0274
	rank	2	7	8	13	1	11	12	4	3	6	10	9	5
C17-F7	mean	824.4019	1028.891	1173.837	1378.659	871.1627	1261.634	1237.611	877.7531	902.3727	1050.815	987.1371	961.4578	998.7671
	best	808.2562	937.0001	1050.146	1364.064	830.9612	1217.349	1191.509	830.7576	869.2935	1023.079	936.652	940.4088	990.4792
	worst	840.1661	1166.637	1346.571	1403.463	963.2763	1286.653	1300.094	923.0444	952.51	1093.22	1062.44	991.3926	1006.751
	std	15.87142	97.43161	130.9887	17.50672	62.07233	30.40281	53.17939	38.72923	40.18853	33.72067	55.15938	24.88631	6.666849
	median	824.5927	1005.964	1149.315	1373.554	845.2067	1271.267	1229.42	878.6053	893.8436	1043.48	974.7284	957.015	998.9192
	rank	1	8	10	13	2	12	11	3	4	9	6	5	7
C17-F8	mean	876.44	921.3526	959.7397	1143.549	905.3425	1109.568	1022.72	918.4966	931.9204	1052.634	972.3755	928.3456	1000.158
	best	861.6931	896.2341	927.3542	1121.49	882.7297	1050.063	974.1633	894.9188	904.3727	1014.92	946.2582	899.4956	989.1129
	worst	901.5509	949.9888	983.0733	1172.767	930.5994	1173.263	1072.325	929.7411	960.3594	1079.362	1000.98	953.2309	1019.463
	std	18.96082	22.04258	25.26299	25.97783	22.14314	51.04285	42.15311	16.41749	25.40171	27.15429	24.10391	23.49774	13.27682
	median	871.258	919.5937	964.2657	1139.97	904.0204	1107.473	1022.196	924.6632	931.4748	1058.127	971.1318	930.3279	996.027
	rank	1	4	7	13	2	12	10	3	6	11	8	5	9
C17-F9	mean	1195.591	6940.902	5082.067	11,265.22	1229.29	14,700.33	12,240.59	3886.39	2149.413	4773.832	4288.668	4170.381	1390.886
	best	1005.433	5371.93	3739.922	10,986.11	1039.082	12,687.95	8790.439	2585.385	1625.875	4462.957	3715.719	1685.736	1257.047
	worst	1413.609	9175.404	5799.199	11,406.53	1446.192	17,328.07	16,223.67	6664.175	2800.198	5128.325	5171.329	6392.186	1505.439
	std	174.8498	1601.414	921.6272	189.314	172.3628	2274.191	3233.82	1919.637	510.5671	310.8324	641.2085	1950.664	125.7143
	median	1181.661	6608.138	5394.574	11,334.13	1215.943	14,392.65	11,974.14	3148	2085.789	4752.023	4133.811	4301.802	1400.529
	rank	1	10	9	11	2	13	12	5	4	8	7	6	3
C17-F10	mean	3816.318	5075.775	5669.471	8287.882	4220.886	7602.726	6780.741	5112.877	4492.313	8516.982	5023.915	5002.451	6061.83
	best	3461.682	4293.963	4863.102	7401.561	3924.042	6749.686	6256.547	4322.227	3596.22	8229.134	4753.717	4206.05	5451.612
	worst	4025.525	6408.048	6213.965	8953.203	4725.856	8093.557	7076.522	6028.47	5426.175	8698.705	5457.712	5740.762	6971.01
	std	268.2071	933.6489	647.4401	649.1055	362.4291	596.9208	382.085	785.8896	962.8636	207.2443	323.6773	627.4781	645.3234
	median	3889.033	4800.545	5800.408	8398.382	4116.823	7783.83	6894.948	5050.406	4473.429	8570.044	4942.116	5031.496	5912.35
	rank	1	6	8	12	2	11	10	7	3	13	5	4	9
C17-F11	mean	1162.133	1448.116	1268.696	9328.214	1171.813	5380.791	6344.694	1341.146	1947.539	1897.276	3021.119	1401.099	5138.217
	best	1142.585	1297.005	1196.704	7579.907	1132.01	3668.83	4242.53	1265.451	1440.926	1744.854	2320.74	1164.748	1545.325
	worst	1182.493	1551.242	1337.29	10,508.06	1215.997	6958.553	9436.31	1429.885	2921.548	2074.605	3738.218	1770.983	9100.655
	std	18.852	121.068	58.60946	1341.609	41.78269	1638.069	2277.368	82.79307	687.1649	140.7209	668.1668	268.493	3518.582
	median	1161.728	1472.108	1270.394	9612.443	1169.622	5447.89	5849.969	1334.624	1713.841	1884.822	3012.758	1334.332	4953.445
	rank	1	6	3	13	2	11	12	4	8	7	9	5	10
C17-F12	mean	90,577.25	85,200,129	22,294,766	1.17 × 10^10^	90,003.89	4.13 × 10^9^	1.9 × 10^8^	16,023,229	30,541,462	3.65 × 10^8^	2.13 × 10^8^	436,028.7	7,615,346
	best	53,334.11	6,278,481	3,137,139	1.04 × 10^10^	53,008.9	8.42 × 10^8^	54,166,306	1969,767	2402,615	2.99 × 10^8^	41,143,265	236,773	5,611,464
	worst	186,847.7	1.92 × 10^8^	54,450,458	1.47 × 10^10^	185,636.8	9.43 × 10^9^	3.02 × 10^8^	27,669,712	90,151,780	4.98 × 10^8^	6.8 × 10^8^	912,580.8	9,655,726
	std	64,578.16	77,647,077	22,587,307	2.04 × 10^9^	64,148.09	3.69 × 10^9^	1.08 × 10^8^	10,686,736	40,183,994	90,619,476	3.12 × 10^8^	320,014.9	1,658,891
	median	61,063.6	71,256,869	15,795,734	1.08 × 10^10^	60,684.91	3.12 × 10^9^	2.03 × 10^8^	17,226,719	14,805,726	3.3 × 10^8^	65,228,755	297,380.4	7,597,098
	rank	2	8	6	13	1	12	9	5	7	11	10	3	4
C17-F13	mean	1698.702	42,943.25	159,812.1	1.13 × 10^10^	1687.016	3.08 × 10^9^	1880,155	115,014	806,958.5	50,671,827	38,902.02	19,073,995	8,631,128
	best	1666.692	15,453.39	88,435.66	5.92 × 10^9^	1655.899	3.01 × 10^8^	874,962.7	43,649.1	90,835.15	24,346,775	31,538.43	10,348.01	5,437,187
	worst	1739.004	69,114.37	252,818.6	1.38 × 10^10^	1727.879	4.75 × 10^9^	3,971,612	152,301.5	2,153,200	79,648,604	56,967.6	71,682,069	11,994,455
	std	30.25763	29,451.87	68,264.23	3.62 × 10^9^	30.04096	1.98 × 10^9^	1,425,344	48,532.06	945,863.6	22,741,505	12,177.81	35,135,804	2,736,987
	median	1694.557	43,602.61	148,997.1	1.27 × 10^10^	1682.144	3.64 × 10^9^	1,337,022	132,052.8	491,899.7	49,345,965	33,551.03	2,301,781	8,546,436
	rank	2	4	6	13	1	12	8	5	7	11	3	10	9
C17-F14	mean	1444.401	1676.266	289,413.4	2,344,304	1496.974	605,895.5	4,052,315	31,475.33	80,820.24	125,817.1	1220,811	40,538.98	1389,524
	best	1432.619	1509.528	40,389.43	1,178,223	1482.994	19,646.14	500,090.9	1774.484	3031.162	56,550.37	792,133.3	5098.106	79,752
	worst	1454.965	1968.988	670,169.8	3,490,954	1528.7	2,259,238	10,915,286	81,318.86	304,629.3	166,885.1	1843,312	113,632	2,018,125
	std	9.363157	205.8048	277,873.9	1,112,536	21.28861	1,102,518	4,662,123	34,996.97	149,234.9	51,967.8	494,689.9	49,872.43	884,978.4
	median	1445.009	1613.274	223,547.2	2,354,020	1488.101	72,349.17	2,396,942	21,403.99	7810.246	139,916.5	1,123,899	21,712.91	1,730,110
	rank	1	3	8	12	2	9	13	4	6	7	10	5	11
C17-F15	mean	1607.963	4778.296	39,852.03	6.38 × 10^8^	1689.005	11,487,233	2179,861	42,062.9	55,302.7	6330,079	17,042.7	22,342.61	1,660,817
	best	1597.136	2049.811	11,559.75	5.5 × 10^8^	1662.636	510,798.9	41,148.44	27,984.36	29,781.28	1782,675	12,076.24	9161.007	162,991.5
	worst	1620.23	7380.576	64,784.18	7.04 × 10^8^	1713.512	30,936,722	6697,059	53,948.33	95,876.74	10,145,977	23,149.44	43,566.9	2,552,150
	std	11.79297	2216.528	22,477.62	75,373,693	26.43209	14,324,358	3053,330	10,716.84	28,402.06	3805,854	4629.296	16,392.76	1,046,225
	median	1607.243	4841.398	41532.1	6.48 × 10^8^	1689.935	7,250,705	990,617.7	43,159.45	47,776.39	6,695,832	16,472.57	18,321.27	1,964,064
	rank	1	3	6	13	2	12	10	7	8	11	4	5	9
C17-F16	mean	1938.024	2652.321	3090.893	5197.758	2043.515	3941.647	3856.78	2718.081	2844.053	3652.155	3800.762	2579.706	2881.999
	best	1875.092	2467.942	2618.661	4355.175	1960.684	2696.154	3503.463	2471.407	2350.898	2855.694	3606.06	2182.495	2536.668
	worst	2022.866	2873.364	3646.496	5943.335	2140.407	5949.276	4712.045	3124.71	3696.224	4147.742	3977.124	3007.692	3235.006
	std	63.16545	180.599	422.6948	848.5357	76.10694	1411.044	573.5086	297.6959	587.7307	555.942	165.3951	348.332	306.3028
	median	1927.069	2633.988	3049.207	5246.261	2036.484	3560.579	3605.805	2638.104	2664.545	3802.591	3809.932	2564.319	2878.16
	rank	1	4	8	13	2	12	11	5	6	9	10	3	7
C17-F17	mean	1852.902	2208.309	2527.865	3850.265	1842.558	3293.211	2983.695	2140.944	2036.312	2268.715	2581.32	2297.254	2189.114
	best	1788.722	2004.347	2371.871	3442.105	1779.719	2060.002	2607.115	2039.798	1924.983	2092.269	2474.207	2111.585	2010.935
	worst	1928.103	2579.523	2652.707	4571.363	1922.001	5754.239	3280.496	2263.674	2223.179	2603.348	2743.277	2433.751	2310.59
	std	71.09131	262.7278	122.841	511.0619	71.75205	1677.199	286.4468	106.3038	138.6211	232.8375	131.3936	136.9696	131.9916
	median	1847.391	2124.683	2543.44	3693.796	1834.256	2679.302	3023.584	2130.153	1998.542	2189.622	2553.898	2321.84	2217.466
	rank	2	6	9	13	1	12	11	4	3	7	10	8	5
C17-F18	mean	1938.93	73,105.14	2,825,586	34,857,872	1925.262	1277,923	12,144,452	500,241.1	1900,915	1,703,500	549,137.3	690,457.8	4,288,985
	best	1893.579	35,316.23	300,783.8	11,269,502	1876.939	562,332.5	5149,402	138,664.6	227,445.9	539,938.8	307,806.8	65,741.96	1,298,945
	worst	2006.212	126,012.8	5,637,821	68,482,148	1993.891	1,910,634	31,414,794	794,063.9	3,767,376	2,823,794	1,069,302	1,843,343	7,761,510
	std	55.16086	43,259.8	2,501,283	24,260,902	55.11681	631,242.3	12,853,387	306,007.1	1,464,392	954,837.7	351,297.8	816,443.5	2,854,320
	median	1927.964	65,545.78	2,681,870	29,839,919	1915.11	1,319,364	6,006,807	534,118	1,804,419	1,725,134	409,720.2	426,373.2	4,047,743
	rank	2	3	10	13	1	7	12	4	9	8	5	6	11
C17-F19	mean	1950.448	7634.682	72,080.61	1.04 × 10^9^	1934.536	46,099,123	16,697,045	566,616.3	776,641.6	6,505,032	87,089.66	155,575	1,002,429
	best	1944.048	4603.741	15,265.2	7.54 × 10^8^	1925.933	1968,442	1190,037	237,497.3	45,758.44	5,013,834	47,176.61	2541.803	178,602.2
	worst	1956.475	9305.339	160,723	1.58 × 10^9^	1940.446	1.4 × 10^8^	42,887,049	962,525.9	2,874,146	8,511,539	117,244.7	526,626	2,377,684
	std	6.70424	2126.603	63,361.95	3.68 × 10^8^	6.19493	64,769,055	18,312,639	316,644.1	1,398,964	1,468,164	29,169.63	249,691.1	964,832.8
	median	1950.635	8314.824	56,167.14	9.2 × 10^8^	1935.883	21,268,185	11,355,547	533,220.9	93,330.9	6,247,378	91,968.68	46,566.05	726,714.7
	rank	2	3	4	13	1	12	11	7	8	10	5	6	9
C17-F20	mean	2189.442	2360.68	2678.297	3018.944	2202.08	2907.819	2744.225	2463.574	2502.458	2699.637	3079.815	2833.366	2438.403
	best	2134.196	2288.632	2510.461	2829.558	2121.756	2570.504	2403.758	2140.461	2283.051	2586.227	2680.886	2259.638	2241.364
	worst	2259.836	2498.568	2933.844	3140.294	2261.922	3136.657	2907.139	2802.38	2617.604	2888.94	3587.357	3572.419	2528.431
	std	57.14654	94.38145	186.4711	133.7757	60.93235	261.575	235.0817	303.3727	149.2588	136.6338	379.9235	552.2857	132.8408
	median	2181.868	2327.759	2634.442	3052.963	2212.321	2962.058	2833.001	2455.727	2554.588	2661.69	3025.509	2750.703	2491.908
	rank	1	3	7	12	2	11	9	5	6	8	13	10	4
C17-F21	mean	2334.267	2451.525	2451.182	2708.062	2372.321	2623.763	2600.99	2418.065	2435.549	2544.532	2590.171	2440.405	2497.362
	best	2229.608	2427.241	2210.563	2624.032	2364.018	2578.473	2539.261	2385.897	2403.354	2514.163	2570.64	2423.64	2490.605
	worst	2384.456	2472.696	2619.705	2807.897	2387.931	2707.121	2653.493	2460.016	2517.673	2556.921	2628.386	2466.822	2506.684
	std	70.60764	20.94728	172.4367	79.98545	10.64284	58.08507	49.47883	31.62808	55.05508	20.48386	25.92502	19.61484	6.981597
	median	2361.502	2453.082	2487.23	2700.16	2368.668	2604.729	2605.603	2413.174	2410.585	2553.522	2580.83	2435.579	2496.079
	rank	1	7	6	13	2	12	11	3	4	9	10	5	8
C17-F22	mean	2332.529	3959.433	6059.49	8152.075	2330.163	7394.703	7190.862	6119.96	4511.888	8205.841	6631.627	4407.861	2804
	best	2302.568	2653.991	2403.992	7043.939	2303.498	4181.538	3233.91	4962.584	2908.358	3396.988	4129.775	2417.459	2740.193
	worst	2373.482	5235.829	7475.833	9265.812	2405.022	9991.897	9030.261	7463.774	6446.611	10,454	7731.139	7039.898	2875.878
	std	35.41728	1461.304	2441.084	954.9404	49.93237	2954.165	2683.669	1031.328	1696.022	3239.01	1678.839	2265.566	62.5268
	median	2327.034	3973.957	7179.067	8149.275	2306.067	7702.689	8249.638	6026.741	4346.291	9486.189	7332.796	4087.044	2799.965
	rank	2	4	7	12	1	11	10	8	6	13	9	5	3
C17-F23	mean	2691.435	3077.843	2949.293	3294.999	2826.529	3,,206.505	3089.699	2776.37	2843.433	2903.634	3858.628	2957.034	2950.425
	best	2679.378	2941.813	2831.233	3236.012	2780.905	3073.12	3021.808	2743.748	2731.346	2892.146	3741.567	2853.239	2899.501
	worst	2704.098	3173.198	3136.276	3380.292	2889.29	3316.166	3188.281	2813.701	2939.296	2918.309	3974.499	3093.225	3010.503
	std	10.10131	100.1635	133.6444	62.73727	46.7791	101.8644	72.42882	36.38104	100.4405	11.04206	123.4437	101.7865	45.64687
	median	2691.131	3098.181	2914.831	3281.845	2817.96	3218.368	3074.354	2774.014	2851.546	2902.041	3859.223	2940.837	2945.848
	rank	1	9	6	12	3	11	10	2	4	5	13	8	7
C17-F24	mean	2896.226	3408.484	3199.101	3463.698	2877.119	3391.269	3316.346	2915.695	2958.944	3090.075	3408.418	3091.683	3257.36
	best	2888.001	3305.505	3048.832	3366.821	2870.935	3343.024	3234.967	2877.571	2894.125	3059.462	3368.189	3016.899	3199.87
	worst	2902.589	3537.417	3367.271	3632.006	2884.633	3440.512	3459.107	2948.554	3049.884	3125.528	3449.345	3136.157	3339.026
	std	6.731113	96.59341	139.9192	122.5211	5.839244	51.92828	98.12749	34.76358	65.36341	29.5475	35.88437	54.9917	59.44844
	median	2897.156	3395.506	3190.151	3427.983	2876.453	3390.77	3285.656	2918.328	2945.884	3087.655	3408.07	3106.838	3245.273
	rank	2	12	7	13	1	10	9	3	4	5	11	6	8
C17-F25	mean	2912.942	3083.84	2910.493	4702.903	2906.992	3544.987	3087.454	2893.786	2978.111	3041.552	3004.331	2912.146	3085.075
	best	2909.21	3059.773	2895.096	4048.889	2890.234	3041.758	3014.644	2883.757	2932.571	2966.697	2991.811	2888.122	3052.956
	worst	2917.091	3116.934	2952.85	5574.617	2948.672	4212.467	3186.74	2913.858	3018.136	3093.076	3017.914	2942.563	3105.734
	std	3.759012	26.82682	28.25826	635.4398	27.9838	489.9687	74.05925	13.61994	37.24705	55.78418	10.77907	27.8468	23.07507
	median	2912.734	3079.325	2897.013	4594.053	2894.53	3462.861	3074.216	2888.764	2980.868	3053.217	3003.8	2908.95	3090.805
	rank	5	9	3	13	2	12	11	1	6	8	7	4	10
C17-F26	mean	2893.811	7030.026	7628.776	10,262.03	2900.274	7838.24	8165.049	4768.338	4910.815	5876.503	7785.852	4194.966	4581.948
	best	2807.968	5403.73	6234.854	9352.232	2900.076	6226.954	7340.114	4402.959	4478.258	3765.572	6650.013	2819.941	4133.612
	worst	2927.204	8050.923	8445.905	11,868.69	2900.493	9673.83	8975.654	5108.717	5422.342	7143.24	8377.255	6371.495	4920.061
	std	57.33427	1138.534	973.1733	1181.029	0.207894	1474.61	738.9123	347.4211	482.6334	1477.575	805.9973	1521.981	346.2337
	median	2920.037	7332.725	7917.173	9913.604	2900.263	7726.088	8172.214	4780.839	4871.329	6298.6	8058.069	3794.215	4637.06
	rank	1	8	9	13	2	11	12	5	6	7	10	3	4
C17-F27	mean	3215.3	3427.03	3367.647	3811.95	3329.272	3609.212	3470.991	3235.764	3266.848	3319.738	5117.272	3295.73	3464.964
	best	3201.255	3331.043	3273.591	3507.857	3290.571	3282.225	3306.319	3224.66	3223.755	3262.373	4628.729	3245.382	3436.138
	worst	3224.249	3639.124	3449.777	4124.025	3421.134	3869.463	3676.979	3250.099	3311.729	3362.006	5473.508	3341.919	3489.332
	std	9.925705	143.5802	92.80698	264.1424	61.78087	259.6228	157.9012	11.04206	38.67463	47.4773	413.4343	40.6175	22.19488
	median	3217.847	3368.977	3373.61	3807.959	3302.691	3642.581	3450.333	3234.148	3265.954	3327.287	5183.427	3297.81	3467.192
	rank	1	8	7	12	6	11	10	2	3	5	13	4	9
C17-F28	mean	3310.389	3608.074	3279.513	5904.207	3265.216	5091.362	3573.327	3237.176	3491.163	3607.88	3556.346	3350.173	3615.139
	best	3210.894	3491.04	3245.999	5568.396	3206.032	4122.444	3506.162	3218.46	3382.682	3493.443	3479.024	3251.203	3506.504
	worst	3398.371	3834.864	3314.364	6255.937	3373.749	6052.284	3629.175	3255.912	3763.179	3765.012	3718.62	3531.804	3678.771
	std	76.84487	160.5476	27.94958	328.4469	75.21557	889.448	63.20233	20.24324	181.9531	126.2162	109.5052	124.2419	75.08981
	median	3316.146	3553.196	3278.844	5896.247	3240.542	5095.36	3578.986	3237.165	3409.395	3586.532	3513.871	3308.842	3637.641
	rank	4	10	3	13	2	12	8	1	6	9	7	5	11
C17-F29	mean	3513.911	3928.315	4412.489	5807.492	3672.668	4787.271	5058.604	4072.709	3994.36	4518.153	5204.786	4232.107	4550.496
	best	3438.997	3649.853	4031.944	5122.188	3586.227	4310.399	4512.631	4002.885	3773.157	4421.967	4908.07	4047.214	4380.996
	worst	3601.815	4058.043	4644.465	6750.722	3787.399	5070.882	5647.867	4125.836	4324.094	4661.445	5483.886	4365.615	4714.488
	std	75.06742	187.7454	271.1545	799.9387	87.08749	350.1526	515.2403	54.79906	263.3728	108.3907	309.7752	137.1358	146.445
	median	3507.415	4002.683	4486.774	5678.529	3658.523	4883.901	5036.96	4081.058	3940.094	4494.599	5213.594	4257.798	4553.251
	rank	1	3	7	13	2	10	11	5	4	8	12	6	9
C17-F30	mean	8305.989	137,769.6	1,529,425	3.03 × 10^9^	9157.746	6.15 × 10^8^	30,762,556	3,049,986	9,525,378	25,150,289	2,426,811	104,576.7	987,308.6
	best	6809.307	59,525.81	539,075.8	2.18 × 10^9^	7017.166	7,706,794	5,608,686	1,699,158	1,885,661	19,520,550	2,118,389	8897.047	568,046.1
	worst	9524.921	183,375.5	2,708,640	3.35 × 10^9^	10,822.16	1.23 × 10^9^	54,147,488	5,439,819	14,684,335	35,666,572	2,919,985	321,178.6	1,356,063
	std	1134.907	56,915.13	907,077.7	5.7 × 10^8^	1704.62	6.99 × 10^8^	19,847,912	1,654,366	6,013,987	7,204,436	345,090	145,516.1	362,444.2
	median	8444.864	154,088.4	1,434,992	3.3 × 10^9^	9395.826	6.1 × 10^8^	31,647,026	2,530,484	10,765,759	22,707,017	2,334,435	44,115.67	1,012,563
	rank	1	4	6	13	2	12	11	8	9	10	7	3	5
Sum rank		44	183	192	366	57	324	307	122	167	248	245	165	219
Mean rank		1.517241	6.310345	6.62069	12.62069	1.965517	11.17241	10.58621	4.206897	5.758621	8.551724	8.448276	5.689655	7.551724
Total rank		1	6	7	13	2	12	11	3	5	10	9	4	8

**Table 4:** Optimization results of the CEC 2017 test suite (for the dimension d=50).

		SOA	WSO	AVOA	RSA	MPA	TSA	WOA	MVO	GWO	TLBO	GSA	PSO	GA
C17-F1	mean	151,889.6	2.57 × 10^10^	9,830,140	9.96 × 10^10^	5,506,826	5.41 × 10^10^	7.14 × 10^9^	4,797,753	9.99 × 10^9^	2.21 × 10^10^	1.82 × 10^10^	5.74 × 10^9^	8.1 × 10^9^
	best	115,387.6	1.88 × 10^10^	1,170,313	8.71 × 10^10^	3,290,149	3.98 × 10^10^	5.01 × 10^9^	3,351,115	5.9 × 10^9^	1.65 × 10^10^	1.45 × 10^10^	1.44 × 10^9^	6.57 × 10^9^
	worst	247,412.7	3.39 × 10^10^	26,012,940	1.09 × 10^11^	8,579,338	6.44 × 10^10^	1.13 × 10^10^	5,622,433	1.44 × 10^10^	3.09 × 10^10^	2.18 × 10^10^	1.05 × 10^10^	9.01 × 10^9^
	std	63,842.31	6.64 × 10^9^	11,038,046	9.48 × 10^9^	2,249,133	1.16 × 10^10^	2.83 × 10^9^	1,007,788	3.47 × 10^9^	6.74 × 10^9^	2.97 × 10^9^	4.87 × 10^9^	1.14 × 10^9^
	median	122,379.1	2.51 × 10^10^	6,068,653	1.01 × 10^11^	5,078,909	5.6 × 10^10^	6.13 × 10^9^	5,108,731	9.83 × 10^9^	2.05 × 10^10^	1.83 × 10^10^	5.54 × 10^9^	8.41 × 10^9^
	rank	1	11	4	13	3	12	6	2	8	10	9	5	7
C17-F3	mean	25,741.16	109,166.7	155,708.8	167,728.7	27,077.85	109,111	239,463.3	55,133.09	121,422.4	98,432.16	189,072.7	227,319.8	289,533.1
	best	17,748.75	87,516.26	119,637	152,152.9	18,689.86	93,934.99	149,929.1	35,708.23	109,107.7	82,388.28	170,742.6	169,886.5	206,512.9
	worst	31,049.77	131,269.6	189,453.3	182,838.5	32,278.65	143,029.3	381,283.7	88,723.05	137,403.7	10,7613.2	213,623.9	306,999.1	361,157.1
	std	5842.384	17,915.62	31,532.21	13,624.19	6102.045	22,781.45	99,541.9	24,999.8	12,061.24	11,298.13	20,728.75	57,433.93	67,162.39
	median	27,083.06	108,940.5	156,872.5	167,961.6	28,671.45	99,739.83	213,320.2	48,050.53	119,589.1	101,863.6	185,962.1	216,196.8	295,231.1
	rank	1	6	8	9	2	5	12	3	7	4	10	11	13
C17-F4	mean	556.8209	4628.003	711.7594	25,211.16	603.1969	9115.47	1767.649	592.487	1175.328	3505.154	3225.817	978.3787	1594.562
	best	537.4312	3095.048	692.7891	16,625.85	544.7605	7469.591	1392.045	533.3314	951.1455	2035.156	2692.306	831.7601	1584.14
	worst	600.4123	6512.632	744.1292	30,113.72	642.9742	11,041.53	2186.232	633.6151	1702.492	5193.195	3440.077	1063.683	1605.234
	std	29.38858	1751.262	22.72217	6158.629	43.34086	1468.831	333.7792	48.89051	353.3024	1632.081	357.6048	103.4355	10.48297
	median	544.72	4452.167	705.0597	27,052.53	612.5264	8975.38	1746.16	601.5007	1023.838	3396.133	3385.444	1009.036	1594.437
	rank	1	11	4	13	3	12	8	2	6	10	9	5	7
C17-F5	mean	677.7792	825.0179	879.2559	1166.95	721.1215	1151.616	1032.234	716.9972	732.7364	1062.166	824.3543	843.4191	930.9574
	best	656.4368	798.6558	847.0349	1146.761	700.2873	1006.991	966.5428	655.0431	654.3651	1050.273	768.6373	757.5923	874.5555
	worst	704.4044	854.3394	923.0372	1180.923	741.884	1243.544	1058.35	768.2161	842.6329	1085.351	863.1571	935.7099	986.5072
	std	23.359	23.67232	32.825	15.67259	16.98195	101.2598	43.90967	46.69174	80.43853	15.84562	44.50277	73.25487	46.17203
	median	675.1377	823.5383	873.4757	1170.059	721.1573	1177.965	1052.021	722.3649	716.9737	1056.52	832.8113	840.1871	931.3835
	rank	1	6	8	13	3	12	10	2	4	11	5	7	9
C17-F6	mean	612.5444	650.0934	663.6411	702.8348	636.3891	697.6476	697.9072	641.0802	624.8983	664.2849	661.2626	651.6176	648.1166
	best	608.3547	647.0002	658.5488	700.4264	610.8922	689.7925	695.4772	627.1604	621.0722	655.5853	656.0718	644.4713	637.0315
	worst	618.0937	652.6056	669.415	705.9343	658.9156	705.5216	701.5624	662.1438	634.2509	670.3405	664.3465	656.3579	653.6417
	std	4.065504	2.809484	5.020008	2.578301	21.6058	6.447668	2.764061	15.49878	6.284174	6.536734	3.64112	5.419492	7.510199
	median	611.8646	650.3839	663.3002	702.4892	637.8744	697.6381	697.2946	637.5083	622.1351	665.6069	662.3161	652.8207	650.8966
	rank	1	6	9	13	3	11	12	4	2	10	8	7	5
C17-F7	mean	1020.053	1634.936	1719.618	1959.322	1061.083	1830.724	1790.789	1104.083	1082.367	1452.483	1449.5	1226.391	1392.31
	best	984.9752	1274.275	1644.257	1873.413	1012.389	1488.422	1657.407	1042.23	977.2225	1415.913	1271.118	1090.999	1331.588
	worst	1069.875	1936.749	1789.939	2069.047	1096.42	2051.525	1928.988	1216.458	1156.252	1470.253	1586.373	1346.737	1447.051
	std	36.24558	272.8645	61.93246	83.88886	35.62031	252.3578	135.164	77.13688	75.41492	24.69756	142.0339	110.3465	48.90072
	median	1012.68	1664.36	1722.138	1947.414	1067.762	1891.475	1788.38	1078.822	1097.996	1461.884	1470.255	1233.914	1395.3
	rank	1	9	10	13	2	12	11	4	3	8	7	5	6
C17-F8	mean	992.8944	1119.935	1143.249	1485.684	1054.1	1501.057	1300.005	1093.326	1037.12	1343.492	1160.173	1090.87	1239.259
	best	958.4409	1089.39	1094.35	1452.256	984.2098	1375.148	1199.351	1031.358	993.6126	1294.575	1151.218	1064.359	1164.301
	worst	1018.161	1182.289	1193.203	1509.275	1144.524	1561.062	1386.4	1211.078	1054.829	1379.624	1175.097	1135.192	1276.922
	std	25.38413	43.54379	56.20376	23.96556	67.8529	87.41298	78.03448	81.14765	29.12162	38.09022	10.62307	31.92935	53.04527
	median	997.4879	1104.031	1142.721	1490.603	1043.832	1534.009	1307.133	1065.433	1050.02	1349.885	1157.188	1081.965	1257.907
	rank	1	6	7	12	3	13	10	5	2	11	8	4	9
C17-F9	mean	3379.968	31,574.54	13,994.58	38,384.66	3535.572	43,997.3	38,999.55	12,924.53	10,955.82	24,490.33	11,230.46	13,220.99	11,936.19
	best	2720.798	27,546.88	13,334.96	36,059.86	2795.909	34,228.79	27,771.85	4379.022	7695.713	17,011.15	10,226.78	8972.343	9776.976
	worst	4482.205	35,368.01	14,911.51	40,280.9	4766.806	51,238.8	57,115.19	20,429.49	13,132.49	27,535.96	12,132.64	21,544.05	14,035.6
	std	766.3366	4276.902	681.8704	1998.844	858.076	7407.689	12,869.39	8072.639	2466.082	5024.465	792.2221	5669.612	2264.86
	median	3158.435	31,691.64	13,865.93	38,598.94	3289.786	45,260.8	35,555.58	13,444.81	11,497.54	26,707.11	11,281.22	11,183.79	11,966.1
	rank	1	10	8	11	2	13	12	6	3	9	4	7	5
C17-F10	mean	6174.072	7403.704	8579.601	14,818.96	6667.021	13,370.03	12,530.29	7041.612	7036.968	14,639.16	8868.741	8635.025	11,353.66
	best	5757.895	6703.828	8018.602	14,572.31	5919.308	12,643.44	11,659.82	6312.651	6849.65	13,468.42	7973.388	7336.097	10,137.65
	worst	6627.51	7941.478	9205.324	15,177.46	7192.289	13,961.87	13,907.52	7530.854	7297.502	15,497.98	9980.347	9821.611	11,876.02
	std	461.6323	515.452	503.2166	286.0404	585.7901	662.7266	1020.996	557.1729	220.1985	848.7871	837.357	1033.431	825.7338
	median	6155.442	7484.756	8547.239	14,763.03	6778.245	13,437.41	12,276.92	7161.472	7000.36	14,795.11	8760.614	8691.196	11,700.49
	rank	1	5	6	13	2	11	10	4	3	12	8	7	9
C17-F11	mean	1334.96	4491.299	1635.402	22,214.14	1309.933	10,609.27	3565.628	1464.313	8372.954	5000.771	15,005.37	1892.931	15,726.03
	best	1281.171	3159.729	1507.538	19,751.85	1305.562	6841.722	3031.132	1362.16	4716.472	4243.983	14,069.52	1462.895	11,246.91
	worst	1372.22	6882.562	1798.581	24,081.65	1315.877	15,049.35	3780.766	1592.317	12,480.16	5708.219	17,016.79	2863.255	19,576.5
	std	38.83575	1664.8	134.2452	1809.233	4.400385	4211.171	358.468	112.5475	3546.225	655.6899	1355.471	655.2459	3430.853
	median	1343.224	3961.453	1617.745	22,511.53	1309.145	10,273	3725.307	1451.387	8147.593	5025.441	14,467.58	1622.787	16,040.36
	rank	2	7	4	13	1	10	6	3	9	8	11	5	12
C17-F12	mean	11,251,731	5.27 × 10^9^	78,049,621	7.57 × 10^10^	12,693,027	1.72 × 10^10^	1.61 × 10^9^	98,207,747	1.57 × 10^9^	3.95 × 10^9^	2.31 × 10^9^	1.16 × 10^9^	2.07 × 10^8^
	best	10,038,799	1.65 × 10^9^	33,060,130	5.52 × 10^10^	9,956,212	7.73 × 10^9^	8.92 × 10^8^	63,359,996	3.8 × 10^8^	2.82 × 10^9^	7.6 × 10^8^	2.74 × 10^8^	96,807,585
	worst	12,129,544	9.38 × 10^9^	1.21 × 10^8^	1.04 × 10^11^	18,145,198	2.85 × 10^10^	2.96 × 10^9^	1.62 × 10^8^	2.93 × 10^9^	5.13 × 10^9^	4.15 × 10^9^	1.74 × 10^9^	3.39 × 10^8^
	std	1,025,491	3.48 × 10^9^	46,894,592	2.24 × 10^10^	3,729,890	8.96 × 10^9^	9.5 × 10^8^	46,032,952	1.37 × 10^9^	9.8 × 10^8^	1.4 × 10^9^	6.93 × 10^8^	1.02 × 10^8^
	median	11,419,290	5.02 × 10^9^	79,267,889	7.19 × 10^10^	11,335,349	1.62 × 10^10^	1.3 × 10^9^	83,612,325	1.48 × 10^9^	3.93 × 10^9^	2.16 × 10^9^	1.32 × 10^9^	1.95 × 10^8^
	rank	1	11	3	13	2	12	8	4	7	10	9	6	5
C17-F13	mean	25,194.61	7.45 × 10^8^	158,443	4.58 × 10^10^	29,207.77	1.23 × 10^10^	1.55 × 10^8^	169,862.1	2.14 × 10^8^	6.76 × 10^8^	19,712,046	2.11 × 10^8^	26,307,383
	best	14,947.67	39,849,034	36,363.7	2.32 × 10^10^	14,829.62	5.77 × 10^9^	72,249,484	89,730.53	1.19 × 10^8^	4.77 × 10^8^	33,125.95	60217.58	6,804,282
	worst	35,197.79	2.32 × 10^9^	349,245.4	6.59 × 10^10^	49,867.22	1.62 × 10^10^	2.35 × 10^8^	250,596.3	3.63 × 10^8^	7.89 × 10^8^	66,446,165	5.9 × 10^8^	43,575,192
	std	10,557.9	1.08 × 10^9^	133,793.4	1.79 × 10^10^	16,453.73	4.55 × 10^9^	67,992,278	65,749.65	1.14 × 10^8^	1.41 × 10^8^	31,690,714	2.77 × 10^8^	15,084,746
	median	25,316.49	3.12 × 10^8^	124,081.5	4.71 × 10^10^	26,067.12	1.36 × 10^10^	1.56 × 10^8^	169,560.9	1.88 × 10^8^	7.19 × 10^8^	6184,447	1.28 × 10^8^	27,425,029
	rank	1	11	3	13	2	12	7	4	9	10	5	8	6
C17-F14	mean	1561.629	954,429.1	1301,714	51,521,240	1644.844	11,042,571	6537,253	338,362	2,186,341	1,214,913	16,125,876	177,015.6	11,328,374
	best	1551.57	40,522.92	403,134.7	15,801,584	1610.492	744,015.3	1234,192	193,899.1	117,686.7	1,015,556	3,656,077	54,705.12	8,041,233
	worst	1569.011	2,245,963	3,100,586	1.04 × 10^8^	1665.859	38,689,467	18,106,774	480,695.4	4,353,688	1,446,289	26,477,284	410,064.8	15,650,354
	std	7.48164	929,949.3	1226,148	37,650,671	24.55157	18,475,034	7,877,558	121,825.3	2,228,784	193,566.3	10,346,083	15,8781.1	3165,277
	median	1562.968	765,615.1	851,568.7	42,984,409	1651.514	2,368,402	3,404,022	339,426.6	2,136,996	1,198,904	17,185,072	121,646.3	10,810,955
	rank	1	5	7	13	2	10	9	4	8	6	12	3	11
C17-F15	mean	2088.202	84,644.13	40,352.97	4.46 × 10^9^	2168.064	2.15 × 10^9^	45,457,765	106,616	23,648,137	50,130,625	2.1 × 10^8^	20,135.54	13,007,299
	best	2001.116	19,632.75	24,871.69	3.48 × 10^9^	2081.998	7332003	12,695,487	61,228.51	114,896.2	24,391,486	20291.99	2938.625	1,429,335
	worst	2202.268	176,250.9	74,394	5.28 × 10^9^	2262.088	6.23 × 10^9^	86,925,294	163,102.2	42,481,430	77,779,135	8.16 × 10^8^	40,169.41	25,370,774
	std	84.61488	66,115.44	22,955.17	7.97 × 10^8^	77.26539	2.81 × 10^9^	35,216,059	42,226.99	18,140,241	25,125,637	4.04 × 10^8^	17,100.37	9,825,680
	median	2074.712	71,346.42	31,073.09	4.53 × 10^9^	2164.086	1.19 × 10^9^	41,105,139	101,066.6	25,998,110	49,175,940	12,713,833	18,717.06	12,614,543
	rank	1	5	4	13	2	12	9	6	8	10	11	3	7
C17-F16	mean	2737.231	3413.809	4503.424	7943.425	2910.262	5681.85	6366.838	3202.22	3082.343	5298.307	4079.109	3392.654	3966.724
	best	2549.162	2716.472	4180.052	5899.312	2532.098	4684.45	5729.784	2828.437	2728.369	5156.685	3701.751	2883.776	3447.318
	worst	2910.818	3865.438	4935.667	11,948.51	3329.982	6106.082	6736.105	3693.078	3311.269	5434.487	4494.833	3690.971	4613.582
	std	148.0198	530.6386	363.5425	2755.255	326.6354	668.1979	465.9646	390.8859	248.6798	114.3378	375.0628	374.9784	542.6677
	median	2744.471	3536.663	4448.988	6962.941	2889.485	5968.433	6500.731	3143.683	3144.867	5301.028	4059.925	3497.935	3902.998
	rank	1	6	9	13	2	11	12	4	3	10	8	5	7
C17-F17	mean	2573.195	3002.378	3658.258	11,639.65	2700.396	5430.256	4790.562	3274.99	2870.234	3934.082	3937.182	3256.484	3396.164
	best	2411.363	2677.716	3193.284	8466.86	2459.043	3655.951	3430.344	2935.277	2516.354	3671.376	3461.219	3075.895	3257.524
	worst	2687.187	3690.493	4212.583	15,174.64	2833.242	7076.82	5593.094	3534.417	3082.95	4167.482	4255.079	3551.107	3543.127
	std	115.9525	464.2714	492.9618	2763.849	165.2191	1442.127	967.311	265.5602	264.1476	206.5056	348.598	224.8963	117.1755
	median	2597.115	2820.652	3613.583	11,458.55	2754.65	5494.126	5069.405	3315.133	2940.815	3948.735	4016.215	3199.467	3392.002
	rank	1	4	8	13	2	12	11	6	3	9	10	5	7
C17-F18	mean	14,578.01	2,805,300	2,568,723	1.2 × 10^8^	15,143.98	45,107,782	61,393,853	2,423,534	21,017,655	8731,271	8,959,584	832,165.9	16,730,316
	best	4589.064	379,650.7	332,655.2	53,792,821	4782.991	5,112,949	12,472,675	1,316,487	3,654,688	2,959,680	4,236,144	500,424.3	7,543,771
	worst	30,709.1	5,279,270	4,705,067	1.66 × 10^8^	32,192.73	1.12 × 10^8^	1.43 × 10^8^	3,480,084	64,837,312	21,724,952	16,743,556	1,208,421	21,978,246
	std	11,718.82	2,146,247	2,215,520	55,161,299	12,301.32	47,374,611	61,960,905	1,015,362	29,414,178	8,763,297	5,701,618	292,071.8	6,671,844
	median	11,506.93	2,781,141	2,618,585	1.29 × 10^8^	11,800.09	31,796,854	44,854,458	2,448,782	7,789,311	5,120,226	7,429,317	809,909.1	18,699,624
	rank	1	6	5	13	2	11	12	4	10	7	8	3	9
C17-F19	mean	2108.057	159,032.8	276,582.5	4.09 × 10^9^	2212.473	1.16 × 10^9^	3,880,347	5,795,751	11,522,364	69,314,944	481,438.7	27,395.04	852,195.6
	best	2041.296	36,866.49	96,991.5	2.76 × 10^9^	2118.389	10389370	424,737.4	1,799,228	2,465,418	29,753,913	276,793.4	6758.912	657,534.7
	worst	2184.699	419,582.6	570,566.3	5.06 × 10^9^	2328.246	2.36 × 10^9^	7,483,304	9,042,447	32,549,618	1.43 × 10^8^	1,055,175	78,011.97	1,075,172
	std	77.04148	178,299	205,522.6	1.02 × 10^9^	108.4181	1.32 × 10^9^	3,181,528	3,059,230	14,306,749	52,264,278	382,629.3	33,896.96	171,296
	median	2103.117	89,841.03	219,386.1	4.27 × 10^9^	2201.628	1.14 × 10^9^	3,806,673	6,170,664	5,537,210	52,379,219	296,893.2	12,404.65	838,037.7
	rank	1	4	5	13	2	12	8	9	10	11	6	3	7
C17-F20	mean	2547.209	3273.289	3337.748	4223.731	2931.538	3847.953	4145.357	3157.849	3220.128	3846.377	4165.34	3170.407	3108.672
	best	2498.065	2844.233	2688.235	3927.478	2786.566	3412.071	3572.722	2814.451	2952.323	3618.013	3846.16	2963.648	3017.065
	worst	2648.126	3919.758	3912.457	4391.213	3205.982	4148.526	4368.731	3500.378	3597.963	4078.028	4468.586	3305.249	3201.274
	std	68.60929	507.0025	524.2096	204.2398	187.4237	312.5293	382.4528	282.9635	276.6289	252.1705	255.2723	155.2787	92.03002
	median	2521.322	3164.582	3375.15	4288.117	2866.802	3915.608	4319.989	3158.283	3165.113	3844.734	4173.307	3206.364	3108.174
	rank	1	7	8	13	2	10	11	4	6	9	12	5	3
C17-F21	mean	2461.015	2750.03	2785.955	3080.695	2442.558	2978.155	3074.598	2538.401	2541.013	2858.889	2878.604	2691.435	2775.299
	best	2454.062	2655.45	2655.912	2968.624	2438.788	2961.782	2916.837	2465.581	2500.98	2816.416	2802.947	2602.644	2699.723
	worst	2467.75	2890.739	2985.839	3173.847	2445.937	3018.498	3252.448	2618.492	2568.759	2900.466	2921.082	2738.388	2838.696
	std	5.753449	100.6273	142.2127	97.29591	3.785764	26.98508	139.2031	75.69416	28.66691	35.38466	53.10801	60.48597	63.22481
	median	2461.123	2726.966	2751.035	3090.154	2442.753	2966.17	3064.553	2534.767	2547.157	2859.337	2895.194	2712.354	2781.388
	rank	2	6	8	13	1	11	12	3	4	9	10	5	7
C17-F22	mean	5068.179	10,601.63	11,695.73	17,156.72	5289.649	14,744.41	14,773.48	9918.28	8391.527	16,328.37	12,006.95	11,379.86	11,852.76
	best	2317.659	9792.013	9254.48	16,976.74	2386.837	13,539.03	12,269.29	9328.101	6397.783	15,366.03	11,736.64	9262.03	5080.869
	worst	8010.131	11,189.41	13,600.29	17,419.31	8618.209	15,424.94	16,718.84	10,360.01	10,211.59	17,081.38	12,207.05	13,321.13	15,030.32
	std	3175.556	587.4619	1976.242	202.7537	3338.394	827.801	1899.776	440.2749	1597.655	721.302	208.2034	1660.84	4566.475
	median	4972.464	10,712.55	11,964.08	17,115.42	5076.776	15,006.83	15052.9	9992.504	8478.367	16,433.04	12,042.05	11,468.14	13,649.93
	rank	1	5	7	13	2	10	11	4	3	12	9	6	8
C17-F23	mean	2879.864	3742.803	3333.282	3984.37	3013.484	4203.96	3625.626	2987.978	3030.149	3353.519	4909.23	3393.538	3411.219
	best	2819.095	3528.564	3242.024	3931.858	2911.823	3785.87	3524.337	2952.645	2998.021	3281.372	4698.938	3325.579	3287.366
	worst	2929.666	3941.54	3420.534	4030.28	3057.88	4675.038	3725.834	3017.048	3058.198	3472.351	5093.602	3455.819	3511.652
	std	45.73851	215.66	84.27723	41.04961	68.2742	415.8831	87.17086	27.56366	26.83748	82.51679	161.8938	53.58173	114.0835
	median	2885.347	3750.553	3335.286	3987.67	3042.116	4177.466	3626.167	2991.11	3032.189	3330.177	4922.189	3396.376	3422.929
	rank	1	10	5	11	3	12	9	2	4	6	13	7	8
C17-F24	mean	3083.504	4142.676	3560.758	4611.799	3081.981	4037.358	3901.95	3096.846	3208.072	3525.045	4499.5	3660.766	3832.45
	best	3015.965	3876.67	3440.367	4085.627	3000.511	3762.786	3823.238	3056.288	3106.145	3473.443	4461.401	3604.282	3679.231
	worst	3132.978	4662.206	3761.438	5903.316	3136.003	4367.077	3988.185	3126.944	3377.68	3567.211	4558.036	3744.37	4008.357
	std	51.40778	355.0229	139.0862	869.707	58.68838	279.9751	68.7295	29.97032	117.5406	46.10856	44.92477	64.59743	155.6822
	median	3092.536	4015.914	3520.614	4229.127	3095.705	4009.785	3898.188	3102.076	3174.232	3529.763	4489.28	3647.206	3821.106
	rank	2	11	6	13	1	10	9	3	4	5	12	7	8
C17-F25	mean	3068.733	4544.506	3193.318	12,627.24	3087.808	6295.735	4867.662	3090.15	3831.696	4514.777	4376.108	3145.639	4421.074
	best	3046.855	3802.561	3163.265	10,093.24	3054.996	5817.769	4493.166	3057.295	3605.51	3911.35	4001.137	3096.16	4179.04
	worst	3077.521	5346.191	3243.541	14,183.95	3131.849	6738.606	5247.977	3113.089	4055.253	4952.488	5087.889	3185.549	4659.918
	std	14.64504	632.2521	34.79092	1922.174	33.4426	392.6728	344.0079	23.71	233.4356	519.0428	511.0707	39.00329	196.3794
	median	3075.279	4514.635	3183.232	13,115.89	3082.194	6313.284	4864.752	3095.108	3833.011	4597.634	4207.703	3150.422	4422.67
	rank	1	10	5	13	2	12	11	3	6	9	7	4	8
C17-F26	mean	4455.594	10,887.89	11,546.28	15,965.14	4869.194	14,453.46	15,596.31	6182.373	7275.879	9760.462	12,162.55	7406.202	7234.428
	best	3501.58	6702.71	10,993.2	15,295.97	3470.604	13,342.37	14,726.99	5832.205	6155.413	9467.029	11,786.48	3655.993	6898.45
	worst	6191.431	13,062.05	12,099.17	16,992.01	6448.084	15,734.17	16,328.64	6713.88	8916.753	10,118.65	12,597.7	9175.091	7781.243
	std	1226.994	2918.176	452.4794	735.8058	1422.756	985.4345	658.776	386.3427	1291.61	270.3168	338.5611	2584.358	392.6468
	median	4064.682	11,893.4	11,546.38	15,786.29	4779.045	14,368.66	15,664.8	6091.705	7015.674	9728.086	12,133.01	8396.863	7129.01
	rank	1	8	9	13	2	11	12	3	5	7	10	6	4
C17-F27	mean	3363.189	4366.514	3897.689	5125.112	3448.795	5237.028	4903.756	3470.777	3717.304	3693.737	8470.607	3854.516	4433.001
	best	3320.606	4286.133	3848.523	4720.746	3366.621	4761.12	4426.306	3432.261	3636.637	3635.281	8197.85	3698.911	4182.635
	worst	3430.944	4491.872	3970.348	5408.718	3607.609	5915.32	5499.852	3571.634	3830.143	3756.612	8860.917	4102.243	4668.505
	std	49.36455	94.88507	54.71087	330.3539	110.5841	485.8052	449.642	67.53284	87.25992	55.50564	322.7935	173.0623	198.6077
	median	3350.604	4344.025	3885.944	5185.491	3410.475	5135.835	4844.433	3439.606	3701.218	3691.528	8411.831	3808.456	4440.433
	rank	1	8	7	11	2	12	10	3	5	4	13	6	9
C17-F28	mean	3304.026	5927.069	3619.749	11,797.14	3373.545	6674.615	5136.264	3310.385	4426.418	5582.872	5201.752	3869.901	4966.713
	best	3279.151	5470.669	3530.722	10,414.72	3341.683	5799.475	4614.257	3301.588	4091.054	4696.446	5136.095	3456.669	4889.397
	worst	3330.152	6322.616	3716.895	15,468.78	3429.377	8088.887	5807.894	3314.652	4863.695	6048.066	5331.294	4264.888	5054.661
	std	21.30892	374.6972	91.86549	2452.27	38.51731	1032.791	510.3959	6.127407	361.6129	603.6288	88.70492	382.4002	75.04482
	median	3303.4	5957.496	3615.689	10,652.52	3361.561	6405.05	5061.453	3312.649	4375.462	5793.489	5169.809	3879.023	4961.397
	rank	1	11	4	13	3	12	8	2	6	10	9	5	7
C17-F29	mean	4311.503	5508.011	5680.663	20,771.09	4536.4	8098.192	8442.254	5166.941	4755.15	6354.818	8566.63	5084.367	6583.69
	best	4038.342	4914.424	5522.082	10,858.34	4288.411	6715.261	6101.824	4992.053	4634.581	6001.988	7009.127	4698.529	6508.721
	worst	4509.912	6458.317	5834.624	33,002.78	4775.32	10,262.49	10,228.64	5303.74	4937.432	6842.113	11,314.84	5645.839	6630.906
	std	198.4451	720.6455	127.7043	9862.853	200.54	1638.244	1783.724	129.1374	137.3209	361.7188	1938.806	463.4686	52.64746
	median	4348.878	5329.651	5682.974	19,611.63	4540.934	7707.507	8719.274	5185.984	4724.293	6287.585	7971.274	4996.55	6597.567
	rank	1	6	7	13	2	10	11	5	3	8	12	4	9
C17-F30	mean	2,025,569	35,167,304	23,274,855	5.86 × 10^9^	2,682,820	1.04 × 10^9^	2.59 × 10^8^	80,472,757	1.24 × 10^8^	2.86 × 10^8^	1.97 × 10^8^	5990,562	58,956,549
	best	1,181,088	23,290,188	14,200,523	3.6 × 10^9^	1,328,294	1.77 × 10^8^	98,570,183	51,907,046	73641400	1.95 × 10^8^	1.51 × 10^8^	1211,278	45,838,053
	worst	2,875,328	42,761,447	31,913,446	9.19 × 10^9^	3,372,211	2.89 × 10^9^	4.62 × 10^8^	1.34 × 10^8^	1.7 × 10^8^	5.03 × 10^8^	2.58 × 10^8^	18,526,499	66,979,535
	std	698,314.3	8,325,550	8701,063	2.41 × 10^9^	918,657.5	1.26 × 10^9^	1.52 × 10^8^	37,734,532	41,204,160	1.47 × 10^8^	449,807,88	8,368,296	9,118,859
	median	2,022,930	37,308,790	23,492,726	5.32 × 10^9^	3,015,388	5.51 × 10^8^	2.37 × 10^8^	68,043,751	1.27 × 10^8^	2.22 × 10^8^	1.89 × 10^8^	2,112,235	61,504,304
	rank	1	5	4	13	2	12	10	7	8	11	9	3	6
Sum rank		32	216	182	366	62	325	287	115	159	256	264	157	218
Mean rank		1.103448	7.448276	6.275862	12.62069	2.137931	11.2069	9.896552	3.965517	5.482759	8.827586	9.103448	5.413793	7.517241
Total rank		1	7	6	13	2	12	11	3	5	9	10	4	8

**Table 5:** Optimization results of the CEC 2017 test suite (for the dimension d=100).

		SOA	WSO	AVOA	RSA	MPA	TSA	WOA	MVO	GWO	TLBO	GSA	PSO	GA
C17-F1	mean	160,265.1	1.19 × 10^11^	4.08 × 10^9^	2.48 × 10^11^	5 × 10^8^	1.31 × 10^11^	6.53 × 10^10^	67,475,871	5.52 × 10^10^	9.46 × 10^10^	1.4 × 10^11^	2.14 × 10^10^	6.99 × 10^10^
	best	138,023.5	1.11 × 10^11^	1.98 × 10^9^	2.44 × 10^11^	3.71 × 10^8^	1.18 × 10^11^	6.11 × 10^10^	57,348,883	3.89 × 10^10^	7.98 × 10^10^	1.18 × 10^11^	1.65 × 10^10^	6.09 × 10^10^
	worst	207,953.1	1.25 × 10^11^	5.86 × 10^9^	2.5 × 10^11^	7.33 × 10^8^	1.47 × 10^11^	6.77 × 10^10^	77,330,536	6.37 × 10^10^	1.12 × 10^11^	1.58 × 10^11^	2.72 × 10^10^	7.63 × 10^10^
	std	32,666.58	5.84 × 10^9^	1.59 × 10^9^	2.86 × 10^9^	1.6 × 10^8^	1.44 × 10^10^	2.97 × 10^9^	10,406,022	1.16 × 10^10^	1.35 × 10^10^	1.71 × 10^10^	4.42 × 10^9^	6.58 × 10^9^
	median	147,541.9	1.2 × 10^11^	4.23 × 10^9^	2.49 × 10^11^	4.47 × 10^8^	1.3 × 10^11^	6.61 × 10^10^	67,612,033	5.91 × 10^10^	9.31 × 10^10^	1.42 × 10^11^	2.1 × 10^10^	7.13 × 10^10^
	rank	1	10	4	13	3	11	7	2	6	9	12	5	8
C17-F3	mean	155,460.5	284,175.9	347,477.9	343,395.2	163,200.2	375,331.1	977,486.3	500,947.0	389,167.5	329,791.4	365,707.5	607,827.5	654,898.7
	best	136,109.2	248,717.4	339,354.5	331,243.9	142,444.6	312,194.3	943,418.6	444,428.5	374,416.5	301,764.9	354,759.5	419,980.8	516,193.8
	worst	164,612.9	309,081.9	355,262.9	350,541.3	173,294.4	415,302.3	1,042,632.0	632,522.5	415,170.4	355,744.3	377,857.9	772,544	754,873.2
	std	13,110.98	28,313.85	6723.431	9036.029	14,518.07	44,174.85	46,210.51	88,310.45	17,980.22	29,865.05	9617.273	14,9726.7	116,056.6
	median	160,559.9	289,452.2	347,647.2	345,897.8	168,530.8	386,913.8	961,947.5	463,418.4	383,541.7	330,828.1	365,106.3	619,392.6	674,264
	rank	1	3	6	5	2	8	13	10	9	4	7	11	12
C17-F4	mean	707.8421	19,140.58	1615.548	79,883.06	1078.93	19,621.12	13,056.76	745.6667	5449.769	9178.908	36,332.13	3066.08	9469.057
	best	655.7759	14,643.47	1348.811	72,405.03	1013.777	14,895.87	10,390.22	702.4754	3735.491	6841.936	25,952.88	2030.326	9196.622
	worst	793.6724	21,181.19	1785.136	89,013.13	1187.742	28,200.33	14,,767.67	773.1848	7492.231	10,520.72	45,851.15	4909.819	9906.689
	std	60.21498	3087.898	197.5832	6886.744	75.37681	6081.237	1906.107	31.28424	1791.353	1681.483	8202.682	1313.62	306.1485
	median	690.96	20,368.84	1664.122	79,057.04	1057.1	17,694.15	13,534.58	753.5032	5285.676	9676.486	36,762.24	2662.088	9386.458
	rank	1	10	4	13	3	11	9	2	6	7	12	5	8
C17-F5	mean	1140.386	1416.959	1337.534	2014.712	1240.094	2115.385	1999.848	1237.633	1155.592	1860.505	1347.479	1399.646	1716.961
	best	1078.186	1389.332	1325.12	1977.422	1195.626	2036.245	1909.846	1113.65	1084.952	1792.429	1271.784	1348.014	1657.313
	worst	1174.568	1466.582	1347.384	2051.417	1308.247	2283.080	2096.681	1333.125	1222.116	1994.053	1469.717	1467.549	1744.248
	std	42.78946	34.79746	9.317251	37.35075	49.91629	113	76.57659	97.18324	56.28314	90.68752	86.24644	53.25039	40.1947
	median	1154.395	1405.962	1338.816	2015.004	1228.252	2071.107	1996.433	1251.878	1157.651	1827.769	1324.208	1391.51	1733.142
	rank	1	8	5	12	4	13	11	3	2	10	6	7	9
C17-F6	mean	640.8564	671.2647	664.0195	708.4028	635.4878	720.0932	702.7487	677.6025	641.7199	687.1312	668.1679	665.195	667.7512
	best	638.4984	665.4193	659.8006	703.2776	632.7878	708.063	692.7168	661.6266	636.6993	680.3454	666.4344	661.4675	662.3638
	worst	645.8414	687.6795	668.4418	711.4416	640.1923	730.9568	716.0015	693.8966	645.3746	696.9215	669.6235	670.454	674.3769
	std	3.412407	10.94671	3.561852	3.608402	3.400557	10.07273	10.16945	13.63377	3.924944	7.304814	1.69535	4.126496	5.003959
	median	639.5429	665.98	663.9178	709.446	634.4854	720.6764	701.1382	677.4434	642.4029	685.629	668.3068	664.4292	667.132
	rank	2	8	4	12	1	13	11	9	3	10	7	5	6
C17-F7	mean	1893.552	3374.351	3157.709	3828.487	2006.673	3664.688	3488.485	2248.066	2145.731	2970.465	2998.831	2589.66	2744.412
	best	1757.932	3209.741	2991.056	3733.397	1831.281	3130.538	3237.059	2030.148	2005.574	2855.161	2786.07	2157.752	2606.048
	worst	2019.125	3753.746	3297.533	3909.372	2119.133	4341.917	3644.585	2476.499	2342.976	3102.649	3161.247	2829.485	2825.746
	std	114.7911	257.1896	153.159	75.52237	124.2497	501.5494	182.1772	189.4768	147.1537	121.8524	178.6652	315.2845	100.1396
	median	1898.576	3266.959	3171.124	3835.589	2038.139	3593.148	3536.148	2242.807	2117.187	2962.026	3024.004	2685.703	2772.926
	rank	1	10	9	13	2	12	11	4	3	7	8	5	6
C17-F8	mean	1431.669	1802.604	1766.254	2528.82	1483.671	2517.501	2210.127	1482.502	1540.977	2240.139	1883.447	1919.521	2069.916
	best	1288.825	1707.765	1707.542	2502.557	1317.806	2416.13	2128.762	1356.216	1467.944	2124.134	1857.322	1747.085	2014.745
	worst	1505.145	1846.735	1795.721	2544.961	1637.492	2615.076	2313.552	1578.711	1603.175	2340.597	1932.538	2074.104	2101.56
	std	97.56967	63.86616	40.49818	18.33389	131.5389	83.6966	79.91965	97.93548	56.07926	95.53038	35.38708	164.7306	39.18582
	median	1466.353	1827.959	1780.877	2533.881	1489.693	2519.399	2199.096	1497.541	1546.395	2247.912	1871.965	1928.448	2081.68
	rank	1	6	5	13	3	12	10	2	4	11	7	8	9
C17-F9	mean	19,364.46	82,669.58	27,172.11	79,373.17	22,818.43	137,756.3	59,269.65	68,461.86	45,236.99	69,329.59	26,731.7	30,740.75	48,051.43
	best	16,807.53	75,191.45	22,605.98	76,723.33	17,418.05	108,475.1	45,027.85	49,383.76	33,072.81	65,899.36	24,236.3	25,972.61	45,151.63
	worst	21,107.66	91,644.35	30,594.59	81,543.18	25,883.72	152,399.4	83,531.69	94,754.6	54,921.68	73,999.44	30,955.57	36,306.87	53,358.57
	std	2103.88	7174.092	3328.994	2100.287	3868.815	19,968.74	17,085.29	19,622.14	9095.293	3435.528	2919.456	5374.775	3799.396
	median	19,771.33	81,921.27	27,743.93	79,613.09	23,985.98	145,075.3	54,259.52	64,854.53	46,476.73	68,709.78	25,867.47	30,341.75	46,847.75
	rank	1	12	4	11	2	13	8	9	6	10	3	5	7
C17-F10	mean	12,718.41	25,000.53	16,130.00	32,371.07	14,957.25	29,288.9	27,809.40	16,429.39	20,918.75	32,258.28	16,555.1	17,941.46	26,975.78
	best	12,215.97	17,070.00	13,311.48	31,423.28	13,758.01	28,082.1	25,632.71	12,929.29	16,553.94	31,909.45	15,209.4	16,127.12	26,382.45
	worst	13,595.73	31,663.57	18,484.04	32,801.76	15,544.55	30,431.1	29,064.27	20,010.88	32,186.21	32,592.30	17,295.4	20,010.14	27,903.87
	std	603.51	7406.58	2244.45	647.84	825.34	969.3	1538.77	2909.97	7526.20	360.10	923.7	1735.06	702.90
	median	12,530.97	25,634.28	16,362.23	32,629.62	15,263.23	29,321.1	28,270.31	16,388.69	17,467.43	32,265.69	16,857.76	17,814.29	26,808.41
	rank	1	8	3	13	2	11	10	4	7	12	5	6	9
C17-F11	mean	2576.639	66,539.98	66,864.48	215,441.7	4466.554	74,278.42	233,670.5	5121.713	73,277.27	54,827.31	182,410.7	80,069.76	163,604.5
	best	2351.245	48,807.46	60,071.57	164,806.4	3731.435	57,060.97	147,832.1	4503.97	59,898.28	49,308.24	150,790.3	50,850.22	122,067.2
	worst	2838.85	92,451.67	79,917.87	307,014.4	5502.63	83,711.11	404,045.3	6212.619	87,020.96	63,160.31	215,076.3	97,950.76	207,411.1
	std	224.0385	19,508.04	9184.966	64,071.68	763.2635	11,773.96	115,695.8	775.776	11,363.38	5914.53	27,214.48	22,176.82	41,642.89
	median	2558.232	62450.4	63,734.24	194,972.9	4316.076	78,170.81	191,402.3	4885.131	73,094.91	53,420.35	181,888.2	85,739.03	162,469.8
	rank	1	5	6	12	2	8	13	3	7	4	11	9	10
C17-F12	mean	21,615,544	5.33 × 10^10^	6.87 × 10^8^	1.79 × 10^11^	2.66 × 10^8^	6.03 × 10^10^	1.25 × 10^10^	5.51 × 10^8^	1.78 × 10^10^	2.51 × 10^10^	6.17 × 10^10^	7.41 × 10^9^	1.34 × 10^10^
	best	10,630,715	4.58 × 10^10^	3.65 ×10^8^	1.34 × 10^11^	1.03 × 10^8^	4.46 × 10^10^	9.28 × 10^9^	2.54 × 10^8^	1.19 × 10^10^	1.69 × 10^10^	4.86 × 10^10^	2.54 × 10^9^	1.17 × 10^10^
	worst	32,253,244	6.1 × 10^10^	1.1 × 10^9^	2.08 × 10^11^	3.55 × 10^8^	8.39 × 10^10^	1.46 × 10^10^	9.23 × 10^8^	3.18 × 10^10^	3.8 × 10^10^	6.66 × 10^10^	1.75 × 10^10^	1.58 × 10^10^
	std	9525,992	6.22 × 10^9^	3.16 × 10^8^	3.4 × 10^10^	1.13 × 10^8^	1.72 × 10^10^	2.28 × 10^9^	2.95 × 10^8^	9.38 × 10^9^	9.05 × 10^9^	8.78 × 10^9^	6.81 × 10^9^	2.01 × 10^9^
	median	21,789,109	5.33 × 10^10^	6.44 × 10^8^	1.87 × 10^11^	3.04 × 10^8^	5.64 × 10^10^	1.31 × 10^10^	5.13 × 10^8^	1.38 × 10^10^	2.28 × 10^10^	6.59 × 10^10^	4.8 × 10^9^	1.3 × 10^10^
	rank	1	10	4	13	2	11	6	3	8	9	12	5	7
C17-F13	mean	44,203.5	7.73 × 10^9^	102,552.1	4.46 × 10^10^	70,549.51	2.06 × 10^10^	8.69 × 10^8^	440,253.3	1.66 × 10^9^	4.43 × 10^9^	9.98 × 10^9^	1.63 × 10^8^	1.74 × 10^8^
	best	39,417.16	2.91 × 10^9^	72,417.29	3.45 × 10^10^	39,047.23	1.37 × 10^10^	4.27 × 10^8^	385,351.8	3490,814	1.73 × 10^9^	8.96 × 10^9^	63,314.68	61,879,640
	worst	47,640.75	1.2 × 10^10^	139,843.7	5.05 × 10^10^	89,174.52	3.1 × 10^10^	1.71 × 10^9^	549,081.5	5.13 × 10^9^	7.98 × 10^9^	1.11 × 10^10^	5.76 × 10^8^	2.79 × 10^8^
	std	3447.109	4.32 × 10^9^	28,590.46	7.42 × 10^9^	23,479.33	7.5 × 10^9^	5.8 × 10^8^	73,870.14	2.35 × 10^9^	2.61 × 10^9^	9.87 × 10^8^	2.76 × 10^8^	92177490
	median	44,878.03	8.02 × 10^9^	98,973.74	4.67 × 10^10^	76,988.15	1.88 × 10^10^	6.72 × 10^8^	413,289.9	7.45 × 10^8^	4.01 × 10^9^	9.92 × 10^9^	38354572	1.78 × 10^8^
	rank	1	10	3	13	2	12	7	4	8	9	11	5	6
C17-F14	mean	38,247.02	8,398,313	6,983,904	83,370,942	37,992.77	7574,523	23,051,859	2,406,385	7,632,300	12,005,340	8,966,839	3,232,258	16,151,979
	best	19,691.01	5,689,698	4,235,073	76,038,636	19,526.17	3959,310	16,617,918	1,206,786	4,435,632	8,814,373	6,670,450	720,653	10,615,559
	worst	70,208.67	11,219,705	11,593,673	91,264,970	69,787.65	15,774,376	35,953,797	4,757,866	13,293,148	15,067,242	14,374,476	7,262,728	21,599,852
	std	22,041.78	2,526,169	3,241,606	7,314,111	21,928.96	5,505,061	8787,473	1,618,096	3,949,408	2,628,175	3,652,301	2,811,928	4561,649
	median	31,544.2	8,341,924	6,053,435	83,090,081	31,328.63	5,282,204	19,817,861	1,830,443	6,400,211	12,069,874	7,411,215	2,472,826	16,196,252
	rank	2	8	5	13	1	6	12	3	7	10	9	4	11
C17-F15	mean	33,097.94	4.19 × 10^8^	88,181.73	2.46 × 10^10^	34,635.7	1.03 × 10^10^	1.95 × 10^8^	145,183.4	2.72 × 10^8^	7.94 × 10^8^	1.72 × 10^9^	1.18 × 10^9^	9,645,827
	best	16,382.21	23,910,679	72,089.62	1.76 × 10^10^	17,471.38	6.25 × 10^9^	69,888,989	80,674.01	1.24 × 10^8^	4.89 × 10^8^	5.36 × 10^8^	58117.71	6,699,332
	worst	44,575.1	1.22 × 10^9^	110,726.8	3.07 × 10^10^	48,568.52	1.53 × 10^10^	4.94 × 10^8^	208,724.2	5.69 × 10^8^	1.14 × 10^9^	4.14 × 10^9^	2.91 × 10^9^	12,373,368
	std	13,525.92	5.44 × 10^8^	18,472.61	6.5 × 10^9^	14,309.74	3.92 × 10^9^	2.01 × 10^8^	53,444.25	2.01 × 10^8^	2.9 × 10^8^	1.64 × 10^9^	1.44 × 10^9^	2,319,862
	median	35,717.23	2.16 × 10^8^	84,955.27	2.51 × 10^10^	36,251.46	9.75 × 10^9^	1.07 × 10^8^	145,667.7	1.98 × 10^8^	7.73 × 10^8^	1.11 × 10^9^	9.01 × 10^8^	9,755,304
	rank	1	8	3	13	2	12	6	4	7	9	11	10	5
C17-F16	mean	5289.667	8075.619	7346.94	23,585.12	5556.754	12,659.13	16,572.81	6401.656	6685.502	12,217.08	10,417.54	6132.963	11,108.18
	best	4897.6	7396.592	6037.364	18,423.11	4859.187	11,295.55	13,966.61	5961.599	6185.134	10,721.76	9217.053	5449.275	9424.787
	worst	5710.276	8970.729	8134.842	26,458.22	6867.361	14,021.14	18,721.12	6668.897	7453.341	13,860.22	11,540.53	6832.967	11,940.21
	std	332.3395	704.2064	923.0135	3635.585	892.9725	1121.277	1963.62	327.4517	604.6548	1289.506	962.7863	582.5351	1138.671
	median	5275.395	7967.578	7607.778	24,729.57	5250.235	12,659.92	16,801.76	6488.063	6551.766	12,143.17	10,456.29	6124.805	11,533.86
	rank	1	7	6	13	2	11	12	4	5	10	8	3	9
C17-F17	mean	4400.558	10,409.14	5990.338	8,696,852	4366.873	151,808.6	15,333.77	5168.306	5408.623	9341.009	228,490.8	7064.66	6756.812
	best	4055.441	5815.1	5796.742	2,357,254	4026.543	32,785.66	11,709.25	4917.866	4332.309	8097.868	42,794.52	6170.799	6191.594
	worst	4671.854	16,795.95	6476.642	20,011,852	4628.367	375,453	18,769.63	5547.375	6488.278	10,527.61	741,374.4	7828.836	7423.221
	std	260.7233	4852.693	326.2353	8,306,042	255.0694	158,483.7	3908.91	267.6024	896.4995	999.1039	342,134.4	692.6501	514.2877
	median	4437.468	9512.766	5843.985	6,209,152	4406.292	99,497.89	15,428.1	5103.992	5406.953	9369.278	64,897.14	7129.502	6706.217
	rank	2	9	5	13	1	11	10	3	4	8	12	7	6
C17-F18	mean	188,979.9	7,212,606	2,951,049	1.08 × 10^8^	235,943.2	18,747,096	20,370,306	3,891,643	9,340,657	20,568,868	8,970,756	2,463,637	7,316,713
	best	168,960.2	2,281,779	1,467,407	41,911,753	178,664.2	9,857,550	12,477,291	2,117,331	2,729,394	12,444,228	4,277,344	834,378.1	5,157,171
	worst	217,287.5	14,425,982	4,665,842	1.97 × 10^8^	379,813	28,848,858	31,924,135	7,854,026	15,928,695	38,379,799	11,074,720	3,768,395	10,061,294
	std	22,970	5,140,198	1,449,149	65,562,281	96,626.92	7,801,882	8256,895	2,663,425	5,635,043	12,131,498	3,152,116	1,271,998	2,343,646
	median	184,836	6071,331	2,835,474	96,283,852	192,647.90	18,140,988	18,539,900	2,797,608	9,352,270	15,725,723	10,265,479	2,625,888	7,024,194
	rank	1	6	4	13	2	10	11	5	9	12	8	3	7
C17-F19	mean	240,362.6	8.76 × 10^8^	3,017,804	2.34 × 10^10^	253,780.5	6.88 × 10^9^	1.99 × 10^8^	19,860,187	5.05 × 10^8^	9.51 × 10^8^	1.38 × 10^9^	5.71 × 10^8^	15,651,499
	best	106,471.1	33,323,526	1,155,276	1.71 × 10^10^	112,995.9	6.17 × 10^9^	73469347	6,784,792	13,099,458	4.24 × 10^8^	5.24 × 10^8^	1420743	5,304,591
	worst	317,561.9	2.12 × 10^9^	5,555,441	2.92 × 10^10^	335,639.9	8.01 × 10^9^	3.14 × 10^8^	31,723,596	1.91 × 10^9^	1.98 × 10^9^	2.57 × 10^9^	1.49 × 10^9^	35,863,459
	std	92,219.0	9.6 × 10^8^	1,859,799	4.98 × 10^9^	97,113.3	8.03 × 10^8^	1.01 × 10^8^	10,221,012	9.38 × 10^8^	6.96 × 10^8^	9.4 × 10^8^	6.8 × 10^8^	14,375,122
	median	268,708.6	6.73 × 10^8^	2,680,249	2.37 × 10^10^	283,243.1	6.67 × 10^9^	2.05 × 10^8^	20,466,180	48,052,465	7.03 × 10^8^	1.22 × 10^9^	3.96 × 10^8^	10,718,973
	rank	1	9	3	13	2	12	6	5	7	10	11	8	4
C17-F20	mean	4329.037	5209.792	6336.396	7771.453	4716.199	7113.265	6925.121	5395.872	4996.863	7387.463	5974.363	5170.578	6642.64
	best	3983.977	4821.265	5904.681	7612.301	4388.633	6767.715	6587.829	5094.733	4850.608	7234.322	5242.187	4710.582	6076.979
	worst	4539.248	5659.555	6676.751	7841.025	4978.188	7576.378	7332.808	5543.659	5163.653	7497.733	6314.946	5782.027	7037.92
	std	240.729	355.8995	343.9901	106.793	252.3931	386.9691	368.0945	203.5619	148.5221	125.1266	496.6382	474.2739	420.3355
	median	4396.461	5179.175	6382.075	7816.242	4748.988	7054.484	6889.924	5472.548	4986.596	7408.899	6170.159	5094.852	6727.831
	rank	1	5	8	13	2	11	10	6	3	12	7	4	9
C17-F21	mean	2736.167	3745.407	3729.792	4508.594	2838.871	4334.393	4315.533	3017.185	3055.55	3655.302	4547.086	3650.019	3629.856
	best	2679.86	3690.774	3506.549	4427.007	2768.495	4120.796	3872.558	2972.154	2900.463	3567.526	4387.706	3423.076	3557.698
	worst	2813.182	3796.665	3875.17	4568.519	2890.906	4510.86	4750.77	3054.39	3136.03	3719.88	4719.22	3851.77	3712.51
	std	58.0787	43.77476	159.0511	61.58535	58.50085	166.9908	385.4136	35.2477	105.7954	70.19626	135.5839	178.1641	71.99652
	median	2725.81	3747.09	3768.73	4519.43	2848.04	4352.96	4319.40	3021.10	3092.85	3666.90	4540.71	3662.61	3624.61
	rank	1	9	8	12	2	11	10	3	4	7	13	6	5
C17-F22	mean	16,754.43	22,635.78	20,677.34	34,479.41	19,077.25	32,106.98	30,302.25	19,243.96	18,528.36	34,487.37	20,703.73	20,184.32	29,011.38
	best	15,651.14	21,193.53	19,202.46	34,027.03	18,107.02	29,368.93	29,139.08	17,859.86	17,156.79	33,489	19,451.88	17,961.85	28,178.06
	worst	17,268.55	23,665.76	22,710.46	35,121.42	20,362.55	33,216.89	31,342.78	20,447.75	19,437	35,320.04	21,879.51	21,096.56	30,184.34
	std	754.6418	1037.438	1555.121	491.9479	953.8498	1830.945	1029.101	1331.092	1001.715	894.5323	1039.709	1497.787	894.7833
	median	17,049.01	22,841.92	20,398.22	34,384.6	18,919.72	32,921.05	30,363.58	19,334.13	18,759.82	34,570.22	20,741.76	20,839.44	28,841.56
	rank	1	8	6	12	3	11	10	4	2	13	7	5	9
C17-F23	mean	3330.207	5009.335	4180.505	5475.558	3302.335	5508.514	5381.020	3553.843	3630.727	4364.402	7806.769	5035.024	4491.184
	best	3220.897	4915.96	4094.759	5190.584	3192.09	5331.809	5167.214	3462.843	3529.972	4221.49	7299.394	4779.893	4285.936
	worst	3407.188	5150.114	4271.186	5700.211	3376.761	5801.627	5828.837	3670.808	3713.651	4491.014	8053.976	5209.153	4806.089
	std	84.12463	101.5099	83.55801	210.9503	83.8719	203.2764	302.6356	90.02939	75.82554	126.8273	353.6352	191.2664	233.4911
	median	3346.372	4985.632	4178.037	5505.718	3320.244	5450.31	5264.014	3540.861	3639.643	4372.552	7936.852	5075.525	4436.355
	rank	2	8	5	11	1	12	10	3	4	6	13	9	7
C17-F24	mean	3838.394	6619.833	5501.153	10,952.73	4019.698	7130.901	6669.652	4156.191	4463.04	4946.4	10,577.72	6083.567	5573.038
	best	3804.994	6393.899	5264.016	7257.544	3965.063	6181.58	5802.949	3992.141	4329.01	4779.776	10,344.84	6038.202	5498.956
	worst	3880.947	6956.558	5697.902	13,415.8	4076.189	7864.329	7222.435	4387.281	4672.438	5127.243	10,849.330	6123.814	5654.398
	std	33.5964	240.8529	193.1216	2980.522	55.04538	703.9283	626.4185	170.6274	146.9678	153.8419	268.4815	40.94055	69.16896
	median	3833.817	6564.438	5521.346	11,568.79	4018.771	7238.848	6826.611	4122.67	4425.356	4939.289	10,558.35	6086.125	5569.398
	rank	1	9	6	13	2	11	10	3	4	5	12	8	7
C17-F25	mean	3419.878	12,914.68	4200.03	22,463.79	3727.179	10,109.35	7602.511	3458.415	6612.48	9840.617	13,003.17	6315.64	8226.567
	best	3270.776	10,959.75	3793.055	20,802.69	3639.544	7389.433	7500.673	3391.467	5802.505	7855.257	11,232.45	5406.982	7683.57
	worst	3570.643	15,484.74	4584.833	26,151.87	3817.394	12,792.82	7828.874	3519.4	7077.109	13,190.3	14,733.21	7810.14	8659.75
	std	127.2128	2106.496	327.3314	2514.302	84.07639	2611.297	152.0693	67.05617	559.221	2384.22	1600.964	1069.279	439.551
	median	3419.046	12,607.12	4211.116	21,450.29	3725.889	10,127.58	7540.248	3461.397	6785.152	9158.458	13,023.5	6022.719	8281.474
	rank	1	11	4	13	3	10	7	2	6	9	12	5	8
C17-F26	mean	12,161.53	33,589.15	25,812.52	47,915.4	12,417.5	36,882.86	39,697.02	13,011.43	16,796.16	26,337.9	36,037.68	26,179.06	23,813.48
	best	11,570.58	32,040.89	22,771.24	45,204.13	12,010.75	34,702.39	36,284.73	12,173.32	16,104.17	23,268.42	34,617.49	23,436.07	18,794.69
	worst	13,140.16	35,062.47	28,918.45	49,592	13,166.37	39,236.38	46,080.27	14,038	18,257.86	31,568.31	38,296.26	29,012.3	30,130.64
	std	714.7895	1291.344	2627.91	2110.57	517.0592	2305.593	4571.018	774.4961	986.1479	3755.775	1584.211	2288.35	5129.716
	median	11,967.7	33,626.62	25,780.19	48,432.75	12,246.44	36,796.33	38,211.53	12,917.19	16,411.3	25,257.43	35,618.49	26,133.95	23,164.3
	rank	1	9	6	13	2	11	12	3	4	8	10	7	5
C17-F27	mean	3573.983	7203.719	4220.107	12,874.15	3543.702	6590.686	5549.819	3636.355	4351.534	4365.099	14,390.47	4068.458	5427.951
	best	3550.019	6374.999	4035.689	9574.128	3515.753	5450.785	5255.732	3538.111	4047.274	4231.086	12,460.66	3698.791	5163.581
	worst	3617.97	8029.224	4525.976	16,299.41	3586.981	7337.146	6286.109	3743.107	4728.147	4538.464	16,773.18	4534.344	5728.043
	std	30.36471	874.2169	212.0854	3625.498	30.41211	825.7145	492.4035	87.09758	298.1583	144.9164	1847.757	369.1118	238.5253
	median	3563.971	7205.326	4159.381	12,811.52	3536.036	6787.406	5328.717	3632.1	4315.358	4345.422	14,164.01	4020.349	5410.089
	rank	2	11	5	12	1	10	9	3	6	7	13	4	8
C17-F28	mean	3582.808	16,810.98	4799.097	29,879.23	3850.162	16,533.6	11,426.62	3532.959	10765.53	11,980.69	17,423.16	6348.414	13,091.11
	best	3420.284	15,333.26	4474.553	26,738.46	3777.292	14,884.29	9122.899	3454.509	8221.553	9544.848	15,852.57	4956.941	12,054.66
	worst	3659.831	18,085.58	5038.336	33,810.2	3972.019	18,597.99	13,140.11	3636.308	14,812.54	14,940.48	19,363.45	7865.142	14,211.12
	std	110.8076	1313.643	238.6427	2965.76	86.65758	1651.567	1959.457	75.76293	2827.535	2230.037	1666.674	1328.374	1159.838
	median	3625.557	16,912.53	4841.749	29,484.12	3825.668	16,326.07	11,721.74	3520.51	10,014.01	11,718.71	17,238.3	6285.787	13,049.32
	rank	2	11	4	13	3	10	7	1	6	8	12	5	9
C17-F29	mean	6785.358	11,200.52	9945.999	371,294.3	7011.439	29,447.41	16,930.69	8701.23	8650.05	14,358.73	21,205.48	8372.447	12,438.74
	best	6237.576	10,204.3	8591.844	199,089.7	6512.523	15,174.79	13,802.42	7941.767	7491.625	13,504.12	16,011.96	7665.661	12,035.03
	worst	7296.052	11,863.42	10,788.87	515,537.6	7399.818	66,248.24	21,278.25	9252.116	10,249.87	15,398.75	24,203.99	8683.496	13,143.18
	std	489.398	754.6761	947.033	134,866.2	422.6678	24,574.85	3132.304	552.0585	1345.612	871.218	3595.977	482.8703	485.136
	median	6803.901	11,367.19	10,201.64	385,275.00	7066.708	18,183.31	16,321.06	8805.519	8429.351	14,266.02	22,302.99	8570.316	12,288.36
	rank	1	7	6	13	2	12	10	5	4	9	11	3	8
C17-F30	mean	5,554,314	2.47 × 10^9^	29,428,850	4 × 10^10^	6,188,795	1.46 × 10^10^	1.17 × 10^9^	1.19 × 10^8^	2.32 × 10^9^	3.05 × 10^9^	1.18 × 10^10^	3.93 × 10^8^	6.24 × 10^8^
	best	2,780,134	1.38 × 10^8^	16,769,683	3.74 × 10^10^	2,886,968	1.32 × 10^10^	8.85 × 10^8^	85,223,927	5.3 × 10^8^	1.18 × 10^9^	8.16 × 10^9^	7358589	4.39 × 10^8^
	worst	7,910,687	6.19 × 10^9^	51,751,386	4.33 × 10^10^	8,330,799	1.64 × 10^10^	1.6 × 10^9^	1.65 × 10^8^	5.4 × 10^9^	4.23 × 10^9^	1.56 × 10^10^	1.52 × 10^9^	1.04 × 10^9^
	std	2,148,328	2.6 × 10^9^	15,661,651	2.53 × 10^9^	2,560,052	1.43 × 10^9^	3.08 × 10^8^	34,020,142	2.12 × 10^9^	1.31 × 10^9^	3.05 × 10^9^	7.51 × 10^8^	2.83 × 10^8^
	median	5,763,217	1.77 × 10^9^	24,597,166	3.97 × 10^10^	6,768,707	1.45 × 10^10^	1.1 × 10^9^	1.13 × 10^8^	1.68 × 10^9^	3.4 × 10^9^	1.17 × 10^10^	22,288,328	5.08 × 10^8^
	rank	1	9	3	13	2	12	7	4	8	10	11	5	6
Sum rank		35	244	144	359	61	318	275	116	159	255	281	172	220
Mean rank		1.206897	8.413793	4.965517	12.37931	2.103448	10.96552	9.482759	4	5.482759	8.793103	9.689655	5.931034	7.586207
Total rank		1	8	4	13	2	12	10	3	5	9	11	6	7

**Table 6:** Optimization results of the CEC 2019 test suite.

		SOA	WSO	AVOA	RSA	MPA	TSA	WOA	MVO	GWO	TLBO	GSA	PSO	GA
C19-F1	mean	1	71,754.43	1	1	1	20,197.74	11,700,900	1,132,771	27,189.42	68.36027	6.2 × 10^8^	149,550.8	7,917,670
	best	1	1160.891	1	1	1	2.7537	2,455,600	376,289.6	1.003811	1.000003	1.49 × 10^8^	14,365.34	2,787,375
	worst	1	205,465.8	1	1	1	80,619	16,679,000	2,285,093	94,712.58	270.415	1.07 × 10^9^	475,601.4	16,682,079
	std	0	92,622.55	0	0	0	40,280.89	6,367,086	841,143.6	45,495.01	134.7032	4.66 × 10^8^	218,241.3	6,534,856
	median	1	40,195.5	1	1	1	84.6	13,834,500	934,851.2	7022.04	1.013028	6.33 × 10^8^	54,118.3	6,100,613
	rank	1	5	1	1	1	3	9	7	4	2	10	6	8
C19-F2	mean	3.1203	172.9367	4.694109	5	3.569175	800.59	6139.5	563.8434	454.4571	667.1054	27,745.11	363.9978	857.5801
	best	2.607813	98.73351	4.303868	5	2.798006	629.09	2908	286.6219	210.5741	5.564019	9851.265	226.8182	577.9538
	worst	3.596576	266.8291	5	5	4.344219	937.15	9275.1	838.1335	658.0928	1190.362	42,469.39	544.8626	1135.415
	std	0.409111	70.34908	0.359864	5.13 × 10^−16^	0.631625	136.5541	2632.46	227.506	184.6327	537.804	14,988.08	133.4324	290.0151
	median	3.138405	163.0922	4.736285	5	3.567238	818.06	6187.45	565.309	474.5807	736.2478	29,329.9	342.1552	858.4759
	rank	1	5	3	4	2	10	12	8	7	9	13	6	11
C19-F3	mean	1.250139	1.892155	2.126998	8.138315	1.423922	6.45255	6.1394	8.960775	1.889948	4.716604	4.003168	3.777545	5.779726
	best	1.045965	1.409789	1.409196	6.294517	1.196344	1.7408	3.0642	7.711774	1.141124	4.149536	2.763422	1.409135	3.599784
	worst	1.468114	2.377011	3.342791	9.21189	1.563064	9.7143	9.6473	10.70855	3.407555	5.801678	5.911327	7.687041	7.700128
	std	0.23558	0.555352	0.920308	1.299598	0.165362	3.397994	2.740517	1.498499	1.026228	0.751039	1.382805	3.010391	1.849209
	median	1.243239	1.890909	1.878002	8.523426	1.46814	7.17755	5.92305	8.71139	1.505556	4.457601	3.668962	3.007002	5.909496
	rank	1	4	5	12	2	11	10	13	3	8	7	6	9
C19-F4	mean	8.992048	17.42558	35.32589	73.96348	11.44685	53.1035	59.77375	25.557	23.2038	35.43137	51.99142	18.90922	22.35515
	best	3.019184	13.93534	8.959667	55.8996	7.9647	41.459	32.984	22.89004	10.10581	32.68921	39.80328	10.94959	19.10672
	worst	12.96868	21.89463	57.7123	94.37194	14.929	65.757	92.985	29.57352	38.80844	41.06531	60.69719	36.81838	27.48026
	std	4.53174	4.092737	20.23611	16.55228	3.493927	11.06971	26.37413	2.951315	13.70501	3.931349	9.096213	12.04949	3.586093
	median	9.990163	16.93617	37.31579	72.79118	11.44685	52.599	56.563	24.88222	21.95048	33.98547	53.73261	13.93446	21.41682
	rank	1	3	8	13	2	11	12	7	6	9	10	4	5
C19-F5	mean	1.050836	1.688348	1.201342	88.27602	1.034475	29.61225	2.0399	1.392442	1.595406	2.934297	1.136918	1.213511	1.725773
	best	1.047471	1.210439	1.10852	66.19828	1.0074	13.555	1.6941	1.239289	1.347328	2.63866	1.066225	1.179585	1.550766
	worst	1.057175	2.265097	1.285169	108.9916	1.0616	62.067	2.3591	1.756175	1.893882	3.143506	1.205143	1.253634	1.841835
	std	0.004577	0.456641	0.07586	17.49218	0.028578	22.85461	0.279808	0.246618	0.258445	0.225516	0.070591	0.038228	0.125116
	median	1.049349	1.638929	1.205841	88.95708	1.03445	21.4135	2.0532	1.287153	1.570207	2.977511	1.138152	1.210412	1.755246
	rank	2	8	4	13	1	12	10	6	7	11	3	5	9
C19-F6	mean	1.074498	2.901374	7.091154	9.942598	1.192698	7.857175	10.6821	3.023598	3.494285	4.576521	4.111191	3.643596	3.227866
	best	1.036486	1.801215	6.214133	9.456187	1.074	4.3964	9.3754	1.226604	1.335962	3.587161	1.139357	1.531474	2.490765
	worst	1.179165	3.603571	9.322565	10.7512	1.321624	10.543	11.969	4.363759	4.995392	5.230096	5.652704	6.240727	4.391409
	std	0.06988	0.794252	1.496419	0.606401	0.104412	2.598436	1.062426	1.370934	1.548655	0.722828	2.109974	1.980984	0.835364
	median	1.04117	3.100355	6.41396	9.781504	1.187584	8.24465	10.692	3.252014	3.822894	4.744412	4.826352	3.401092	3.014646
	rank	1	3	10	12	2	11	13	4	6	9	8	7	5
C19-F7	mean	246.8228	487.5351	1082.794	1730.98	302.0954	1406.9	1141.905	1056.615	1177.53	1175.092	1660.543	1151.383	707.8292
	best	150.4273	282.8179	803.7554	1577.123	165.3973	1022	702.62	723.0297	1021.721	708.6485	1502.922	658.1489	490.5917
	worst	328.0506	758.795	1546.282	1815.608	451.11	1685.7	1794.3	1850.827	1376.227	1521.911	1797.54	1606.777	1093.745
	std	73.04863	211.9359	352.957	108.8781	117.496	279.3649	463.0885	533.2837	167.1757	370.338	133.3842	444.184	282.0167
	median	254.4067	454.2637	990.5686	1765.594	295.9372	1459.95	1035.35	826.3016	1156.086	1234.905	1670.856	1170.303	623.4902
	rank	1	3	6	13	2	11	7	5	10	9	12	8	4
C19-F8	mean	2.981774	3.730856	4.468653	4.818338	3.3539	4.1504	4.84945	4.059096	3.609261	4.301578	5.216925	4.608137	4.529276
	best	2.825294	3.676426	3.927288	4.538189	3.010552	3.5611	4.607	3.561631	3.366284	4.055844	5.119708	4.369611	4.30402
	worst	3.104412	3.84246	4.89483	4.985393	3.6811	4.9029	5.082	4.484265	4.036074	4.870075	5.373209	4.978318	4.669163
	std	0.131505	0.07541	0.456051	0.200563	0.338951	0.562938	0.210516	0.436397	0.302651	0.381754	0.12212	0.264886	0.164295
	median	2.998694	3.702269	4.526247	4.874885	3.361974	4.0688	4.8544	4.095245	3.517342	4.140198	5.187391	4.542309	4.571962
	rank	1	4	8	11	2	6	12	5	3	7	13	10	9
C19-F9	mean	1.081897	1.175838	1.393224	3.105656	1.092347	1.350775	1.3803	1.176442	1.220459	1.324073	1.196267	1.193862	1.138063
	best	1.063102	1.122186	1.078336	2.401267	1.0512	1.1981	1.1917	1.150186	1.1062	1.253609	1.078801	1.089627	1.11366
	worst	1.115502	1.242474	1.604168	3.684131	1.123	1.5607	1.6508	1.203633	1.355802	1.384995	1.315911	1.263166	1.184152
	std	0.024024	0.054244	0.22406	0.546042	0.033978	0.161533	0.21688	0.024786	0.113289	0.06944	0.11506	0.075043	0.031644
	median	1.074491	1.169346	1.445197	3.168613	1.097595	1.32215	1.33935	1.175974	1.209917	1.328844	1.195178	1.211327	1.12722
	rank	1	4	12	13	2	10	11	5	8	9	7	6	3
C19-F10	mean	18.03383	17.89517	21.03628	21.46784	21.00075	21.41375	21.212	21.02743	21.47564	21.43069	23.24989	21.03227	21.26732
	best	6.811592	7.37422	20.97165	21.37514	21	21.355	21.047	21.00739	21.46041	21.39861	20.99799	20.99969	21.10764
	worst	22.01547	21.54158	21.17207	21.54733	21.003	21.538	21.594	21.04061	21.49669	21.50204	24.54712	21.12946	21.39193
	std	7.483952	7.014666	0.091558	0.070798	0.0015	0.084215	0.256657	0.014164	0.017614	0.048814	1.666164	0.064791	0.126423
	median	21.65414	21.33245	21.0007	21.47445	21	21.381	21.1035	21.03087	21.47273	21.41107	23.72722	20.99997	21.28486
	rank	2	1	6	11	3	9	7	4	12	10	13	5	8
Sum rank		12	40	63	103	19	94	103	64	66	83	96	63	71
Mean rank		1.2	4	6.3	10.3	1.9	9.4	10.3	6.4	6.6	8.3	9.6	6.3	7.1
Total rank		1	3	4	11	2	9	11	5	6	8	10	4	7

**Table 7:** Wilcoxon rank sum test results.

Compared Algorithm	Types of Objective Functions				
	CEC 2017				CEC 2019
	d=10	d=30	d=50	d=100	
SOA vs. WSO	1.93 × 10^−15^	2.02 × 10^−21^	2.02 × 10^−21^	1.97 × 10^−21^	2.63 × 10^−7^
SOA vs. AVOA	6.1 × 10^−20^	1.16 × 10^−20^	1.97 × 10^−21^	1.97 × 10^−21^	2.07 × 10^−5^
SOA vs. RSA	2.02 × 10^−21^	1.97 × 10^−21^	1.97 × 10^−21^	1.97 × 10^−21^	2.79 × 10^−7^
SOA vs. MPA	0.025318	1.36 × 10^−5^	1.86 × 10^−12^	1.74 × 10^−15^	0.000237
SOA vs. TSA	2.24 × 10^−21^	1.97 × 10^−21^	1.97 × 10^−21^	1.97 × 10^−21^	1.18 × 10^−7^
SOA vs. WOA	1.97 × 10^−21^	1.97 × 10^−21^	1.97 × 10^−21^	1.97 × 10^−21^	1.48 × 10^−7^
SOA vs. MVO	2.77 × 10^−19^	5.13 × 10^−19^	1.56 × 10^−20^	2.8 × 10^−20^	4.98 × 10^−7^
SOA vs. GWO	7.23 × 10^−20^	4.62 × 10^−21^	3.69 × 10^−21^	4.74 × 10^−21^	1.71 × 10^−6^
SOA vs. TLBO	3.78 × 10^−21^	1.97 × 10^−21^	1.97 × 10^−21^	1.97 × 10^−21^	1.97 × 10^−7^
SOA vs. GSA	7.23 × 10^−20^	1.97 × 10^−21^	2.73 × 10^−21^	1.97 × 10^−21^	3.57 × 10^−8^
SOA vs. PSO	2.42 × 10^−20^	7.79 × 10^−21^	2.07 × 10^−21^	1.97 × 10^−21^	7.06 × 10^−7^
SOA vs. GA	4.18 × 10^−21^	2.02 × 10^−21^	1.97 × 10^−21^	1.97 × 10^−21^	1.71 × 10^−7^

**Table 8:** Optimization results of the CEC 2011 test suite.

		SOA	WSO	AVOA	RSA	MPA	TSA	WOA	MVO	GWO	TLBO	GSA	PSO	GA
C11-F1	mean	3.703267	15.02557	25.74562	26.00412	4.069952	17.52275	21.89831	13.4942	15.568	20.13739	25.86975	21.49811	25.49193
	best	2.68 × 10^−10^	11.75673	24.83901	25.15683	2.85 × 10^−10^	8.510385	17.24132	6.548961	10.72236	17.39148	24.1914	17.49104	24.75272
	worst	14.81307	19.29177	26.94225	27.78769	16.27981	21.63138	24.82611	16.5131	22.07106	22.56158	27.76547	25.58579	26.07053
	std	7.406534	3.129599	1.051112	1.209479	8.139905	6.077377	3.257521	4.67819	5.389997	2.179018	1.524199	3.753819	0.558841
	median	1.37 × 10^−9^	14.52688	25.60061	25.53597	1.48 × 10^−9^	19.97462	22.7629	15.45736	14.7393	20.29825	25.76107	21.45781	25.57223
	rank	1	4	11	13	2	6	9	3	5	7	12	8	10
C11-F2	mean	−25.5074	−22.3944	−11.6301	−8.06028	−24.8695	−6.49474	−15.4353	−9.34108	−21.6587	−8.61104	−9.40185	−22.0875	−13.1955
	best	−26.343	−22.8813	−14.0599	−8.64276	−25.7669	−9.42193	−20.4428	−12.3034	−24.5033	−9.39253	−13.4981	−24.4758	−14.0636
	worst	−24.2869	−21.4054	−9.06418	−7.42364	−23.6464	−4.02889	−11.7159	−7.01703	−15.7941	−7.55467	−6.03461	−19.675	−12.6002
	std	1.004079	0.674967	2.043459	0.550565	0.996461	2.542635	4.065062	2.333323	3.960315	0.858825	3.099445	2.334602	0.625598
	median	−25.6998	−22.6454	−11.6981	−8.08736	−25.0324	−6.26406	−14.7912	−9.02192	−23.1687	−8.74848	−9.03735	−22.0996	−13.0591
	rank	1	3	8	12	2	13	6	10	5	11	9	4	7
C11-F4	mean	−34.1366	−29.4024	−17.5227	−16.7272	−33.0629	−27.8822	−23.8592	−27.1652	−31.4271	−14.1604	−26.2837	−7.58	−8.27727
	best	−34.4821	−33.8574	−19.4025	−19.341	−34.2193	−31.2664	−27.0822	−31.5712	−34.2156	−20.773	−34.1647	−11.3148	−12.0614
	worst	−33.4793	−27.2841	−16.1919	−13.8236	−31.812	−25.793	−21.0792	−24.2788	−26.2364	−10.1154	−21.2736	−3.0134	−4.22734
	std	0.452345	3.068482	1.37731	2.848936	0.998888	2.51344	2.466816	3.106749	3.57637	4.888331	5.749063	3.428548	3.376156
	median	−34.2925	−28.234	−17.2482	−16.8722	−33.1102	−27.2347	−23.6377	−26.4055	−32.6282	−12.8767	−24.8482	−7.99588	−8.41014
	rank	1	4	9	10	2	5	8	6	3	11	7	13	12
C11-F5	mean	−27.1938	−19.3848	−10.0921	−10.5136	−25.6126	−12.3726	−14.1942	0	−22.2141	−5.21334	−14.2828	0	0
	best	−27.4298	−23.0059	−14.7254	−11.5854	−26.5001	−19.429	−15.0878	0	−26.8272	−12.1923	−16.9124	0	0
	worst	−26.4862	−16.8457	−7.09818	−9.51564	−23.0059	0	−12.4219	0	−19.5116	0	−10.6775	0	0
	std	0.471752	2.647173	3.382754	1.153367	1.73799	8.505039	1.25671	0	3.488744	6.19006	3.109174	0	0
	median	−27.4297	−18.8438	−9.27249	−10.4768	−26.4723	−15.0308	−14.6336	0	−21.2588	−4.33054	−14.7707	0	0
	rank	1	4	9	8	2	7	6	11	3	10	5	11	11
C11-F6	mean	0.89073	1.14754	2.024755	2.155071	0.90234	1.643725	2.052031	0.965717	1.048196	1.874535	1.082047	1.180848	2.052041
	best	0.866406	0.998087	1.6614	1.83069	0.841922	1.560405	1.793715	0.833545	0.938764	1.614306	0.905594	1.008846	1.855107
	worst	0.934629	1.258533	2.187947	2.345592	0.966592	1.831218	2.309743	1.247127	1.146828	2.007636	1.387668	1.423934	2.215405
	std	0.030907	0.129835	0.247021	0.227085	0.053749	0.125878	0.234016	0.190355	0.085177	0.17657	0.224094	0.176804	0.15728
	median	0.880943	1.16677	2.124836	2.222001	0.900423	1.591638	2.052333	0.891097	1.053595	1.938099	1.017464	1.145306	2.068825
	rank	1	6	10	13	2	8	11	3	4	9	5	7	12
C11-F7	mean	231.7975	223.6592	276.5	349.25	220	234	291.25	220	224.5	227	231.5	329.9552	229.8177
	best	220	220	230	299	220	220	238	220	220	220	220	220	220
	worst	239.4414	234.6368	323	404	220	258	333	220	238	238	248	535.8208	259.2707
	std	8.387818	7.318376	40.02083	43.06874	0	18.11077	39.55903	0	9	8.717798	13.89244	141.2683	19.63537
	median	233.8743	220	276.5	347	220	229	297	220	220	225	229	282	220
	rank	7	2	9	12	1	8	10	1	3	4	6	11	5
C11-F8	mean	7306.049	735,450.4	1,029,600	1301,689	7878.018	96,263.48	401,545.2	127,251.3	29,143.13	549,851	998,962.5	1,637,763	2,186,754
	best	4355.355	547,467.2	914,559	848,186.4	4665.333	69,896.59	323,827.5	88,847.38	17,099.15	377,823.8	955,606.7	616,975.9	2,097,586
	worst	11,321.75	873,535.9	1,112,084	1,528,229	12,237.1	134,708.9	547,008.1	164,393.9	42,638.2	747,382	1,052,476	3,466,500	2,273,623
	std	3118.179	147,088.4	86,808.81	308,299.6	3430.68	27,889.49	103,098.1	30,939.54	12,355.31	152,022.1	42,600.87	1256,071	72,053.94
	median	6773.544	760,399.2	1,045,878	1,415,170	7304.821	90,224.2	367,672.5	127,881.9	28,417.59	537,099	993,883.9	1,233,788	2,187,904
	rank	1	8	10	11	2	4	6	5	3	7	9	12	13
C11-F9	mean	−21.1519	−16.4886	−8.89083	−10.1972	−19.7224	−12.2849	−9.69672	−17.4831	−11.0572	−10.2549	−11.3843	−10.2803	−10.0705
	best	−21.5356	−18.7915	−9.07622	−10.5958	−20.7818	−15.2414	−10.2871	−21.2049	−12.9979	−10.2865	−11.8241	−10.364	−10.1474
	worst	−20.6824	−14.5174	−8.70287	−9.81793	−16.9694	−10.5739	−8.59883	−12.3942	−10.3885	−10.2262	−10.935	−10.2224	−9.99439
	std	0.418453	2.099664	0.19497	0.317858	1.843503	2.110651	0.788387	4.054017	1.294026	0.027217	0.446827	0.06044	0.066287
	median	−21.1947	−16.3228	−8.89211	−10.1875	−20.5693	−11.6621	−9.95046	−18.1667	−10.4213	−10.2535	−11.389	−10.2674	−10.0701
	rank	1	4	13	10	2	5	12	3	7	9	6	8	11
C11-F10	mean	275,953.6	293,843.2	1,728,003	10,733,179	507,841.1	6814,247	1668,923	1969,755	4265,332	5,689,705	1,570,136	5710,499	6,730,921
	best	111,906.1	119,680.6	1,671,593	10,440,242	443,295.4	5637,001	1596,987	1584,500	3731,297	5,689,705	1,439,379	5689,705	6,711,548
	worst	435,202.3	457,974.2	1,774,439	10,907,934	577,384.2	8,101,877	1718,805	2559,982	4585,233	5,689,705	1,703,594	5748,435	6,752,137
	std	133,300.2	140,150.7	43,954.16	203,662.6	57,922.72	1,169,664	51,360.73	465,329.2	378,888.6	0	128,768.8	27,792.26	17,639.59
	median	278,353	298,858.9	1,732,991	10,792,271	505,342.4	6,759,054	1,679,950	1,867,268	4,372,400	5,689,705	1,568,786	5,701,929	6,729,999
	rank	1	2	6	13	3	12	5	7	8	9	4	10	11
C11-F11	mean	1244,166	3917,084	7200,862	15,954,845	1,205,197	6,003,707	6,372,956	1,356,302	1,588,951	15,505,519	6777,124	2,450,810	15,381,122
	best	1115,988	3701,366	6,994,873	14,807,307	1,087,931	4,992,934	5,869,317	1,237,960	1,378,205	14,999,220	6594,614	2,214,801	14,242,937
	worst	1331,317	4098,419	7,301,082	16,959,124	1,287,550	7,191,303	6,835,128	1,627,660	2,025,660	15,937,381	7026,405	2,558,421	16,446,911
	std	91,442.38	176,574.8	139,296.1	882,680.7	84,212.59	903,553.7	417,842.9	184,571.9	300,145.8	397,440.9	207,663.9	158,822.7	935,503.1
	median	1,264,680	3,934,275	7,253,747	16,026,475	1,222,653	5,915,295	6,393,689	1,279,795	1,475,969	15,542,737	6,743,739	2,515,010	15,417,320
	rank	2	6	10	13	1	7	8	3	4	12	9	5	11
C11-F12	mean	15,444.2	15,775.42	15,454.67	16,506.66	16,390.34	15,566.71	15,506.98	15,488.7	15,519.23	15,935.78	129,995.2	15,515.88	16,052.38
	best	15,444.19	15,444.23	15,451.9	15,996.18	15,444.21	15,529.77	15,484.73	15,469.18	15,500.74	15,514.42	113,048.8	15,477.7	15,489.97
	worst	15,444.21	16,732.14	15,457.72	17,774.94	16,966.72	15,613.13	15,533.89	15,505.51	15,547.46	16,670.71	151,977.1	15,555.92	17,025.63
	std	0.010596	637.9726	2.451103	852.4752	667.7195	35.95627	24.013	14.9133	20.19086	541.7847	16,644.88	33.017	694.0509
	median	15,444.19	15,462.66	15,454.53	16,127.77	16,575.22	15,561.97	15,504.65	15,490.05	15,514.36	15,778.99	127477.5	15,514.94	15,846.96
	rank	1	8	2	12	11	7	4	3	6	9	13	5	10
C11-F13	mean	18,808.95	18,401.87	18,913.78	276,941.8	18,308.93	19,373.02	19,262.54	19,316.49	19,545.76	108,292.5	19,295.41	19,116.86	19,139.07
	best	18,583.4	18,212.02	18,679.04	202,857.5	18,281.42	19,118.13	19,044.16	19,136.48	19,340.25	42,350.89	19,218.27	18,963.55	18,965.28
	worst	18,941.42	18,524.11	19,086.98	400,961	18,342.71	19,749.48	19,529.18	19,493.59	19,714.25	254,303.9	19,414.24	19,255.93	19,268.32
	std	156.5688	134.1286	181.804	88,734.65	31.09058	267.474	210.8087	146.6933	196.8206	98,161.29	84.17887	157.4519	144.5224
	median	18,855.48	18,435.69	18,944.54	251,974.4	18,305.8	19,312.23	19,238.41	19,317.94	19,564.27	68,257.65	19,274.56	19,123.97	19,161.34
	rank	3	2	4	13	1	10	7	9	11	12	8	5	6
C11-F14	mean	33,436.9	33,006.71	239,954.4	2,343,498	32,840.39	58,551.43	55,559.51	32,996.16	33,227.12	9,328,370	406,885.7	33,333.04	6,772,673
	best	33,126.18	32,844.61	61,174.85	975,713.9	32,786.83	33,205.38	33,070.23	32,880.13	33,076.3	5,245,604	353,167.1	33,320.61	4,619,131
	worst	33,831.7	33,126.57	439,827.3	6,129,448	32,874.34	133,374.7	122,653.1	33,127.33	33,382.38	13,754,921	429,323.5	33,357.17	10,429,108
	std	309.7042	134.8848	203,421.2	2,527,893	37.99649	49,883.49	44,729.12	107.7502	132.081	3,483,336	36,212.51	17.05479	2,694,081
	median	33,394.87	33,027.82	229,407.7	1,134,415	32,850.19	33,812.82	33,257.37	32,988.58	33,224.9	9,156,476	422,526.2	33,327.18	6,021,226
	rank	6	3	9	11	1	8	7	2	4	13	10	5	12
C11-F15	mean	131,896.2	140,692.8	138,044.1	2362,861	141,030	139,185.7	149,714.5	142,078	137,989.1	98,216,083	16,661,057	82,739,645	90,489,805
	best	129,599.2	134,258.9	134,145.7	551,007.8	136,822.8	134,983.1	143,326.3	137,708.7	134,926	81,712,714	145,480.7	67,023,611	77,222,102
	worst	133,406.6	151,586.1	142,366.4	5916,825	143,594.4	144,166.6	156,177.1	149,494.5	143,091.5	1.11 × 10^8^	42,521,588	1 × 10^8^	1.04 × 10^8^
	std	1692.118	7711.282	3367.715	2,413,071	3052.919	3843.03	5629.417	5157.713	3897.759	12,641,427	18,783,856	16,142,420	11,527,058
	median	132,289.6	138,463.1	137,832.2	1491,805	141,851.4	138,796.5	149,677.3	140,554.5	136,969.5	99,861,336	11,988,581	81,736,076	90,566,521
	rank	1	5	3	9	6	4	8	7	2	13	10	11	12
C11-F16	mean	1,992,168	4,080,675	6.39 × 10^9^	1.91 × 10^10^	1,931,384	1.7 × 10^9^	1.12 × 10^10^	3,335,018	2,977,526	2.45 × 10^10^	1.37 × 10^10^	2.05 × 10^10^	2.24 × 10^10^
	best	1,966,581	1,935,090	5.81 × 10^9^	1.37 × 10^10^	1,899,652	9.95 × 10^8^	8.91 × 10^9^	2,994,846	2,450,995	1.85 × 10^10^	1.1 × 10^10^	1.67 × 10^10^	2.1 × 10^10^
	worst	2,004,837	9,745,854	7 × 10^9^	2.33 × 10^10^	1,956,433	2.5 × 10^9^	1.29 × 10^10^	3,937,955	3,345,029	2.98 × 10^10^	1.55 × 10^10^	2.44 × 10^10^	2.39 × 10^10^
	std	17,340.9	3,793,542	5.24 × 10^8^	4.12 × 10^9^	24,490.05	6.31 × 10^8^	1.81 × 10^9^	435,811.6	379,800.1	5.09 × 10^9^	1.91 × 10^9^	3.47 × 10^9^	1.26 × 10^9^
	median	1,998,628	2,320,879	6.38 × 10^9^	1.96 × 10^10^	1,934,726	1.66 × 10^9^	1.15 × 10^10^	3,203,636	3,057,041	2.48 × 10^10^	1.41 × 10^10^	2.04 × 10^10^	2.24 × 10^10^
	rank	2	5	7	10	1	6	8	4	3	13	9	11	12
C11-F17	mean	966,310.6	1,616,414	16,449,493	1.45 × 10^8^	944,296.7	1909,746	12,427,380	986,840.3	1100,256	29,913,771	14,001,521	1.87 × 10^8^	1.28 × 10^8^
	best	952,773.8	1,094,971	9,228,474	1 × 10^8^	941,062.2	1271,774	4919,875	958,253.1	1018,907	26,861,276	11,017,330	1.75 × 10^8^	1.26 × 10^8^
	worst	979,957.6	2,285,712	29,396,167	1.66 × 10^8^	947,649.9	2280,117	25,716,414	1,030,102	1,340,216	37,140,318	17,250,112	2.03 × 10^8^	1.32 × 10^8^
	std	11,741.73	494,288.8	9,390,234	30,701,419	2718.591	440,403.5	9,750,591	30,602.88	159,976.7	4,871,672	2,620,744	14,016,796	3,160,298
	median	966,255.5	1,542,486	13,586,665	1.57 × 10^8^	944,237.3	2,043,546	9,536,616	979,503	1,020,951	27,826,745	13,869,321	1.85 × 10^8^	1.27 × 10^8^
	rank	2	5	9	12	1	6	7	3	4	10	8	13	11
C11-F18	mean	1,000,924	2,590,116	16,582,334	1.42 × 10^8^	1,069,181	2,937,110	14,575,361	1,710,525	1,589,011	35,041,852	15,310,972	1.81 × 10^8^	1.27 × 10^8^
	best	943,538.7	1,954,981	14,730,898	1.23 × 10^8^	997,145.1	2,414,231	5831,462	1,393,338	1,167,771	26,831,644	9,362,833	1.66 × 10^8^	1.26 × 10^8^
	worst	1,061,517	3,891,235	20,548,870	1.79 × 10^8^	1130,546	3,393,560	22,239,505	2,349,957	1,889,412	48,044,844	18,324,799	1.98 × 10^8^	1.28 × 10^8^
	std	52,438.1	879,404	2678,190	26,103,571	55,567.61	467,088.6	7,003,567	433,187.8	318,048.5	9,787,189	4,155,256	13,395,124	674,274.6
	median	999,320.4	2,257,125	15,524,785	1.34 × 10^8^	1,074,517	2,970,325	15,115,238	1,549,403	1,649,430	32,645,460	16,778,129	1.81 × 10^8^	1.26 × 10^8^
	rank	1	5	9	12	2	6	7	4	3	10	8	13	11
C11-F19	mean	955,907	3,058,450	14,619,890	1.54 × 10^8^	943,673	2,198,561	9,996,685	1,012,821	1,152,320	32,955,731	15,021,597	1.71 × 10^8^	1.27 × 10^8^
	best	946,161.6	1,019,342	12,702,812	1.34 × 10^8^	939,631	1,982,053	7,065,299	967,576.5	1,006,006	28,464,019	8,991,833	1.42 × 10^8^	1.25 × 10^8^
	worst	965,274.1	8,406,301	16,683,672	1.83 × 10^8^	945,155.6	2,462,708	16,377,179	1,042,643	1,410,250	38,222,795	20,803,805	1.98 × 10^8^	1.29 × 10^8^
	std	7877.253	3,580,771	1,652,290	20,568,980	2698.357	216,671.7	4,296,432	32,544.64	184,022.2	4361,221	5,235,721	26,365,907	21,75,679
	median	956,096.1	1,404,078	14,546,539	1.49 × 10^8^	944,952.7	2,174,742	8,272,131	1,020,533	1,096,512	32,568,056	15,145,374	1.73 × 10^8^	1.27 × 10^8^
	rank	2	6	8	12	1	5	7	3	4	10	9	13	11
C11-F20	mean	9.891135	18.43484	37.38995	91.01355	10.6478	33.26948	50.31143	30.14097	31.4193	106.5034	42.42906	99.89802	120.8118
	best	8.48708	17.10456	35.08192	66.45898	9.075328	27.26106	37.94496	24.44492	20.005	96.68012	36.61611	94.66104	117.9591
	worst	12.17501	20.77307	38.49468	115.651	12.96167	40.06704	54.83165	34.77377	39.31062	119.9895	48.12816	104.208	122.6439
	std	1.597904	1.636928	1.602459	21.7381	1.659265	5.379866	8.261372	4.569823	8.429287	10.42733	5.629644	4.140173	2.131154
	median	9.451225	17.93087	37.9916	90.97211	10.27711	32.87492	54.23456	30.67259	33.18078	104.672	42.48599	100.3615	121.3222
	rank	1	3	7	10	2	6	9	4	5	12	8	11	13
C11-F21	mean	15.56392	27.48089	47.41507	74.30445	16.73979	34.48503	50.05503	27.46992	27.25074	97.52848	77.12081	96.64377	90.22154
	best	11.96234	25.26953	41.00099	52.03457	12.7765	27.49964	44.78979	20.42011	24.60761	88.33535	63.26603	83.14479	55.52377
	worst	19.37189	32.64845	56.32061	85.38791	20.57706	46.01433	59.96269	34.30754	30.17099	110.6057	103.444	115.6542	116.5376
	std	3.098876	3.475444	7.085416	15.10545	3.240004	8.054631	7.08343	6.257589	2.843483	10.47165	17.91987	13.69907	26.29488
	median	15.46073	26.00279	46.16933	79.89766	16.8028	32.21307	47.73381	27.57601	27.11219	95.58645	70.8866	93.88804	94.41237
	rank	1	5	7	9	2	6	8	4	3	13	10	12	11
C11-F22	mean	3.703267	15.02557	25.74562	26.00412	4.069952	17.52275	21.89831	13.4942	15.568	20.13739	25.86975	21.49811	25.49193
	best	2.68 × 10^−10^	11.75673	24.83901	25.15683	2.85 × 10^−10^	8.510385	17.24132	6.548961	10.72236	17.39148	24.1914	17.49104	24.75272
	worst	14.81307	19.29177	26.94225	27.78769	16.27981	21.63138	24.82611	16.5131	22.07106	22.56158	27.76547	25.58579	26.07053
	std	7.406534	3.129599	1.051112	1.209479	8.139905	6.077377	3.257521	4.67819	5.389997	2.179018	1.524199	3.753819	0.558841
	median	1.37 × 10^−9^	14.52688	25.60061	25.53597	1.48 × 10^−9^	19.97462	22.7629	15.45736	14.7393	20.29825	25.76107	21.45781	25.57223
	rank	1	4	11	13	2	6	9	3	5	7	12	8	10
Sum rank		38	91	161	226	48	140	154	96	91	205	166	189	213
Mean rank		1.809524	4.333333	7.666667	10.7619	2.285714	6.666667	7.333333	4.571429	4.333333	9.761905	7.904762	9	10.14286
Total rank		1	3	7	12	2	5	6	4	3	10	8	9	11
Wilcoxon: *p*-value			1.81 × 10^−7^	1.09 × 10^−14^	1.71 × 10^−15^	0.522069	8.49 × 10^−15^	2.42 × 10^−14^	6.09 × 10^−10^	2.71 × 10^−12^	5.56 × 10^−15^	7.92 × 10^−15^	8.57 × 10^−14^	7.92 × 10^−15^

**Table 9:** Performance of optimization algorithms on the pressure vessel design problem.

Algorithm	Optimum Variables				Optimum Cost
	*T_s_*	*T_h_*	*R*	*L*	
SOA	0.778027	0.384579	40.31228	200	5882.901
WSO	0.778027	0.384579	40.31228	200	5882.901
AVOA	0.778027	0.384579	40.31228	200	5882.901
RSA	0.873804	0.743654	41.80167	200	7974.077
MPA	0.778027	0.384579	40.31228	200	5882.901
TSA	0.780589	0.390787	40.34795	200	5925.31
WOA	0.956571	0.48327	49.13929	105.2553	6351.3
MVO	0.860496	0.426361	44.58435	148.0464	6044.069
GWO	0.779811	0.38726	40.39606	198.8451	5892.513
TLBO	1.997346	0.976593	75.72573	77.86602	24,264.99
GSA	1.129784	0.558452	58.53792	108.2202	9777.026
PSO	1.381856	0.696892	60.63946	169.9479	16,744.76
GA	0.962225	2.050866	47.54579	183.9335	14,893.63

**Table 10:** Statistical results of optimization algorithms on pressure vessel design problem.

Algorithm	Mean	Best	Worst	Std	Median	Rank
SOA	5882.901	5882.901	5882.901	1.87 × 10^−12^	5882.901	1
WSO	5906.961	5882.901	6118.936	58.51093	5882.901	3
AVOA	6318.02	5883.453	7029.777	356.8045	6324.189	6
RSA	12,261.31	7974.077	20,310.05	3514.19	11,063.85	9
MPA	5889.237	5883.856	5893.836	2.708182	5888.898	2
TSA	6257.625	5925.31	7013.933	368.492	6102.727	5
WOA	7683.151	6351.3	11,250.02	1293.839	7295.016	8
MVO	6668.44	6044.069	7322.993	415.1949	6676.802	7
GWO	6112.193	5892.513	7112.804	416.0391	5909.954	4
TLBO	42,144.27	24,264.99	83,025.09	16,815.72	38,698.07	12
GSA	21,921.33	9777.026	35,302.66	7701.707	21,288.72	10
PSO	42,957.13	16,744.76	75,536.88	15,181.17	46,304.65	13
GA	40,300.82	14,893.63	98,448.02	18,294.20	35,672.07	11

**Table 11:** Performance of optimization algorithms on the speed reducer design problem.

Algorithm	Optimum Variables							Optimum Cost
	*b*	*M*	*p*	*b_1_*	*b_2_*	*d_1_*	*d_2_*	
SOA	3.5	0.7	17	7.3	7.8	3.350215	5.286683	2996.348
WSO	3.5	0.7	17	7.300003	7.800006	3.350215	5.286683	2996.349
AVOA	3.5	0.7	17	7.3	7.8	3.350215	5.286683	2996.348
RSA	3.6	0.7	17	7.3	8.3	3.351345	5.5	3188.612
MPA	3.5	0.7	17	7.3	7.8	3.350215	5.286683	2996.348
TSA	3.502004	0.7	17	7.531361	7.918844	3.353967	5.292829	3006.665
WOA	3.524215	0.700895	17	7.859427	7.957261	3.351278	5.317321	3038.216
MVO	3.503185	0.7	17	7.425817	7.839415	3.374115	5.286934	3005.882
GWO	3.500789	0.7	17	7.468265	7.818522	3.351642	5.286764	2998.965
TLBO	3.575075	0.710376	26.87747	7.412554	7.933867	3.401006	5.308059	5360.781
GSA	3.555741	0.701286	17.26289	8.153121	8.159038	3.443303	5.348242	3150.666
PSO	3.577873	0.703145	17.69495	7.75669	8.048995	3.736336	5.2889	3289.236
GA	3.57042	0.700615	20.37875	8.029033	8.055597	3.433943	5.362534	3758.934

**Table 12:** Statistical results of optimization algorithms on the speed reducer design problem.

Algorithm	Mean	Best	Worst	Std	Median	Rank
SOA	2996.348	2996.348	2996.348	9.33 × 10^−13^	2996.348	1
WSO	2996.845	2996.349	3004.806	1.876691	2996.367	2
AVOA	3000.507	2996.348	3010.109	4.665488	2999.125	4
RSA	3273.628	3188.612	3363.873	60.87604	3258.238	8
MPA	2999.118	2996.44	3002.078	1.841197	2999.338	3
TSA	3038.484	3006.665	3059.817	14.5261	3039.687	7
WOA	3354.082	3038.216	5582.461	653.6465	3113.028	9
MVO	3032.462	3005.882	3058.964	16.87454	3032.846	6
GWO	3004.446	2998.965	3009.552	3.542339	3004.059	5
TLBO	6.67 × 10^13^	5360.781	2.32 × 10^14^	7.04 × 10^13^	4.58 × 10^13^	12
GSA	3498.023	3150.666	4508.259	317.1042	3414.037	10
PSO	1.32 × 10^14^	3289.236	1.32 × 10^15^	2.95 × 10^14^	4.5 × 10^13^	13
GA	6.44 × 10^13^	3758.934	5.09 × 10^14^	1.34 × 10^14^	1.05 × 10^13^	11

**Table 13:** Performance of optimization algorithms on the welded beam design problem.

Algorithm	Optimum Variables				Optimum Cost
	*h*	*l*	*t*	*b*	
SOA	0.20573	3.470489	9.036624	0.20573	1.724852
WSO	0.20573	3.470489	9.036624	0.20573	1.724852
AVOA	0.20573	3.470489	9.036624	0.20573	1.724852
RSA	0.150134	4.75063	10	0.206313	1.979431
MPA	0.20573	3.470489	9.036624	0.20573	1.724852
TSA	0.201353	3.584929	9.045002	0.205782	1.735242
WOA	0.20488	3.546188	8.921686	0.211065	1.754013
MVO	0.203146	3.539276	9.03661	0.205819	1.730769
GWO	0.205462	3.479	9.036729	0.205759	1.72583
TLBO	0.236531	7.25166	7.68074	0.29846	2.791973
GSA	0.192398	4.185734	9.345858	0.219364	1.964869
PSO	0.240686	4.095809	7.000139	0.598026	3.90663
GA	0.133949	6.613311	9.470163	0.396197	3.852019

**Table 14:** Statistical results of optimization algorithms on the welded beam design problem.

Algorithm	Mean	Best	Worst	std	Median	Rank
SOA	1.724852	1.724852	1.724852	6.83 × 10^−16^	1.724852	1
WSO	1.724852	1.724852	1.724854	4.32 × 10^−7^	1.724852	2
AVOA	1.759209	1.725117	1.892483	0.041877	1.74656	7
RSA	2.279139	1.979431	2.751124	0.211874	2.258755	8
MPA	1.726515	1.725441	1.728092	0.000972	1.726346	3
TSA	1.746863	1.735242	1.761533	0.006875	1.746978	5
WOA	2.509499	1.754013	4.629688	0.875301	2.11047	10
MVO	1.749039	1.730769	1.807243	0.017414	1.745069	6
GWO	1.727289	1.72583	1.730001	0.001078	1.727085	4
TLBO	4.32 × 10^13^	2.791973	4.69 × 10^14^	1.24 × 10^14^	5.331066	12
GSA	2.455232	1.964869	2.876107	0.258565	2.391602	9
PSO	7.27 × 10^13^	3.90663	3.02 × 10^14^	1.16 × 10^14^	5.09 × 10^12^	13
GA	3.15 × 10^13^	3.852019	2.37 × 10^14^	7.65 × 10^13^	5.36045	11

**Table 15:** Performance of optimization algorithms on the tension/compression spring design problem.

Algorithm	Optimum Variables			Optimum Cost
	*d*	*D*	*P*	
SOA	0.051689	0.356718	11.28897	0.012665
WSO	0.051689	0.356716	11.28906	0.012665
AVOA	0.051689	0.356718	11.28897	0.012665
RSA	0.05	0.310493	15	0.013196
MPA	0.051688	0.356703	11.28982	0.012665
TSA	0.051504	0.352152	11.57771	0.012683
WOA	0.052121	0.36721	10.69936	0.012669
MVO	0.061321	0.635221	3.976356	0.014275
GWO	0.05171	0.357118	11.27238	0.012674
TLBO	0.069275	0.939724	2	0.018039
GSA	0.05514	0.439926	8.921418	0.014608
PSO	0.068994	0.933432	2	0.017773
GA	0.069308	0.940961	2	0.01808

**Table 16:** Statistical results of optimization algorithms on the tension/compression spring design problem.

Algorithm	Mean	Best	Worst	Std	Median	Rank
SOA	0.012665	0.012665	0.012665	1.19 × 10^−18^	0.012665	1
WSO	0.012683	0.012665	0.012851	4.4 × 10^−5^	0.012666	3
AVOA	0.013316	0.012684	0.014868	0.000655	0.013049	7
RSA	0.026737	0.013196	0.152646	0.033029	0.013333	11
MPA	0.012676	0.012666	0.01269	7.04 × 10^−6^	0.012674	2
TSA	0.012967	0.012683	0.013663	0.00028	0.012883	5
WOA	0.013266	0.012669	0.01558	0.000739	0.013041	6
MVO	0.017716	0.014275	0.018434	0.000985	0.017988	8
GWO	0.012769	0.012674	0.013195	0.00012	0.012727	4
TLBO	0.018633	0.018039	0.019221	0.000332	0.018711	9
GSA	0.019185	0.014608	0.036067	0.004735	0.017658	10
PSO	1.99 × 10^13^	0.017773	3.97 × 10^14^	8.88 × 10^13^	0.017773	13
GA	3.38 × 10^12^	0.01808	5.31 × 10^13^	1.19 × 10^13^	0.026219	12

## Data Availability

Not applicable.
